# Species delimitation in *Amblyosyllis* (Annelida, Syllidae)

**DOI:** 10.1371/journal.pone.0214211

**Published:** 2019-04-10

**Authors:** María Teresa Aguado, María Capa, Domingo Lago-Barcia, João Gil, Fredrik Pleijel, Arne Nygren

**Affiliations:** 1 Centro de Investigación en Biodiversidad y Cambio Global (CIBC-UAM), Departamento de Biología, Facultad de Ciencias, Universidad Autónoma de Madrid, Cantoblanco, Madrid, Spain; 2 Animal Evolution and Biodiversity, Johann-Friedrich-Blumenbach Institute for Zoology & Anthropology, Georg-August-Universität Göttingen, Göttingen, Germany; 3 Departament de Biologia, Universitat de les Illes Balears, Palma, Illes Balears, Spain; 4 NTNU University Museum, Norwegian University of Science and Technology, Trondheim, Norway; 5 Laboratório de Ecologia e Evolução, Escola de Artes, Ciências e Humanidades (EACH), Universidade de São Paulo, São Paulo, São Paulo, Brazil; 6 Centre of Marine Sciences, CCMAR, University of Algarve, Faro, Portugal; 7 Centre d’Estudis Avançats de Blanes, CEAB-CSIC, Blanes, Girona, Spain; 8 Department of Marine Science, The Faculty of Science, University of Gothenburg, Tjärnö, Strömstad, Sweden; 9 Sjöfartsmuseet Akvariet, Gothenburg, Sweden; Universidad de Sevilla, SPAIN

## Abstract

*Amblyosyllis* is a worldwide distributed group of annelids mainly found in coastal environments. It is well known among the polychaete specialists mostly because of its notable beauty, showing bright colourful patterns and outstanding long and coiled appendices. *Amblyosyllis* is a monophyletic genus easy to identify due to its distinct diagnostic features; however, the species and their boundaries are, in most cases, not well defined. Herein, we provide an extensive sample of *Amblyosyllis* material (115 specimens) from several world geographic areas. We have studied the morphological features of each specimen and photographed them alive. Two mitochondrial DNA markers (COI and 16S) and one nuclear gene fragment (28S, D1 region) were sequenced. We performed phylogenetic analyses based on each DNA partition, as well as the combined data sets, obtaining congruent results. Species delimitation methods such as distance analyses, statistical parsimony networks and multi-rate Poisson tree processes were also applied. The combined results obtained from different methodologies and data sets are used to differentiate between, at least, 19 lineages compatible with the separately evolving meta-populations species concept. Four of these lineages are identified as nominal species, including the type species of *Amblyosyllis*, *A*. *rhombeata*. For three other lineages previously synonymized names are recovered, and seven lineages are described as new species. All of these species are described and supported by appropriate iconography. We recognize several morphological characters useful to identify species of *Amblyosyllis*, which in some cases should also be combined with molecular methods for species delineation. The genetic divergence in the genus is high, contrary to the morphological homogeneity observed. Two species show a wide geographical distribution, while the rest have a more restricted distribution. There are several examples of species with overlapping distribution patterns.

## Introduction

“They were very beautiful creatures, and had a habit of curling their rosy tentacular filaments into regular compact spirals” (Mr. A. Hancock about *Amblyosyllis spectabilis*, September 1850, [1]).

**Fig 1:**
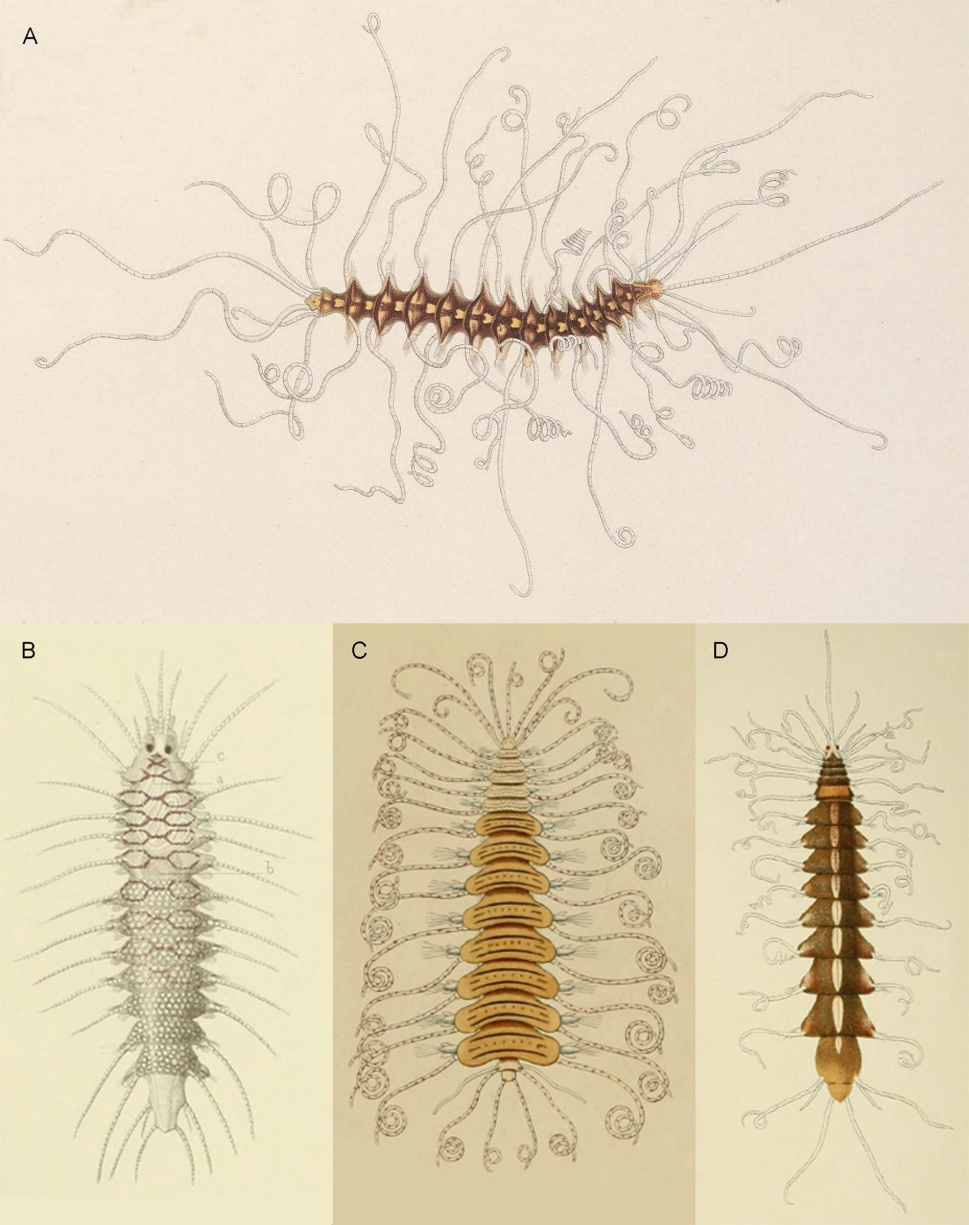
*Amblyosyllis* drawings from early polychaete literature. (A) *Amblyosyllis lineata*, by McIntosh [7]. (B) *Pterosyllis dorsigera*, by Claparède [5]. (C) *Nicotia lineolata*, by Costa [6]. (D) *Amblyosyllis speciosa*, by Izuka [3].

Indeed, members of *Amblyosyllis* Grube & Ørsted *in* Grube, 1857 [2] are remarkable creatures, well known among the polychaete specialists mostly because of their notable beauty; which has been used several times to name species (e.g. *A*. *speciosa* Izuka, 1912 [3] or *A*. *formosa* (Claparède, 1863) [4], from Latin “specious” and “formosus”, respectively, meaning beautiful and finely formed), and has made them a usual presence on marine life guides. They usually exhibit colourful patterns and have outstanding long and coiled appendices. *Amblyosyllis* are part of a group of small benthic marine worms, the syllids (Annelida, Syllidae) that inhabit practically every region of the ocean. Grube and Ørsted erected *Amblyosyllis* in 1857 and since then several species have been described worldwide. There are detailed and gorgeous illustrations in the old literature, such as those by Claparède [5], Costa [6], Izuka [3] or McIntosh [7] (Fig 1).

Aguado *et al*. [8–10] included members of *Amblyosyllis* in phylogenetic analyses with molecular data for the first time and showed the group to be monophyletic and closely related to subfamilies Eusyllinae or Autolytinae. Their morphological similarities place them close to *Brachysyllis* Imajima & Hartman, 1964 [11] and/or *Dioplosyllis* Gidholm, 1962 [12, 13, 14], although these two latter genera have never been included in a molecular phylogenetic analysis.

*Amblyosyllis* is a morphologically homogeneous group, easy to identify due to its distinct and exclusive morphological characteristics such as: long and coiled dorsal cirri, trapezoidal segments, a long, thin and coiled pharynx armed with a ring of anterior denticles called trepan, supposedly used to pierce pieces of food, and external nuchal appendices for chemoreception named nuchal lappets [15]. However, the features that probably make it unique within the Syllidae are the reduced and constant number of chaetigers (13), and the presence of an achaetous segment anterior to the pygidium [15]. These features are only shared by members of *Brachysyllis* (which in addition is distinguished from *Amblyosyllis* by the presence of cylindrical segments, a straight pharynx, a middorsal pharyngeal tooth and the lack of nuchal lappets). The rest of syllids do not show a specific and invariable number of segments [13], and many are supposed to grow during their postembrionary development by adding posterior segments through the action of a segment addition zone, located just in front of the pygidium [16]. Members of *Amblyosyllis* are able to regenerate posteriorly, but the number of newly formed segments never exceeds the original [17].

*Amblyosyllis* species have been found in the intertidal and subtidal zones (0–200 m), on a large variety of substrates and in association with different organisms (rocky shores, coarse sand, algae, ascidians, bryozoans, sponges, mollusks, etc.) (MTA pers. obs., [18, 19]). Very little is known about the diet of most syllids [20, 21], but some members of *Amblyosyllis* have been observed feeding on sponges [22, 23]. Pernet [23] was able to culture an *Amblyosyllis* species, identified as *A*. *speciosa* from Friday Harbor (Washington, USA; but see Taxonomic section), in laboratory conditions and observed and documented its reproduction. Members of this species are dioecious and epigamous (reproductive individuals with posterior segments full of gametes and enlarged eyes), the fertilization is external, females brood egg masses (during at least five consecutive reproductive processes), and the juveniles develop without free-living larval previous stages [23]. This combination of exceptional and distinctive biological features (morphological constrains and reproductive mode) would make these animals a suitable model organism for evolutionary biology, ecological or developmental studies. However, our knowledge is still very limited, especially their species diversity.

While the genus is well defined, the species boundaries are not clear [18, 19, 24, 25]. Nominal species have been described based on unique combinations of few features that often show little disparity among members of the group. Intermediate forms reported in literature have been either assigned to new species, or contrary, have been assumed to represent variation within a particular species causing, in some cases, lumping and synonymisation of species. This latter solution results in some of the nominal species showing broad geographic distributions. The database WoRMS [26] currently accepts ten species and two subspecies of *Amblyosyllis* as valid, one more is listed as *nomen dubium*, and nine others are considered junior synonymies (Table 1), most of them of *A*. *formosa*. Five species have a wide distribution (Table 1), being reported from very distant areas (e.g. *A*. *formosa* has been cited in the Mediterranean, Atlantic, South Africa, or Australia) [18, 27, 28].

**Table 1:** Valid species [26], geographic distribution and synonymies.

Genus	Type locality	Reported Distribution	Original description and additional references	Synonymies (organized chronologically)	Source of synonymy
*Amblyosyllis* Grube & Ørsted *in* Grube, 1857	St. Croix Is., Virgin Is., USA	Cosmopolitan	[2, 18]	*Gattiola* Johnston *in* Baird, 1861 *Pterosyllis* Claparède, 1863 *Nicotia* Costa, 1864 *Thylaciphorus* Quatrefages, 1865 *Pseudosyllides* Czerniavsky, 1882	[29, 30]
**Species (chronological order)**					
* *A*. *rhombeata* Grube & Ørsted *in* Grube, 1857	Virgin Is., USA	Caribbean Sea	[2, 31]		
* *A*. *formosa* (Claparède, 1863)	Normandy, France	Cosmopolitan	[4, 18]	* *Gattiola spectabilis* Johnston *in* Baird, 1861 * *Amblyosyllis lineata* Grube, 1863 *Pterosyllis dorsigera* Claparède, 1864 *Thylaciphorus hessii* Quatrefages, 1865 *Pterosyllis plectorhyncha* Marenzeller, 1874 *Amblyosyllis immatura* Langerhans, 1879 *Amblyosyllis algefnae* Viguier, 1886	[18, 32]
* *A*. *finmarchica* (Malmgren, 1867)	Finnmark, Norway	NE Atlantic (Norway; Iceland); NW Atlantic (Labrador—Maine); Pacific (N Japan Sea); Arctic (Russia)	[33–36]		
*A*. *cincinnata* (Verrill, 1874)	Maine, USA	NW Atlantic (Maine)	[22, 37, 38]		
* *A*. *madeirensis* Langerhans, 1879	Madeira Is., Portugal	Circum-tropical	[18, 29]		
*A*. *granosa* Ehlers, 1897	Punta Arenas, Chile	S Pacific (Chile; New Zealand); Central Pacific (Panamá; Galapagos Is.); S Indian (Western Australia; Kerguelen Is.); SW Atlantic (Tristan da Cunha; Brazil)	[39–41]		
*A*. *speciosa* Izuka, 1912	Osaka Prefecture, Japan	E–W Pacific (Japan; California)	[3, 11, 42]	*Amblyosyllis nigrolineata Okada, 1934	[11, 30]
*A*. *lineata alba* Berkeley, 1923	Vancouver Is., Canada	E Pacific (Canada)	[43, 44]		
*A*. *formosa corallicola* (Hartmann-Schröder, 1960)	Egypt, Red Sea	Red Sea (Egypt)	[45, 46]		
*A*. *vesiculosa* Hartmann-Schröder, 1989	New South Wales, Australia	W Pacific (New South Wales)	[47]		
*A*. *multidenticulata* San Martín & Hutchings, 2006	New South Wales, Australia	W Pacific (New South Wales)	[19]		
*A*. *enigmatica* San Martín & Hutchings, 2006	New South Wales, Australia	W Pacific (New South Wales; Queensland); SE Indian (Western Australia)	[19]		
**Nomen dubium**					
*A*. *lineolata* (Costa, 1864)	Naples, Italy	Mediterranean Sea (France; Italy); South Africa	[6, 48, 49]	*Cirrosyllis picta* Schmarda, 1861	[49]

*Amblyosyllis* valid species and synonymies. Information obtained from the data base WoRMS [26]; authorships and publication dates corrected according to the present work, excepting distribution of *A*. *granosa* [50] and *A*. *finmarchica* [36, 51, 52]. Note that the type locality of *A*. *finmarchica* is Finnmark, in northern Norway [33], not in Finland as [35], and followed elsewhere (e.g. [26]).

*: Species included in this study.

Cosmopolitan species, identified based only on morphology, have been shown to be, in many cases, complexes of cryptic or pseudocryptic species [53–56]. Cryptic species are two or more morphologically similar species that have been erroneously classified as a single one [57], while pseudocryptic species are those that can be morphologically distinguished, but only after other methods have revealed their existence [53, 54]. In general, species delimitation is a common difficulty in annelid systematics [57]. However, there is currently access to a variety of methods specifically designed to establish species delimitations. Apart from morphological features, DNA sequence data have been widely used for inferring species limits and phylogenetic relationships, also in annelids (e.g. [58–64]). For instance, Nygren & Pleijel [62] used molecular markers (COI and ITS) to uncover ten cryptic species within the *Eumida sanguinea* (Ørsted, 1843) [65] species complex. On the other hand, the same markers were also used by Nygren *et al*. [64], who found that the supposedly *Harmothoe imbricata* (Linnaeus, 1767) [66] species complex was in fact a single species with at least 10 different colour morphotypes in north-east Atlantic populations. In Syllidae, Maltagliati *et al*. [67] and Westheide & Hass-Cordes [68], were the first ones including molecular information, apart from morphology, to investigate the species complexes problem in *Syllis gracilis* Grube, 1840 [69] and in the genus *Petitia* Siewing, 1956 [70], respectively. The phylogenetic analyses based on molecular markers [8, 9] pointed out that some worldwide distributed species, usually identified by few distinct and conspicuous characters, (e.g. *Branchiosyllis exilis* (Gravier, 1900) [71], *Haplosyllis spongicola* (Grube, 1855) [72], *Syllis armillaris* (Müller, 1776) [73], *S*. *gracilis*, *S*. *hyalina* Grube, 1863 [74], and *Trypanosyllis zebra* (Grube, 1860) [75]) could be cryptic or pseudocryptic species, in need of revision, some of them in agreement with the findings of some previous morphological analyses (e.g. [76]). Recently, Álvarez-Campos *et al*. [77, 78] dealt with *Syllis gracilis* and *Trypanosyllis* Claparède, 1864 [5] species delimitation conflicts. Nevertheless, comprehensive genetic studies of species boundaries in marine annelids, including Syllidae, are still scarce [57].

As stated above, the identification of *Amblyosyllis* species up to now has been only based on morphological characters, which in many cases are not clear enough and rely on a certain degree of subjectivity. This is mainly because differences between species are scarce and intraspecific variation often overlaps with interspecific differences. Therefore, as previously stated, the genus is in need of a revision to assess the species boundaries, and test their diagnostic features and their current geographical and bathymetrical distribution. Herein, we provide an extensive sample of *Amblyosyllis* specimens (115), from several geographic areas (16 ecoregions *sensu* Spalding *et al*., [79]). The morphological features traditionally used to discriminate species are evaluated. Three different molecular markers have been sequenced (the mitochondrial COI, and 16S, and the nuclear 28S) and used to perform phylogenetic analyses (Maximum likelihood and Bayesian inference) to discern their evolutionary relationships. Furthermore, different molecular species delimitation methods (genetic distances, statistical haplotype networks, multi-rate Poisson Tree Processes) were applied, and all the collected information was integrated and used to establish the species boundaries. Finally, we describe and identify two of the most well known species of the genus: *A*. *madeirensis* Langerhans, 1879 [29] and *A*. *spectabilis* (Johnston *in* Baird, 1861) [80]. The latter had previously been considered a junior synonym of *A*. *formosa*, but we found that it has priority since it was described earlier (see Taxonomic Account section). *Amblyosyllis rhombeata* Grube & Ørsted *in* Grube, 1857 [2], the type species of the genus, and *A*. *finmarchica* (Malmgren, 1867) [33] are also redescribed. Additionally, we recovered three previously synonymised names from the literature: *A*. *lineata* Grube, 1863 [74], *A*. *plectorhyncha* (Marenzeller, 1874) [81], and *A*. *nigrolineata* Okada, 1934 [82]. Seven new species are described (*A*. *antoni* n. sp., *A*. *ovei* n. sp., *A*. *clarae* n. sp., *A*. *emilioi* n. sp., *A*. *hectori* n. sp., *A*. *anae* n. sp. and *A*. *idae* n. sp.); and finally, five more species are described but not formally named because there is not enough material available to enable a complete description (*Amblyosyllis* sp. 1–5).

## Materials and methods

### Collection and morphological study

**Fig 2:**
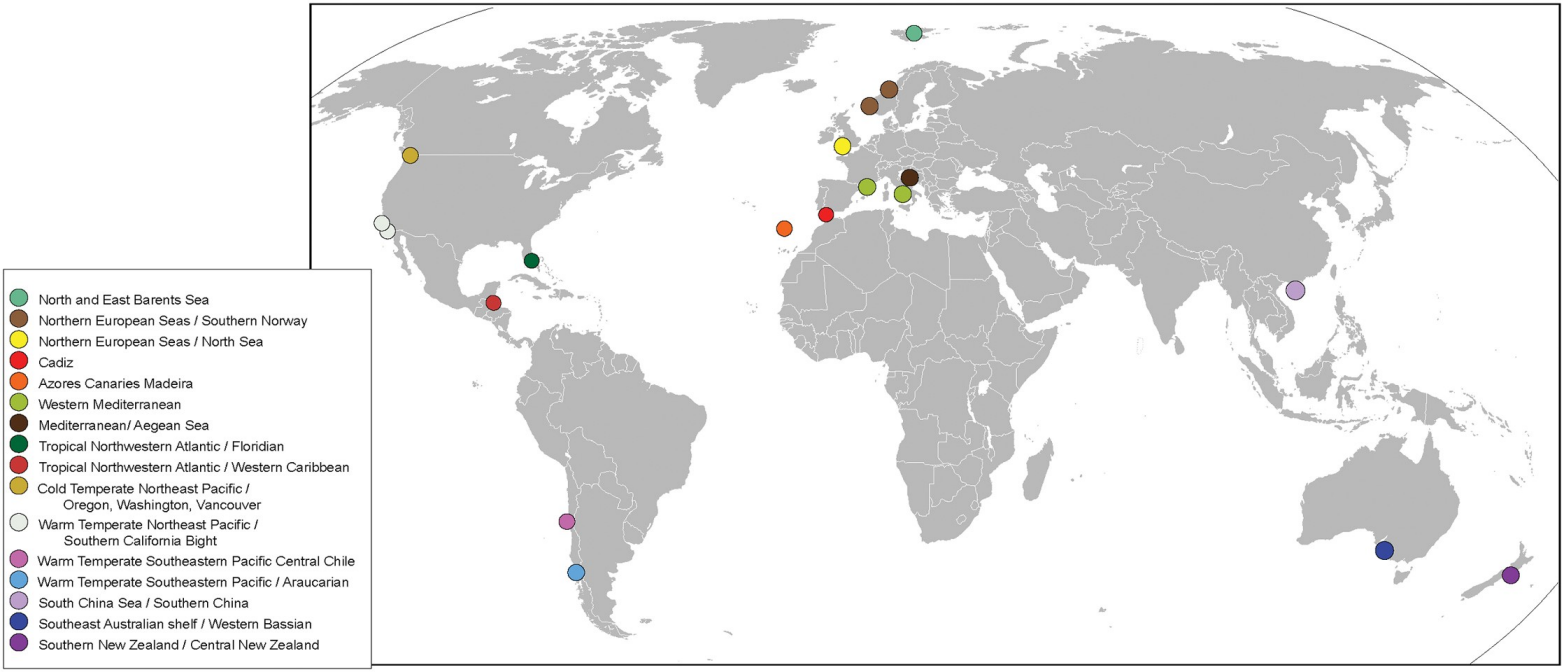
Map with sampling localities. Bioregions from Spalding *et al*. [79].

A total of 115 specimens of *Amblyosyllis* were collected from several worldwide localities. Biogeographic realms, provinces and ecoregions were considered (as per Spalding [79]), and are indicated in Fig 2 (see also S1 File).

Most of the samples were collected from shallow waters, except those from Norway that are from 100–250 m depth (S1 File). No material has been found from deeper depths. Individuals were collected from different substrates ranging from rocky shore or coralline to mangrove roots. The specimens were found together with other organisms such as sponges (*Geodia*, *Phakellia*), mussels, corals (*Lophelia*), algae (*Caulerpa*), ascidian tunicates (*Polycarpa*), other polychaetes (e.g. *Sabella* and *Chaetopterus*), barnacles and plants (*Phyllospadix*) (See S1 File). Most of the examined samples are from warm to tropical ecoregions, except those coming from Northern European Seas, North and East Barents Sea, Cold Temperate Northeast Pacific, and Southern New Zealand / Central New Zealand (Fig 2 and S1 File).

All samples were collected prior to that the Nagoya protocol entered into force, thus there was no need for specific permissions. Sampling did not include endangered or protected species. The specimens were observed and photographed *in vivo*. Four specimens were fixed in formalin, and later changed to 70% ethanol for further morphological studies. Other specimens (111) were immediately preserved in 100% ethanol and maintained at 4°C, for DNA sequencing. The morphology of the different specimens was also studied in the laboratory using a compound microscope with interference contrast optics (Nomarsky) and photographs of parapodia, pharynges and trepans were taken. For scanning electron microscopy (SEM), the specimens were critical point dried with an Emitech K850 Critical Point Dryer, gold-coated with a Q150T-S Turbo-Pumper Sputter Coater, and examined with a Hitachi S-3000N electron microscope at Servicio Interdepartamental de Investigación (SIDI), Universidad Autónoma de Madrid (UAM). Width of the specimens, excluding parapodia, was measured at the proventricle level. Some anterior ends were dissected and others processed with winter green and lactic acid for making the soft tissues transparent to observe the trepan. Studied specimens, voucher numbers, and details of each sample can be consulted in S1 File. Additional comparative material from different institutions (MNCN: Museo Nacional de Ciencias Naturales de Madrid; SMNH: Naturhistoriska Riksmuseet, Stockholm; MfN: Museum für Naturkunde, Berlin) was also revised and morphological features were checked and compared.

### DNA extraction, amplification and sequencing

Genomic DNA from the posterior end of 109 specimens was extracted using standard protocols. Partial mitochondrial genes from the cytochrome oxidase subunit 1 (COI) and 16S, and partial nuclear gene 28S, were sequenced. Polymerase chain reaction (PCR) amplification consisted of an initial denaturation at 96 °C for 1 min, followed by 35 cycles of denaturation at 95 °C for 30 seconds, annealing at 50 °C for 30 seconds and extension at 72 °C for 1 min, with a final extension of 8 min at 72 °C. To obtain the COI sequences the primers LCO1498, HCO2198, COIE, POLY LCO and POLY HCO [52, 83, 84] were used; for the 28S sequences, the region D1 was sequenced and the primers 28SC1 and 28SC2R [85] were used; for the 16S sequences, the primers 16SARL, 16SBRH and 16SANNF [86, 87] were used. The products of successful amplification were purified using ExoSAP-IT PCR Product Cleanup protocol (ThermoScientific). Sanger sequencing was performed on both strands at Eurofins Genomics, DNA Sequencing Department in Ebersberg, Germany.

*Epigamia magna* (Berkeley, 1923) [43] from the subfamily Autolytinae Langerhans, 1879 [29] and *Eusyllis blomstrandi* Malmgren, 1867 [33] from the subfamily Eusyllinae Malaquin, 1893 [88] were used as outgroups. GenBank accession numbers for each individual and genes can be found in S1 File. Additionally, the sequences of COI and 16S obtained for this study have been blasted and compared with those already in the GenBank (see remarks of the species *A*. *finmarchica* and *A*. *nigrolineata* in the Taxonomic account section).

### Phylogenetic analyses

Alignments of each marker were performed separately using the program Mafft [89, 90] with the default parameters and the iterative refinement method E-INS-i, and default gap open and extension values. The alignments were revised using BioEdit [91]. We analysed the mitochondrial data set independently (COI and 16S), the combined mitochondrial data set (COI+16S), the nuclear data set (28S), as well as the complete DNA combined data set (COI+16S+28S) with Maximum likelihood (ML). The combined data matrixes (COI+16S and COI+16S+28S) were also analysed with Bayesian inference (BI) optimality criteria. The genes were combined using FASconCAT-G [92, 93]. ML analyses were implemented in IQ-TREE [94, 95] and best fitting models for each partition were selected by the same program (COI: TIM3+I+G4; 16S: GTR+G4; 28S: TN+G4). Each partition was allowed to have its own set of branch lengths (-sp option). Support values were estimated based on 1000 bootstrap pseudoreplicates (B). For BI analyses, two independent runs of 1000000 generations and four chains, each (one cold, three heated) were run in MrBayes 3.2.2 [96] and trees were sampled every 1000 generations. The most similar models available in MrBayes (-mset option) to those selected by IQ-TREE for each partition were applied. All parameters were unlinked, and rates were allowed to vary freely over partitions. Burn-in and parameter/run convergence were assessed using TRACER v. 1.5 [97]. After discarding 50000 first trees as burn-in, trees from the stationary phase were combined to obtain a majority rule consensus and posterior node probabilities [98].

### Genetic distances and species delimitation

Nucleotide divergence (p-distance and best fitting substitution model, S3–S5 Files) over sequence pairs within and between the well supported lineages after the phylogenetic analyses (19 groups, see phylogenetic results) was estimated in MEGA v.7 [99]. Positions containing gaps and missing data were removed. Two single-locus sequence-based species delimitation methods were applied: identification of independent networks using statistical parsimony [100] and analysis of branch pattern dynamics to detect independently evolving entities (i.e. putative species) using the multi-rate Poisson Tree Processes model (mPTP [101]). The frequency of haplotypes was calculated with TCS v. 1.21 [102]. Statistical parsimony haplotype networks were calculated using TCS (calculating 95% connection limit and treating IUPAC ambiguity codes as missing data) of COI, 16S and 28S aligned sequences independently. Alignment files were trimmed to minimise the amount of missing data. Results were visualized using PopART (population Analysis with Reticulate Trees [103]), grouping sequences in the putative 19 species after phylogenetic analyses and distinguished morphotypes.

The resulting ML tree from the analysis of COI sequences was the input tree for mPTP. The mPTP model attempts to identify the shifting point from the speciation to the coalescent processes, assuming that the two processes produce phylogenies of distinct branching patterns which can be described by a two or more parameter model. Similar analyses were performed with 16S and 28S sequences. Problems with singletons and short branches in this kind of method have been previously detected (e.g. [104]) and hence we also performed analyses using PTP [105], considering only a two parameter model, using the COI ML tree as input (as well as in mPTP) and the ML tree obtained from the combined data set (mitochondrial and nuclear markers) (see S6 File).

## Results

### Morphological features

The purpose of our morphological studies was to review all the characteristics mentioned in the previous descriptions in order to establish which ones could be useful for species identifications. Because of this, after the morphological studies, we have divided the morphological features traditionally used in the descriptions of *Amblyosyllis* into three groups: a) Invariable, b) With intraspecific variability, and c) With interspecific variability. The latter is here proposed to distinguish between the 16 possible morphospecies we have recognised (Table 2), as well.

**Table 2:** Morphospecies proposal and interspecific features.

Morpho-species	Clade	Nuchal lappets	Colour pattern	Colour pattern (per segment)	Trepan teeth	Chaetae	Distal vs proximal chaetal teeth size	Chaetae length	Occurrence
A	1	absent/reduced	distinct	dark transversal bands	8 pentacuspid	bidentate	equivalent	long	France
B	2	present	distinct	spots and lines		bidentate	distal>>proximal	short	New Zealand
*C	3	present	distinct	white longitudinal line		bidentate	equivalent	short	South Australia
*C	4	present	distinct	white longitudinal line		bidentate	equivalent	medium	New Zealand
D	5	present	distinct	transversal lines	6 multicuspid	unidentate	proximal minute or absent	short/ medium	Norway
E	6	present	distinct	large spots	? 6 pentacuspid	bidentate	equivalent or distal>proximal	long	Florida (USA) Belize
F	7	present	distinct	anterior dark area and posterior dark transversal line		bidentate	equivalent	short	Hong Kong
*G	8	present	variable	spots and lines; anterior segments with dark areas and central region unpigmented	? 6 pentacuspid	bidentate	equivalent	long/medium	France, Croatia, Madeira (Portugal)
*H	9	present	variable	spots and lines	6 pentacuspid	bidentate	equivalent	long	Norway
*G	10	present	variable	dark transversal lines and areas; dark areas with central region unpigmented		bidentate	equivalent	long	France, Croatia, Italy
*I	11	present	distinct	dark transversal lines		bidentate	distal>>proximal	medium to short	Chile
*I	12	present	distinct	dark transversal lines	6 pentacuspid	bidentate	distal>>proximal	medium to short	Chile
J	13	present	distinct	dark transversal lines		bidentate	distal>proximal	long/ medium	Florida, Belize
K	14	present	distinct	dark transversal lines		bidentate	distal>proximal	long/medium	California (USA)
L	15	present	distinct	dark transversal lines	6 pentacuspid	bidentate	distal>>proximal	short	California (USA)
M	16	present	distinct	anterior dark area and posterior dark transversal line	? 6 pentacuspid	bidentate	distal>>proximal	short	California (USA)
N	17	present	distinct	dark lines/bands		bidentate	distal>proximal	long	Croatia
O	18	present	distinct	dark transversal lines	? 6 pentacuspid	bidentate	equivalent or distal>proximal	long	France, Italy
*P	19	present	variable	dark transversal lines and ∞ pattern	6 pentacuspid	bidentate	distal>>proximal	long	France, Italy, Croatia, Great Britain, Madeira (Portugal)

Morphospecies proposal and interspecific features. The upper most chaetae in the midbody fascicles have been considered for measurements of blade’s length. Long blades are >40 μm length; medium blades are around 40 μm length; and short blades are <40 μm length. Possible cryptic or pseudocryptic species considering only morphology with an asterisk (*).

#### a) Invariable morphological features

**Fig 3:**
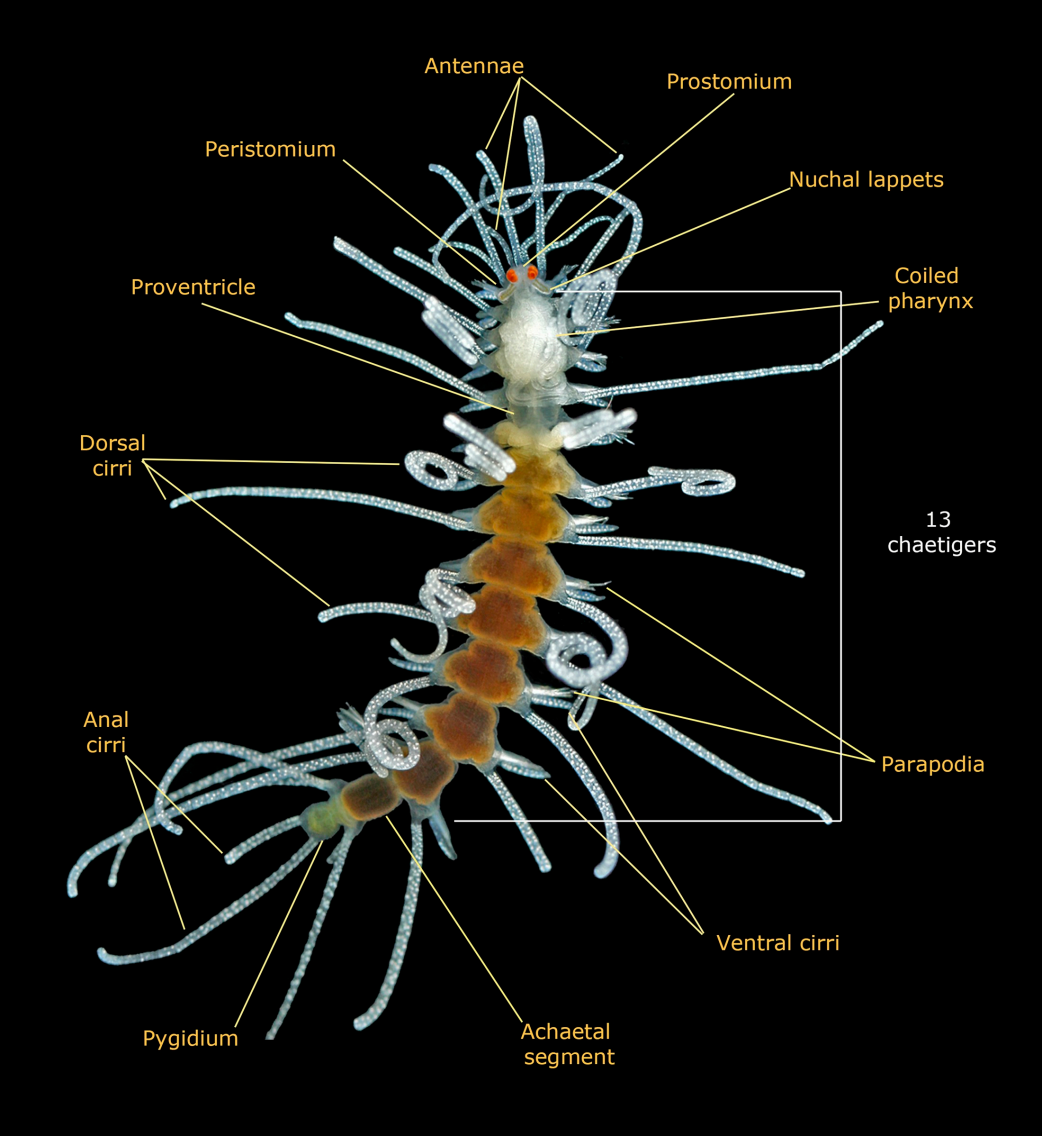
Invariable characters in *Amblyosyllis*. Photo: *Amblyosyllis clarae* n. sp. MNCN 16.01/17994.

Features defining the genus and allowing it to be distinguished from other syllid genera (Fig 3). The invariable features are:

- 13 chaetigers (fixed number).
- One achaetous prepygidial segment.
- Palps ventrally folded.
- Segments with trapezoidal shape.
- Pharynx long (as long as the body length *sensu* Riser [22]), thin and convoluted, occupying the anterior segments.
- Proventricle (i.e. muscular differentiated portion of the digestive tube present in all syllids) barrel-shaped, usually as long as one or two segments
- Dorsal cirri long (often as long as or longer than body length) and coiled (in live specimens).
- Dorsal and ventral cirri with small spherical glands. Those inside dorsal cirri usually organized in longitudinal bands.
- Dorsal cirri often smooth basally while distally pseudoarticulated.
- Presence of one long and conical papilla located dorso-distally on parapodia.
- Four to five straight and pointed aciculae in midbody parapodia

#### b) Morphological features showing intraspecific variability

Some of these features may appear only in certain moments of the life cycle (e.g. during reproduction). These are:

- Secondarily annulated segments.
- Presence of bunches of cilia organized in transversal lines over the dorsal and/or ventral sides of segments.
- Presence of pores and secretions in dorsal and ventral cirri.
- Postventricular segments (last eight chaetigers) modified for reproduction in epigamic specimens, with the segments immediately after the proventricle being full of gametes and enlarged in size (being eventually massive).
- Ventral cirri showing some variation in shape, from lanceolate and distally pointed to digitiform and distally rounded. However, these are always slight variations that can be modified in the preserved material.

#### c) Features showing interspecific variability

**Fig 4:**
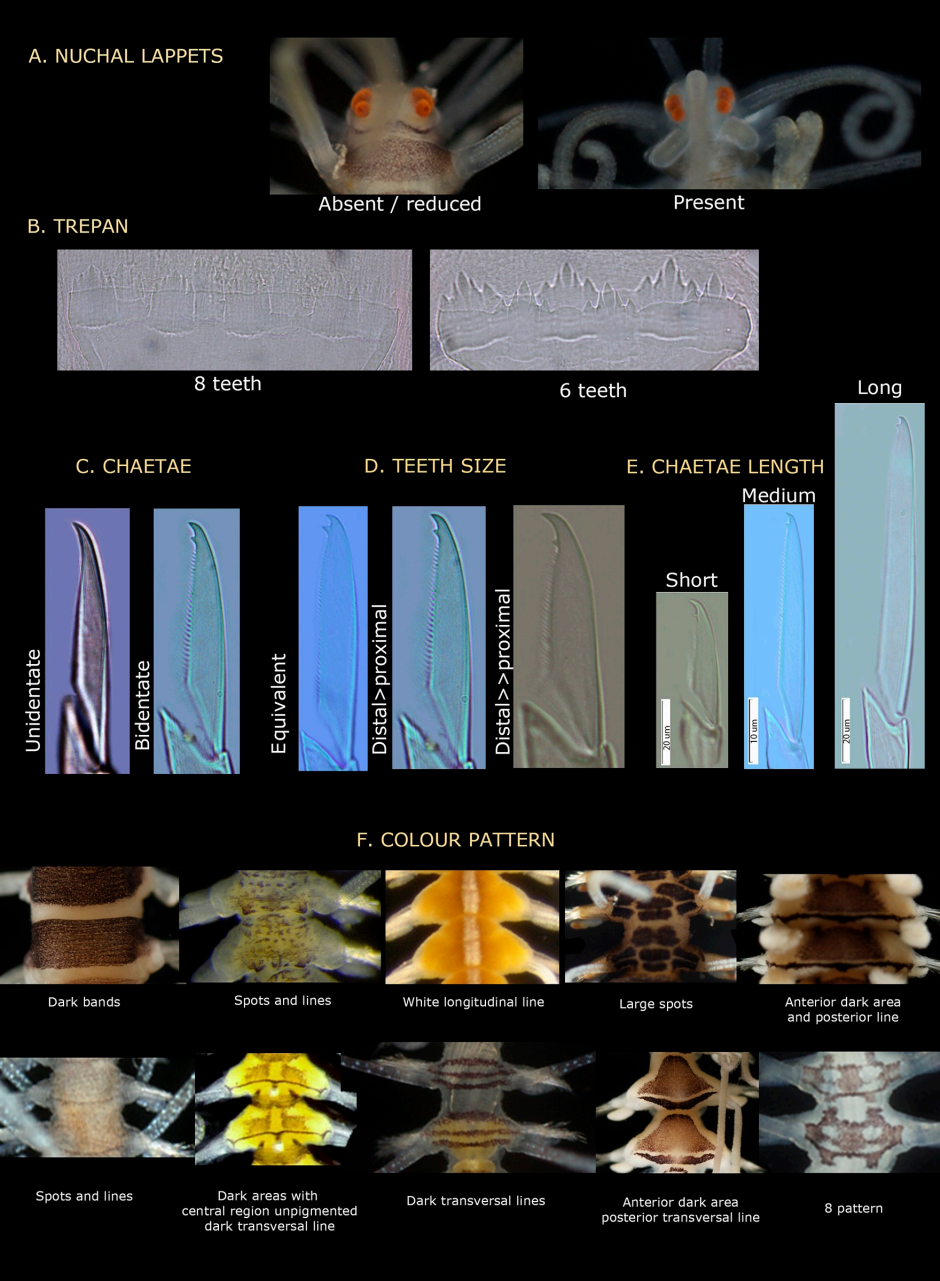
Interspecific variable characters in *Amblyosyllis*. (A) Nuchal lappets. (B) Trepan. (C) Chaetae. (D) Chaetal teeth relative size. (E) Chaetal length. (F) Colour patterns.

- Presence/absence of nuchal lappets arising dorsally from the limit between the prostomium and the first segment. They are backwards oriented and usually long though not exceeding the length of the first segment (Fig 4). They are generally present, but we have found specimens (from Banyuls, France) without them or appearing reduced and similar to ciliary grooves. The length of the nuchal lappets (up to second chaetiger) and their shape (rounded or cirriform) have also been used to differentiate species [18]. According to our observations, these two latter features are often variable and not useful as a species diagnostic character.

- Colour pattern. This characteristic has been traditionally used to discriminate species (e.g. [5, 29, 106, 107]). However, it has also been considered variable within certain species [18, 19]. Additionally, colour is completely lost or considerably faded in preserved material. Thus, we only considered the pigmentation pattern for live specimens. To establish the different colour patterns, the following situations have to be taken into account: a) epigamic specimens modify their colouration in the postventricular segments, and b) the gut content can as well change the body ground colour, which is mostly yellow or cream (e.g., see Remarks section in the species *A*. *spectabilis*). Among the studied material, it was possible to discern 10 clear different colour patterns (Fig 4, Table 2). Some of them are unique and distinctive of certain morphospecies (defined by other features); however, in some morphospecies this feature was variable (Table 2); and in other cases, same colourmorphs are shared by diferent morphospecies (Table 2). This character is therefore useful to differentiate species in combination with other features.
- Number and shape of teeth in the trepan. The trepan is a very difficult structure to observe. It is quite small in size (usually no more than 0.8 mm in length) and its observation requires the dissection of the specimen or a treatment to make the tissues transparent. Most of the trepans studied here were composed by a distal ring of six teeth, though they can be eight in some cases (Fig 4, Table 2). The teeth are normally pentacuspid, though they may also show numerous small cusps (e.g see *A*. *finmarchica*). The number of cusps is usually very difficult to discern clearly in the preserved material. In some cases, if the visualization is not clear enough, two teeth in the same trepan may look like different (e.g. see *A*. *spectabilis*). We propose that the number of teeth is relevant for species identification, but not the shape of the teeth.
- Chaetae shape. The chaetae of *Amblyosyllis* are compound, heterogomph and mostly bidentate. However, some species, such as *A*. *finmarchica* and *A*. *cincinnata* (Verrill, 1874) [37] (Table 1) were described with unidentate chaetae. We have found specimens from Svalbard (Norway), assigned to *A*. *finmarchica*, with unidentate or slightly bidentate chaetae (with a minute proximal teeth) (Table 2, Fig 4). Chaetae show in all *Amblyosyllis* fine spinulation on the verge of blades and dorso-ventral gradation in length (i.e. the dorsalmost are longer and the length decreases gradually to the ventralmost ones, which are the shortest). This way, blades in a midbody chaetiger can be long (more than 40 μm), medium length (around 40 μm), or short (less than 40 μm) (Fig 4). Their shape and length have been considered as practically invariant within the genus [18], but we found some differences between morphospecies. It is interesting to note that blades are usually longer in anterior chaetigers than in midbody or posterior ones. The relative shape of the distal and proximal teeth in the bidentate chaetae shows, as well, some variation. We have considered three different states (Fig 4): distal and proximal teeth approximately equivalent, distal tooth larger than proximal one, and distal tooth much larger (approximately the double) than proximal one. Summarizing, the possible interspecific features to be considered regarding chaetae are: number of distal teeth in blades, length of blades, and relative size of distal teeth (Fig 4).

### Morphospecies

As a result of the complete morphological study, we consider that the following combination of features can be used to discriminate morphospecies: presence of nuchal lappets, colour pattern (when distinct), trepan (when seen) and chaetae (monodentate/bidentate, length, and the relative size of distal teeth). Considering these features, we found 16 possible morphospecies in our material (Table 2). This is a preliminary hypothesis to be compared with the results obtained from the analyses of molecular data (phylogenetic, genetic distances and sequence-based species delimitation results).

Morphospecies A–F and I–O show a unique combination of features that have been considered as diagnostic. In contrast, morphogroups G, H and P show variability regarding some of the selected characters (Table 2).

### Phylogenetic results

**Fig 5:**
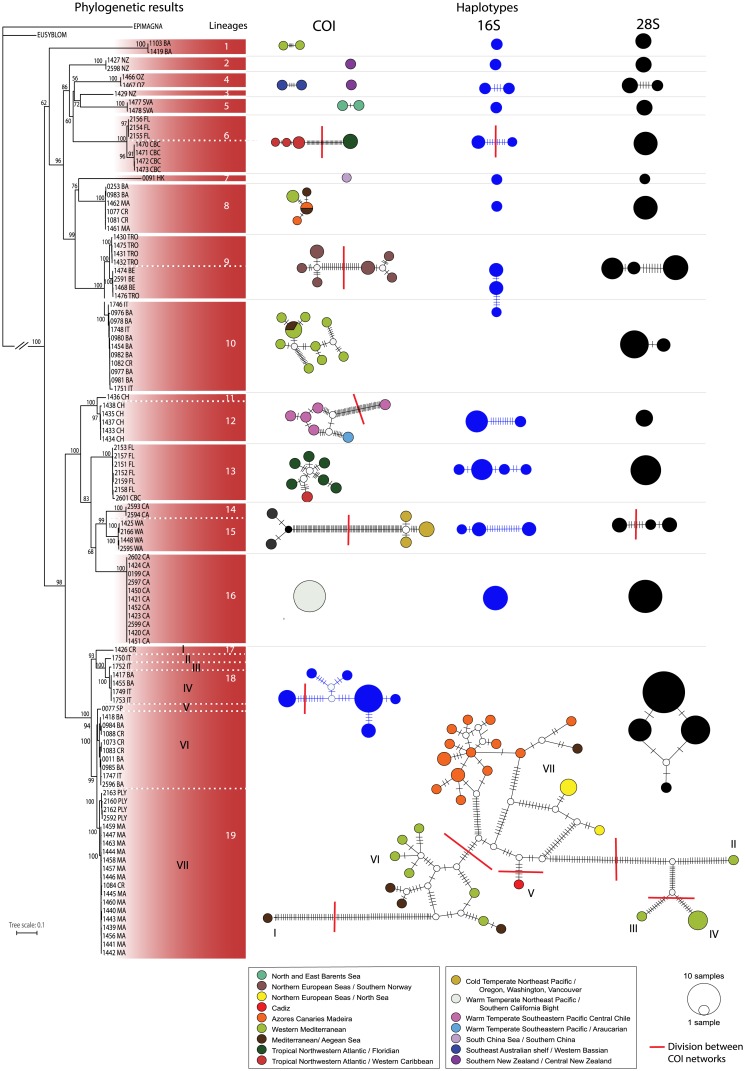
Phylogenetic results and haplotypes. On the left: Maximum Likelihood tree obtained when analysing the combined data set (COI+16S+28S). Bootstrap support values (B) above nodes. Lineages considered are those with support of 100 B and relative length of branch length. On the right: Haplotype networks from markers: COI (colours correspond to bioregions, see legend); 16S (blue) and 28S (black). Size of circles corresponds to sample size (see legend on figure). Each network represents congruent results after TCS analysis of each of the three markers. Red lines indicate different network incongruent results from at least one marker.

**Fig 6:**
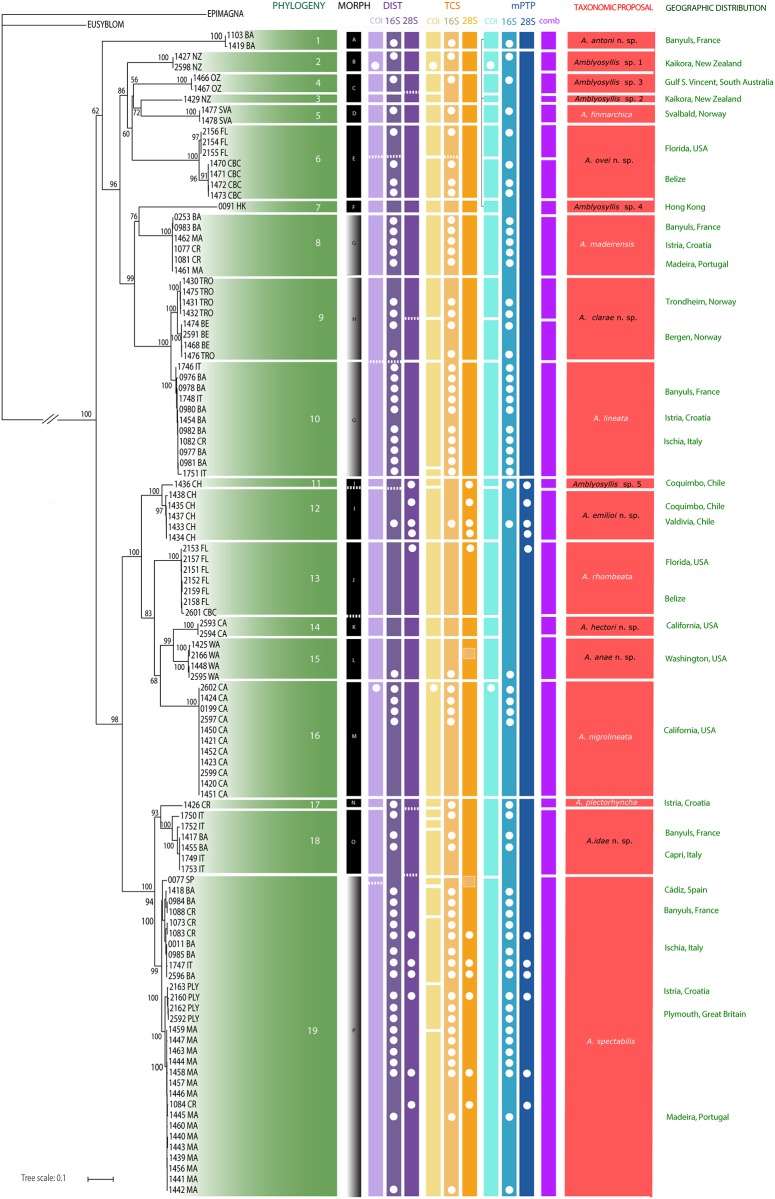
Summary of results obtained through different methodologies. From the left to the right: phylogenetic analysis (ML tree from combined data set (COI+16S+28S)); morphology; distances; TCS; mPTP. On the right: taxonomic proposal and geographic localities of samples. White dots represent missing data. Bars in grey (morphogroups G, H and P) represent variability regarding some of the selected morphological characters to differentiate morphospecies.

Results obtained after analysing the partitions independently and the combined data sets through ML and BI, respectively, show equivalent topologies and support values. The slight differences between results from each independent partition and the combined data sets are explained by the differences in the terminals included [different number of sequences for each marker (see S1 File)]. The ML tree of the combined data set (COI+16S+28S) is shown in Figs 5 and 6. [See in S2 File, the ML tree from the mitochondrial combined data set (COI+16S) and the BI tree from the combined data set (COI+16S+28S)].

The combined analysis (COI+16S+28S) shows *Amblyosyllis* as a well-supported monophyletic group. Within the large clade containing the 109 included terminals (for which we obtained molecular data), there are two large groups (Figs 5 and 6); the upper one is not highly supported. Within them, there are several monophyletic groups or lineages. Lineages supported by 100 B and branches relatively long (>0.03 mutations per site, when considering the sum of the length of the branches separating the nodes) have been considered to have the potential to represent different species. In total, within the studied *Amblyosyllis*, there are 19 lineages considered putative species (named as lineages 1–19 in Figs 5 and 6). Regarding the geographic occurrence, habitat and depth of the samples (S1 File), several lineages have, at least partially, overlapping distribution ranges and ecological preferences: six lineages (1, 8, 10, 17, 18 and 19) are present in the Mediterranean; lineages 8 and 19 also in the East Atlantic (Madeira); lineages 6 and 13 were collected in Florida and Belize; lineages 14 and 16 are from California (Fig 6).

The 19 lineages agree with the identified morphospecies (A–P) (Table 2, Fig 6), except for lineages 3 and 4 (morphospecies C), 8 and 10 (morphospecies G), and 11 and 12 (morphospecies I), that were identified in pairs as the same morphospecies (Fig 6). Monophyly of morphospecies C was not assessed (it was recovered as paraphyletic with low support; Fig 6), and specimens collected in the Svalbard archipelago, that show morphological differences, were found closely related. Morphospecies G was also recovered paraphyletic (Fig 6), forming two clades with members identified as morphospecies H nested within. Mosphospecies I was recovered monophyletic, but one of the sequences branched off at the base of this clade with a branch longer than 0.03 (criterion used herein to split lineages in putative species).

We accept the possible presence of cryptic or pseudocryptic species among the identified morphospecies. Therefore, the 19 lineages were the hypothesis to be tested further with additional methods in order to find the species boundaries.

### Genetic distance and sequence-based species delimitation results

The calculated genetic distances, in COI sequences, between the 19 putative species, based in morphological examination and phylogenetic analyses, were higher than 10% (in most cases p-distance = 18–28% divergent) in all except two cases (p-distance between lineages 9–10 and 11–12), in which they were between 6–8.5%. Intraspecific genetic divergences were amid 0–4% (see S3 File).

The interspecific p-distance measured in 16S sequences were, in most cases, between 4–28%, with two exceptions (i.e. lineages 9–10, 11–12, with p-distances of 1.5%, 2.9%, respectively), which were considered to be comparatively low (marked with a dashed-line in Fig 6). Intraspecific genetic divergence (in several cases not measured due to the low number of sequences available) was lower than 1% except for members of the lineage 6, with 1.9% (see S4 File).

The interspecific genetic distance among 28S sequences was, in most cases larger than 4%, except for lineages 17–18–19, with 1% divergence between them, and lineages 13–14–15, with 2–3%. The intraspecific genetic divergence was null in all but lineage 9, with 1.4% (see S5 File).

Haplotype networks were calculated for 107 COI sequences (excluding outgroups) with 624 positions. Eighty-two haplotypes and 28 networks were found after running TCS analyses (Fig 6). For visualisation of the results, PopART was run using the sequences of each of the 19 putative species from phylogenetic analyses independently (Fig 5). For 16S, TCS analyses were performed for 55 sequences, with 364 positions, resulting in 28 haplotypes and 17 networks. For 28S sequences, 98 sequences with 348 positions were analysed with TCS, recovering 23 haplotypes and 13 networks.

The PTP model (single rate PTP) identified 26 clusters for the 107 COI sequences (see S6 File). The high number of the short branches (with zero or close to zero branch length) across the topology is interpreted by the software as the maximum intraspecific diversity, splitting the terminals into a higher number of clusters than expected. Additional analyses of COI sequences with mPTP (multiple rate PTP) identified 18 clusters, avoiding some of the over-splitting, but making instead a mistake of clustering clades 1 with singletons 3, and 7, that are not sister-clades (see S6 File). The number of clusters identified after mPTP analyses of 16S and 28S sequences is low (four clusters in each case, Fig 6) due to the low number of sequences considered (given that there are a few number of sequences available for 16S and a few number of haplotypes for 28S) and some singletons were grouped together. These latter results contradict the evidence provided by morphology, phylogenetic analyses, and genetic distances, and are not considered as reliable.

In contast, mPTP analyses of combined data identified 21 clusters (see S6 File); congruent with the 19 putative species recovered from previous analyses, except for the lineages 6 and 9 that were split in two additional groups each (Fig 6). In this case, the clades 1, 3 and 7 are identified as independent clusters.

### Summary of results

In this study, we have worked with three different groups of methodologies that were applied sequentially and iteratively. Firstly, morphology was examined and rendered a hypothesis of 16 morphospecies. Secondly, phylogenetic analyses were performed, recovering 19 different lineages with relatively long branches and high support, therefore indicating putative species boundaries (considering species as independently evolving entities that are genetically distinct). Genetic distances (p-distance and model based) were calculated between and within the 19 lineages (for each of the three markers COI, 16S and 28S). Finally, species delimitation methods, including statistical parsimony haplotype networks were also performed and the ML trees were used as the input for PTP and mPTP.

The combination of all results can be seen in Fig 6 and are herein summarized in more detail:

- Phylogenetic analyses provide evidence for monophyly of *Amblyosyllis*. Preliminary morphospecies are congruent with most clades within the genus (Figs 5 and 6). There are some exceptions: morphospecies C is paraphyletic and G corresponds to two different and not closely related lineages each (Fig 6). Morphospecies I is split into two sister clades with significant genetic divergence (nuclear and mitochondrial markers) regardless sympatric distribution.
- The genetic diversity in the genus is high, contrary to the morphological homogeneity observed. The corrected pairwise p-distance among members of *Amblyosyllis* is mostly up to 23.2%, 25.4% and 28.5% in COI, 16S and 28S, respectively (see S3–S5 Files).
- Statistical parsimony analyses find 28 COI haplotype networks.
- This particular dataset, with species showing a relatively low population structure (which may be a biased result since the number of populations sampled is low for some species) and several singletons, is problematic for PTP and mPTP. However, when using the concatenated (COI+16S+28S) ML tree, the program recovers 21 clusters congruent with the 19 lineages, except for lineages 6 (morphospecies E) and 9 (morphospecies H), which are each subdivided in two.
- All the applied methods widely agree in supporting, at least, 19 putative species. This could be considered as a preliminary and conservative measure awaiting new information that may allow us to assess additional species boundaries between members within the lineages 6 and 9 respectively.

### Species delimitation proposal

We have considered five different kinds of results obtained from different methodologies: morphology, phylogeny, distances, TCS and mPTP (Fig 6). Our criteria for making a proposal have been that most of the methods should agree to consider interspecific limits, though each situation has been evaluated independently. In most of the cases all methods agree delimiting species, except for lineages 6, 9 and 19 where some results indicate a possible extra splitting. We have preferred to be conservative and not to oversplit until further analyses, including more material from the given lineages, are performed.

Our results provide us with enough evidence to discriminate between 19 species that have been properly identified (those that were previously described), or described herein by the first time (those that are new to science) (Fig 6). We propose new species names only for those with more than two specimens; otherwise, they are described and named as *Amblyosyllis* sp., except for *A*. *antoni* n. sp. and *A*. *hectori* n. sp., which show clear and consistent morphological diagnostic features. Our proposal (Fig 6) includes four previously described species: *A*. *rhombeata*, *A*. *spectabilis* (former *A*. *formosa*), *A*. *finmarchica*, and *A*. *madeirensis*; three previously synonymised species: *A*. *lineata*, *A*. *plectorhyncha*, and *A*. *nigrolineata*; seven new species: *A*. *antoni* n. sp., *A*. *ovei* n. sp., *A*. *clarae* n. sp., *A*. *emilioi* n. sp., *A*. *anae* n. sp., *A*. *hectori* n. sp. and *A*. *idae* n. sp.; and five possible new species that are left unnamed until more material is available: *Amblyosyllis* sp. 1–5. All of them are described and appropriate iconography is provided (see section Taxonomic Account).

### Nomenclatural Acts

The electronic edition of this article conforms to the requirements of the amended International Code of Zoological Nomenclature, and hence the new names contained herein are available under that Code from the electronic edition of this article. This published work and the nomenclatural acts it contains have been registered in ZooBank, the online registration system for the ICZN. The ZooBank LSIDs (Life Science Identifiers) can be resolved and the associated information viewed through any standard web browser by appending the LSID to the prefix “http://zoobank.org/”. The LSID for this publication is: urn:lsid:zoobank.org:pub:BB05763A-0FC6-46FD-99A4-DC22414916A9. The electronic edition of this work was published in PLOS ONE, a journal with an ISSN (eISSN-1932-6203), and has been archived and is available from PubMed Central and the LOCKSS digital repositories.

The new species LSIDs are:

*Amblyosyllis anae* urn:lsid:zoobank.org:act:B9A0FADE-A625-4B15-8501-2DBBFA3E1195

*Amblyosyllis antoni* urn:lsid:zoobank.org:act:A567179B-11D1-4853-8848-4E30BB16303B

*Amblyosyllis clarae* urn:lsid:zoobank.org:act:4278DCE5-2479-4236-8314-BB155F0E8DD5

*Amblyosyllis emilioi* urn:lsid:zoobank.org:act:B4305B0B-0DED-4636-961F-AEE3E9F898FD

*Amblyosyllis hectori* urn:lsid:zoobank.org:act:5E446EC3-9ADB-4BB0-AB7D-33E7F97CC5B5

*Amblyosyllis idae* urn:lsid:zoobank.org:act:0D3F5304-6DAB-4DCC-B32A-30A69D2CC4B8

*Amblyosyllis ovei* urn:lsid:zoobank.org:act:7D7159D4-B29B-4480-BE1A-E4F6E18EC56A

### Taxonomic account

Genus *Amblyosyllis* Grube & Ørsted *in* Grube, 1857

*Amblyosyllis* Grube & Ørsted *in* Grube, 1857 [2]: 186.

*Gattiola* Johnston *in* Baird, 1861 [80]: 298.

*Pterosyllis* Claparède, 1863 [4]: 46.

*Nicotia* Costa, 1864 [6]: 160–164.

*Thylaciphorus* Quatrefages, 1865 [108]: 55.

*Pseudosyllides* Czerniavsky, 1882 [109]: 173.

**Diagnosis.** Body dorso-ventrally flattened, with 15 segments (peristomium or first segment, 13 chaetigers and one achaetous prepygidial segment). Segments trapezoidal in shape. Prostomium rounded, with four large red eyes in trapezoidal arrangement, and usually two anterior eyespots, sometimes ventrally located. Palps shorter than length of prostomium, basally fused and divergent, usually ventrally folded. Peristomium or first segment shorter than following ones, with two pairs of tentacular cirri, and two nuchal external organs or nuchal lappets, usually ciliated, occasionally absent. Antennae, tentacular and dorsal cirri long, smooth to distally articulated, often coiled over the dorsum. Ventral cirri large, lanceolate or digitiform, located latero-posteriorly to parapodial lobes, usually not exceeding parapodial length. Dorsal and ventral cirri with glands, those within dorsal cirri organized in longitudinal lines. Parapodial lobes long, with conical prechaetal lobes, distally pointed and with a distinct dorsal prechaetal digitiform papilla. Midbody parapodia with 4–5 pointed aciculae. Compound chaetae heterogomph, bidentate falcigers, occasionally unidentate or obscurely bidentate, with fine spinulation on verge. Cylindrical pre-pygidial segment without parapodia or chaetae. Pygidium with two pairs of anal cirri, similar in length and shape to dorsal ones. Pharynx long, slender, highly convoluted, with trepan formed of several teeth, generally six or eight, lacking median tooth; trepan might be absent. Proventricle barrel shaped, relatively short, occupying one of two segments. Reproduction by epigamy, one species is known to brood eggs in a gelatinous mass.

**Type species:** *Amblyosyllis rhombeata* Grube & Ørsted *in* Grube, 1857, by monotypy.

**Type locality:** Saint Croix, Virgin Islands, West Atlantic.

**Remarks:** The intricate taxonomy of the genus *Amblyosyllis* is the result of several different factors co-occurring together: i) the traditional difficulty in finding good and reliable taxonomic characters on which to base the descriptions; ii) the fact that the conspicuous colour patterns used in the recognition of many species not only are lost upon preservation but also show, in some cases, intraspecific variability; iii) the extreme frailty of the specimens; and finally iv) the complex taxonomic history of the group, with numerous early names having to be considered, as well as many unsolved synonymies, most of which due to lack of type material or ancient descriptions being inadequate in face of present day standards.

The complex taxonomic history of the genus includes six different generic names in 25 years, from 1857 to 1882, 15 of 22 species and subspecies being described before the end of the 19th century, and only four after 1960, two of which in the 21st century.

The name *Amblyosyllis* was the first to be introduced [2], for the species *Amblyosyllis rhombeata*. The taxa were based on material observed and collected by Ørsted at Saint Croix, in the Caribbean Sea (by then a Danish possession, part of the Danish West Indies), and also on Ørsted’s own notes and sketches from the living specimens [31, 74]. The shared authorship of both genus and species was clearly expressed in the publication as “Gr. Örsd.”, but it has been normally attributed to Grube alone (e.g. [3, 18, 30]), in spite the fact that Grube himself cited the authorship as being shared (e.g. [2]). Salazar-Vallejo & Eibye-Jacobsen [110] revised the problem and supported the authorship as being “Grube & Ørsted *in* Grube”, following Recommendation 51E of the ICZN [111], a shared authorship that will be also followed here. However, the same authors stated the publication date of the taxa as “1858”, which is incorrect. The volume containing the paper with the descriptions has a footnote on page iii, under “Indhold” (= “Contents”), stating “De særskilte Aftryk af Afhandlingerne indtil Side 199 vare omdelte til Forfatterne inden October 1857. Det samlede Hefte færdigt til Omdeling 26de Februar 1858” (= “The separate reprints of the dissertation until page 199 were distributed to the authors by October 1857. The total volume was completed on 26 February 1858”). The pagination of the paper is 158–186, implying that it was already available in 1857, before the publication of the full volume in the following year. Consequently, we consider the authorship and publication date of both genus *Amblyosyllis*, and species *A*. *rhombeata*, as being “Grube & Ørsted *in* Grube, 1857”.

The following name to be published associated with the group was *Cirrosyllis* [106], often considered to be “in part” a synonym of *Amblyosyllis* (e.g. [30]). The genus was established in Hesionidae, to include six new species: *Cirrosyllis ceylandica* Schmarda, 1861 (from Sri Lanka), *C*. *didymocera* Schmarda, 1861 (Port Jackson, Australia), *C*. *incerta* Schmarda, 1861 (New Zealand), *C*. *picta* Schmarda, 1861 (Cape of Good Hope), *C*. *tuberculata* Schmarda, 1861 (Cape of Good Hope), and *C*. *vittata* Schmarda, 1861 (Croatia, Adriatic Sea) [106]. Schmarda did not designate a type species, and shortly after the genus was considered to be polyphyletic (e.g. [48, 108, 112]). With most of the types been lost, its species have been scattered through different genera belonging to families Hesionidae, Syllidae, Dorvilleidae, and Nereididae, or stated to be indeterminable [30, 48, 108, 109, 113–120]. Yet, Grube [121] did designate *Cirrosyllis didymocera* as the type species of the genus, a subsequent designation that has been overlooked since then. *Cirrosyllis didymocera* was combined in the genus *Oxydromus* Grube, 1855 [72] by Villalobos-Guerrero & Harris [122], as *O*. *didymocerus* (Schmarda, 1861) [106], turning the genus *Cirrosyllis* into a junior synonym of *Oxydromus* and removing it from the list of synonymies of *Amblyosyllis*.

In relation with *Cirrosyllis*, Czerniavsky [109] created the new genus *Pseudosyllides* Czerniavsky, 1882 [109] in Hesionidae to include Schmarda’s *Cirrosyllis picta*. Its holotype was later revised by Augener [48] and determined to be a valid *Amblyosyllis*, as *A*. *picta*, rendering this way *Pseudosyllides* Czerniavsky, 1882 [109] a junior synonym of *Amblyosyllis*.

Particularly relevant is the case of *Gattiola* Johnston *in* Baird, 1861 [80], with the information found in the present work changing priorities in the group and having implications in the accepted synonymies. *Gattiola* was originally created by George Johnston in his “Catalogue of British non-parasitical worms in the collection of the British Museum”, to include *Gattiola spectabilis*, from British waters. Johnston died in 1855 while his “Catalogue” was still in press, remaining unpublished in the hands of his colleagues J.E. Gray and W. Baird, who would manage its posthumous publication only in 1865 [1]. However, the names “*Gattiola* Johnston / *spectabilis* Johnston” appeared published a first time as *nomina nuda* in 1860 in a checklist of the British marine invertebrate fauna [123], where Johnston’s unpublished list of Annelida was used with the permission of J.E. Gray.

Yet, both names appeared formally published shortly after in the Annelida section of “The Museum of Natural History” [124], in a footnote by Baird [80]. The exact publication date is subject to dispute, as the two volumes of the work were published undated, but Curvey & Johnson [125] considered 1859 for volume I, and 1862 for volume II, including the Annelida section. Nevertheless, some book antiquarians also refer an original and different binding of the same publication in eight thinner volumes, suggesting that the different sections of the work were published in sequential instalments, on different dates, and binded together later.

Wright [126] registered both genus and species in the Zoological Record as attributed to Johnston and being published in 1861: “This genus (*Gattiola*) was published in 1861 by Dr. Baird, in article “Annelida” in ‘Museum of Natural History’, vol. ii. p. 298 (note)”. 1861 is also contemporary referred by Baird himself [127] in the “Addenda and Corrigenda” to Johnston’s posthumous “Catalogue”, Malmgren [33], who stated the authorship as being “Johnston”, or Langerhans [29], who attributed the taxa to “Baird”. However, most authors (e.g. [7, 18, 30, 32, 88, 128]), overlooked the 1861’s note, erroneously accepting “Johnston, 1865”, when they should be “Johnston *in* Baird, 1861”.

This has no consequences in what concerns the generic priorities, as the priority of *Amblyosyllis* is clear in relation to *Gattiola*, but it does affect the specific priorities and synonymies, with *Gattiola spectabilis* Johnston *in* Baird, 1861 [80] having priority over *Pterosyllis formosa* Claparède, 1863 [4], *Pterosyllis dorsigera* Claparède, 1864 [5], and even *Thylaciphorus hessii* Quatrefages, 1865 [108].

**Etymological considerations:** *Amblyosyllis* is now recognised as a distinct and well defined group, but during the 19th century it received several different names, resulting in a total of five synonymies (see above). The etymology of *Amblyosyllis*, as well as of its synonymies, were seldom referred in the original descriptions. Therefore, we suggest here the following reasons that may have motivated the original authors to choose those names:

*Amblyosyllis* Grube & Ørsted *in* Grube, 1857 [2]: etymology not stated in the original description. The name is composed by the combining form of Greek origin *ambly-* or *amblyo-*, meaning ‘blunt’, ‘dim’, or ‘dull’, followed by the name of the genus *Syllis* Savigny *in* Lamarck, 1818 [129], type genus of the family. The name could refer to the lack of bright colors of the type species, described has being white with black streaks, or to the shape of the body, with the lateral margin being coarsely serrated, not sharply pointed. The prefix *Amblyo*- can be currently found in use in some medical terms, for instance *Amblyopia* or “lazy eye”, where it means ‘vage’. In *Amblyosyllis* it may also refer to the slow movements of these animals, in comparison with other syllids.

*Gattiola* Johnston *in* Baird, 1861 [80]: according to Baird [80], the genus was named by G. Johnston “in compliment to his friend Mrs. (Margaret S.) Gatty, authoress of the well-known “Parables from Nature” (a children’s book).” Margaret Gatty was also an enthusiast amateur marine biologist.

*Pterosyllis* Claparède, 1863 [4]: etymology not stated in the original description. The name of the genus is formed by the Ancient Greek noun *ptero*, meaning ‘wing’ or ‘feather’, followed by the name of the genus *Syllis*. The name refers to the two external nuchal lappets, described in the generic diagnosis as “flügelartige” (= “wing-like”), resembling small “wings” arising from the peristomium. According to Claparède [4] these peculiar structures justify the formation of a new genus, being otherwise similar to the genus *Syllis*. Langerhans [29] refers to these structures in his diagnosis of the genus as “capite alato” (“winged head”).

*Nicotia* Costa, 1864 [6]: etymology not stated in the original description, but its type species, *N*. *lineolata*, is described by Costa [6] has having the posterior segments of the body “di color tabacchino chiaro”, which could be translated roughly as “light tobacco colored”. *Nicotia* seems to refer to the tobacco plant, *Nicotiana*, named after Jean Nicot, a French diplomat responsible for the introduction of snuff tobacco in the French royal court in the 16th century, after visiting Lisbon as an ambassador to negotiate the marriage between the Princess Margaret of Valois with the King Sebastian of Portugal.

*Thylaciphorus* Quatrefages, 1865 [108]: etymology not stated in the original description. The name is composed by the prefix *thylacis*, from the Ancient Greek *thúlakos*, meaning ‘pouch’ or ‘sack’, and referring probably to the carnivorous marsupial of the genus *Thylacinus*, described in 1824 by Temminck and commonly known as Tasmanian wolf or tiger, followed by the suffix -*phorus*, from the Ancient Greek *phóros* and meaning ‘bearer’ or ‘carrier’. It refers presumably to the resemblance between the black dorsal streaks borne by the type species of the genus, *T*. *hessii* Quatrefages, 1865 [108], and the black streaks typical of the Tasmanian tiger.

*Pseudosyllides* Czerniavsky, 1882 [109]: etymology not stated in the original description. The name is formed by the prefix *pseudo*-, meaning ‘similar to’ (from the Greek *pseudes*, meaning ‘lying’ or ‘false’), followed by the name of the genus *Syllides* Ørsted, 1845 [130], and refers probably to the resemblance between the two genera.

#### Species descriptions

For the identifications of species already described, i.e. *A*. *rhombeata*, *A*. *spectabilis* (former *A*. *formosa*), *A*. *madeirensis*, and *A*. *finmarchica*, and those whose names were previously synonymised with other species and are herein reinstated, i.e. *A*. *lineata*, *A*. *plectorhyncha* and *A*. *nigrolineata* (Table 1), we have mostly considered only the original description and the type locality. Especially, *A*. *spectabilis* (as *A*. *formosa*) and *A*. *madeirensis* have been cited numerously, in many occasions without a detailed description, and their distribution has been considerably expanded. It is difficult to know which of these reports correspond with what we herein propose as *A*. *spectabilis* and *A*. *madeirensis* without the backup of molecular information. In any case, at least some of our samples come from the type locality or close geographical areas. The type series of some species have been revised and compared with the present material, but unfortunately many others never existed (e.g. *A*. *formosa*, *A*. *dorsigera*), were lost (e.g. *A*. *rhombeata*), or so far were not located (e.g. *A*. *spectabilis*, *A*. *madeirensis*, *A*. *nigrolineata*). When available, COI sequences of the specimens studied herein have been compared with data from GenBank (sequences only available for *A*. *madeirensis*, *A*. *finmarchica*, *A*. *nigrolineata* and *Amblyosyllis* sp.) (see corresponding remarks of the species). When the GenBank sequences and ours were more than 97% similar, we expanded the distribution of the species to the areas where those sequenced specimens were collected.

The invariable characters (Fig 3) included in the diagnosis of the genus are not repeated in the species descriptions. The body length measurement is given for the preserved material, and hence, in most of cases, posterior end is missing (used for DNA extraction).

The species descriptions are organized following the order of lineages (Figs 5 and 6), beginning by lineage 1 and finishing by lineage 19.

Detailed information concerning the localities where the present material was collected can be found in S1 File.

#### Lineage 1

***Amblyosyllis antoni* n. sp.**

Figs 7 and 8

**Fig 7:**
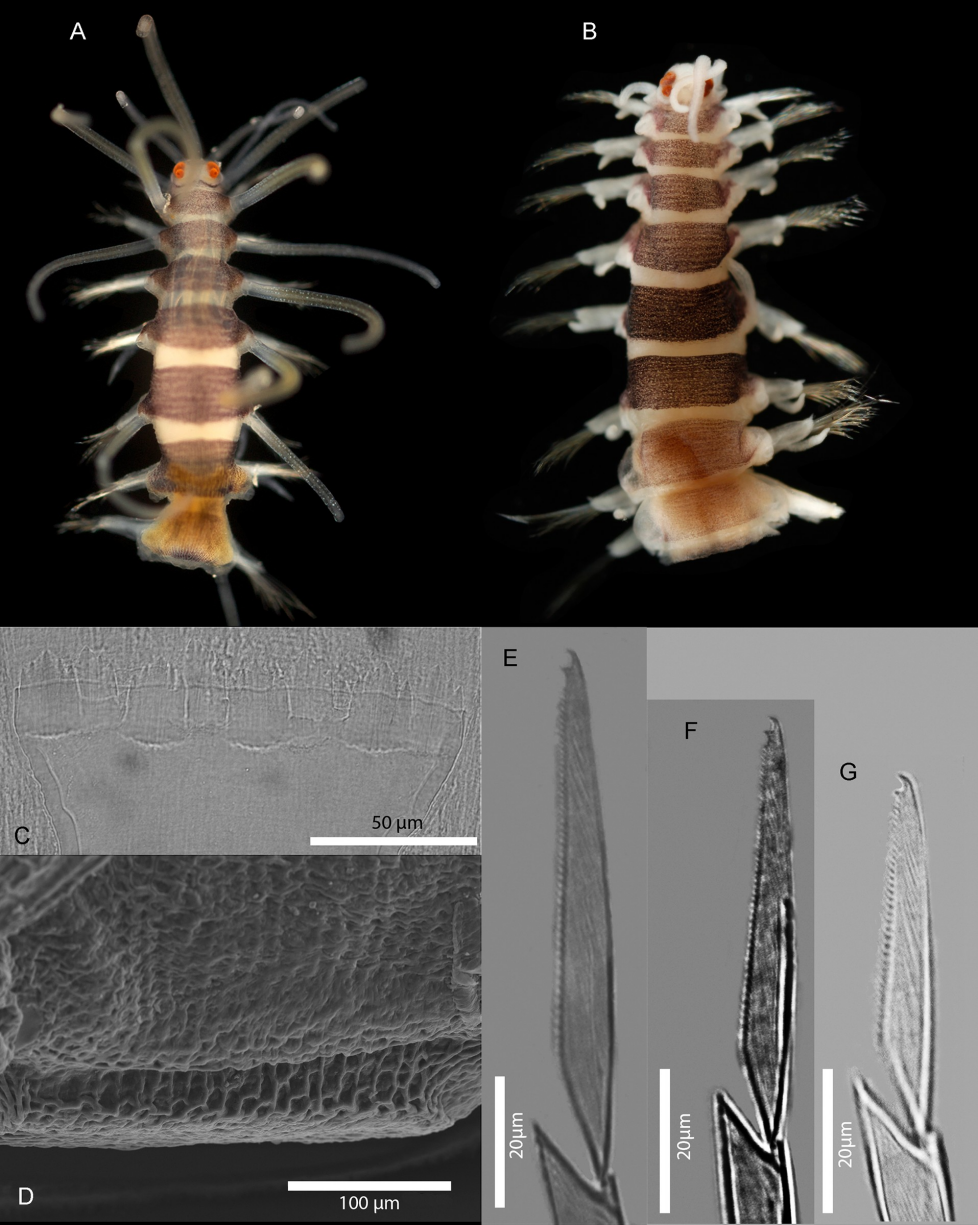
*Amblyosyllis antoni* n. sp. (A) Anterior end, live specimen, dorsal view, MNCN 16.01/18463. (B) Anterior end, preserved specimen, dorsal view, MNCN 16.01/17972. (C) Trepan. (D) Detail of the proventricle, SEM. (E) Dorsal chaeta, midbody segment. (F) Medial chaeta, midbody segment. (G) Ventral chaeta, midbody segment.

**Fig 8:**
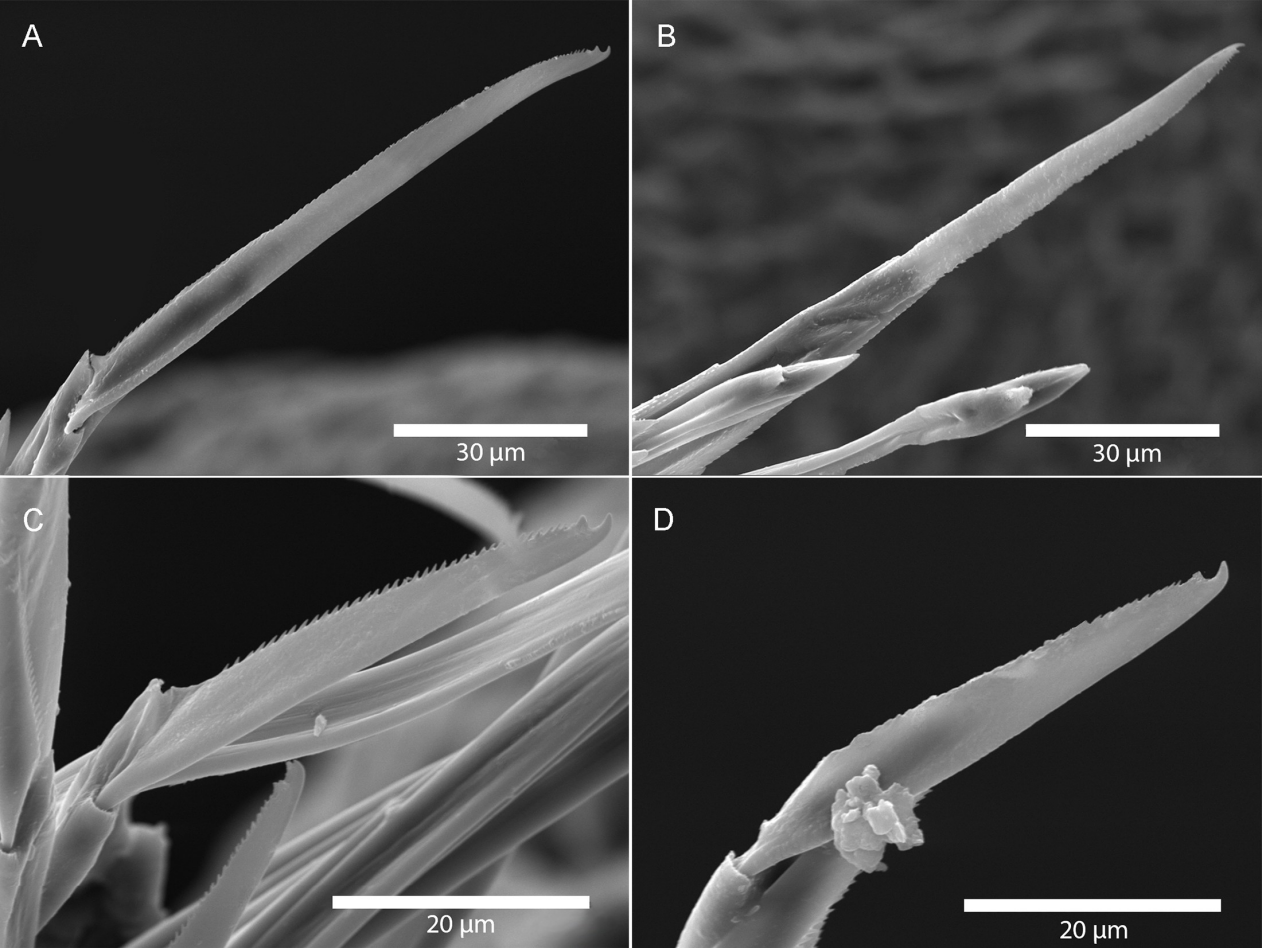
*Amblyosyllis antoni* n. sp. SEM. (A) Dorsal chaeta, anterior segment. (B) Dorsal chaeta, anterior segment. (C) Medial chaeta, anterior segment (D) Ventral chaetae, anterior segment.

**Material examined:** Holotype (MNCN 16.01/17972), and paratype (MNCN 16.01/18463).

**Type locality:** Banyuls-sur-Mer, France (Mediterranean Sea).

**Habitat:** 25 m depth, on calcareous algae.

**Distribution:** Only known from type locality.

**Description:** Holotype incomplete, with eight segments, 2.95 mm long, 0.8 mm wide. Segments dorsally subannulated. Strong dark colouration as several wide transversal bands per segment (Fig 7A and 7B). Pigmentation intensity decreases posterior to proventricle. Nuchal lappets absent or very reduced as ciliary arches postero-laterally on prostomium (Fig 7A and 7B). Long dorsal cirri with longitudinal rows of glands visible by transparency, ventral cirri longer than parapodia, distally pointed (Fig 7B). Parapodia with several compound chaetae, up to 15–18 on midbody, with long, distinctly bidentate blades (Figs 7E–7G and 8A–8D); distal and proximal teeth approximately equivalent in size (Figs 7E–7G, 8A and 8C). Trepan with eight pentacuspid teeth (Fig 7C). Proventricle with muscular cells quadrangular in shape (Fig 7D).

**Remarks**: The absence or strong reduction of nuchal lappets and a trepan composed of eight teeth are distinctive features that separates *A*. *antoni* n. sp. from any other congeners described previously. It is similar to members of *Brachysyllis* in general body shape, in the absence of nuchal lappets and in the shape of anterior segments, which are not distinctly trapezoidal [13]. However, this species shows a trepan with pentacuspid teeth (they are conical in *Brachysyllis*), a convoluted pharynx (straight in *Brachysyllis*), trapezoidal segments after the proventricle and absence of a middorsal pharyngeal tooth (diagnostic of *Brachysyllis*).

**Etymology:** This species is dedicated to Arne Nygren’s son, Anton Nygren.

#### Lineage 2

***Amblyosyllis* sp. 1**

Fig 9

**Fig 9:**
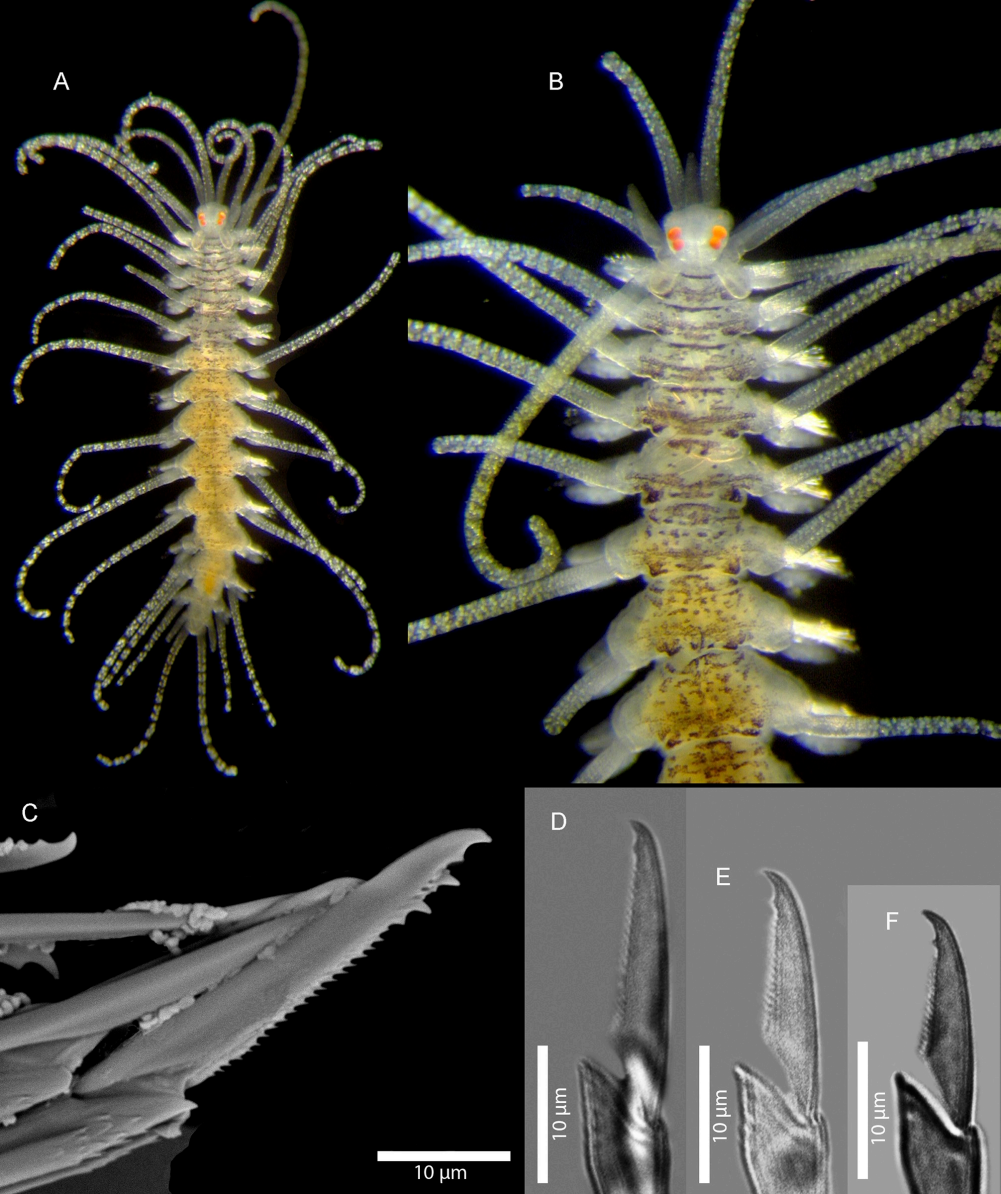
*Amblyosyllis* sp. 1. (A) Live specimen, dorsal view, MNCN 16.01/17973. (B) Live specimen, anterior end, dorsal view, MNCN 16.01/17974. (C) Anterior chaetae, SEM. (D) Dorsal chaeta, midbody segment. (E) Medial chaeta, midbody segment. (F) Ventral chaeta, midbody segment.

**Material examined:** Two specimens (MNCN 16.01/17973–4).

**Locality of material examined:** Kaikora, New Zealand (South Pacific Ocean).

**Habitat:** Intertidal; rock pools with algae.

**Description:** Best preserved specimen (MNCN 16.01/17973) incomplete after fixation, with seven chaetigers, 1.3 mm long, and 0.63 mm wide. With several transversal lines and spots per segment (Fig 9A and 9B), colour pattern less distinct in more posterior segments. Nuchal lappets long (Fig 9A and 9B), extending over half of the second segment, ciliated and with some colouration. Long dorsal cirri visibly articulated. Parapodia with distinct prechaetal digitiform papilla; ventral cirri no longer than parapodial lobes, lanceolate, with rounded glands inside. Several compound chaetae, up to 16–18 on midbody parapodia, with short, distinctly bidentate blades (Fig 9C–9F). Distal tooth considerably larger than proximal one (Fig 9D–9F). Pharynx not completely everted in both preserved specimens. Trepan not observed.

**Remarks:** The chaetae, although shorter in length and with less dorso-ventral gradation, show some resemblance with those from *A*. *enigmatica* San Martín & Hutchings, 2006 [19] from Australia, which differs in showing an additional appendage [10, 19]. One specimen from Lizard Island (Australia) identified as *A*. *enigmatica* [10] was sequenced (COI) and compared with the present specimen, sharing the same nucleotides on 81% of sites, which is the reason why we consider *Amblyosyllis* sp. 1 to represent a different species. The banner of World Polychaeta Database [131] shows one *Amblyosyllis* from New Zealand whose colour pattern resembles *Amblyosyllis* sp. 1.

#### Lineage 3

***Amblyosyllis* sp. 2**

Fig 10

**Fig 10:**
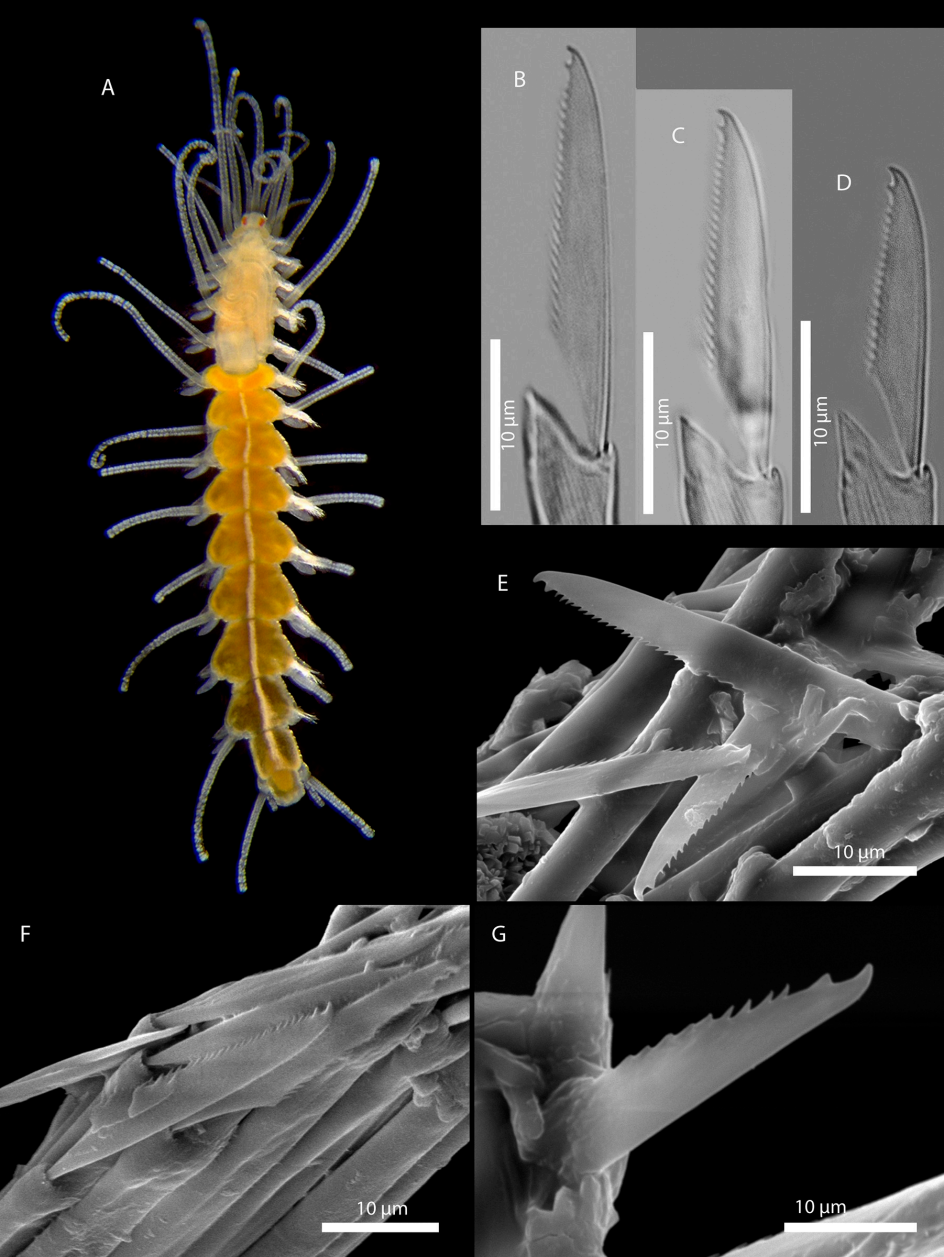
*Amblyosyllis* sp. 2. (A) Live specimen, dorsal view, MNCN 16.01/17975. (B) Dorsal chaeta, midbody segment. (C) Medial chaeta, midbody segment. (D) Ventral chaeta, midbody segment. (E) Dorsal chaeta, anterior segment, SEM. (F) Medial chaeta, anterior segment, SEM. (G) Ventral chaeta, anterior segment, SEM.

**Material examined:** One specimen (MNCN 16.01/17975).

**Locality of material examined:** Kaikora, New Zealand (South Pacific Ocean).

**Habitat:** Intertidal; rock pools with algae.

**Description:** Preserved specimen incomplete, 10 segments, 2.25 mm long, 0.7 mm wide. Pale whitish ground colour in the first segments, turning into brown-yellowish posterior to the proventricle and accompanied by one longitudinal white mid-dorsal band towards the end of the body, not visible in preserved specimens (Fig 10A). Nuchal lappets long (Fig 10A), reaching up to the 2nd chaetiger, with brownish colouration. Bunches of cilia over the dorsum. Large ventral cirri slightly longer than parapodial lobes, spindle shaped. Parapodia with up to 14–16 compound chaetae on midbody, with short, distinctly bidentate blades (Fig 10B–10G). Distal and proximal teeth approximately equivalent in size (Fig 10B–10G). Pharynx everted, trepan not observed.

**Remarks:** The same colour pattern and similar chaetae are found in *Amblyosyllis* sp. 3. However, they do differ in chaetal length, having shorter chaetae and dorso-ventral gradation not as evident as in *Amblyosyllis* sp. 3. The available material consists of only one specimen that probably merits to be described as new species when more material becomes available. *Amblyosyllis enigmatica*, a species reported from Australia, shows different colour pattern and chaetal shape. Additionally, the COI sequence of one specimen of *A*. *enigmatica* [10] was available; we aligned and compared the sequences, obtaining a similarity of 82%, which confirms that they represent different species.

#### Lineage 4

***Amblyosyllis* sp. 3**

Fig 11

**Fig 11:**
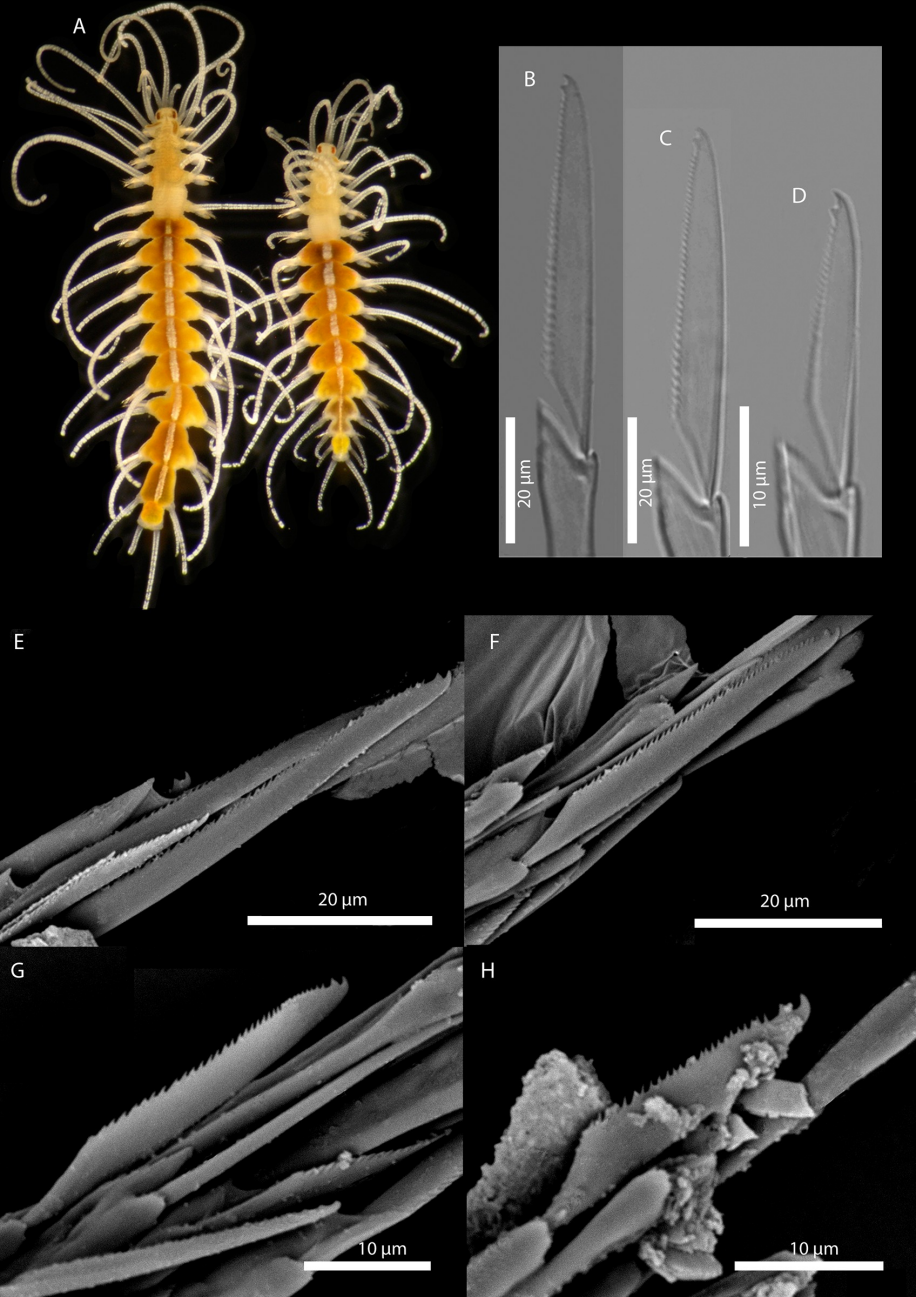
*Amblyosyllis* sp. 3. (A) Live specimens, dorsal view, MNCN 16.01/17976. (B) Dorsal chaeta, midbody segment. (C) Medial chaeta, midbody segment. (D) Ventral chaeta, midbody segment. (E) Dorsal chaeta, anterior segment, SEM. (F) Medial chaeta, anterior segment, SEM. (G) Medial chaeta, anterior segment, SEM. (H) Ventral chaeta, anterior segment, SEM.

**Material examined:** Two specimens (MNCN 16.01/17976–7).

**Locality of material examined:** Yorke Peninsula, Cowbowie Field Station, Gulf St. Vincent, South Australia (Indian Ocean).

**Habitat:** Between 3–5 m; mixed sand and gravel.

**Description:** Best preserved specimen (MNCN 16.01/17976) incomplete, with 13 segments, 3 mm long, 0.68 mm wide. With pale whitish ground colour in the first five chaetigers, turning into brown-yellowish tone in the rest of the body; posterior to the proventricle one longitudinal middorsal white band towards the end of the body, not visible in preserved specimens (Fig 11A). Dorsal surface covered by bunches of cilia, clearly visible under optical microscope in both sides of segments. Nuchal lappets long, extending over half of the second chaetiger, presenting dark dorsal pigmentation (Fig 11A). Dorsal cirri distally articulated, distinct prechaetal digitiform papilla; ventral cirri as long as parapodial lobes, spindle shaped, with granular material and some dark colouration at the tips. Parapodia with several medium length compound chaetae, around 17–20 on midbody parapodia, with bidentate blades (Fig 11B–11H). Trepan not observed.

**Remarks:** As for *Amblyosyllis* sp. 2, the colour pattern and chaetae shape differ from those of *A*. *enigmatica*. The COI sequences of *A*. *enigmatica* [10] and *Amblysyllis* sp. 3 were aligned and compared, obtaining a similarity of 81%, which confirms they belong to different species.

#### Lineage 5

***Amblyosyllis finmarchica* (Malmgren**, **1867)**

Fig 12

**Fig 12:**
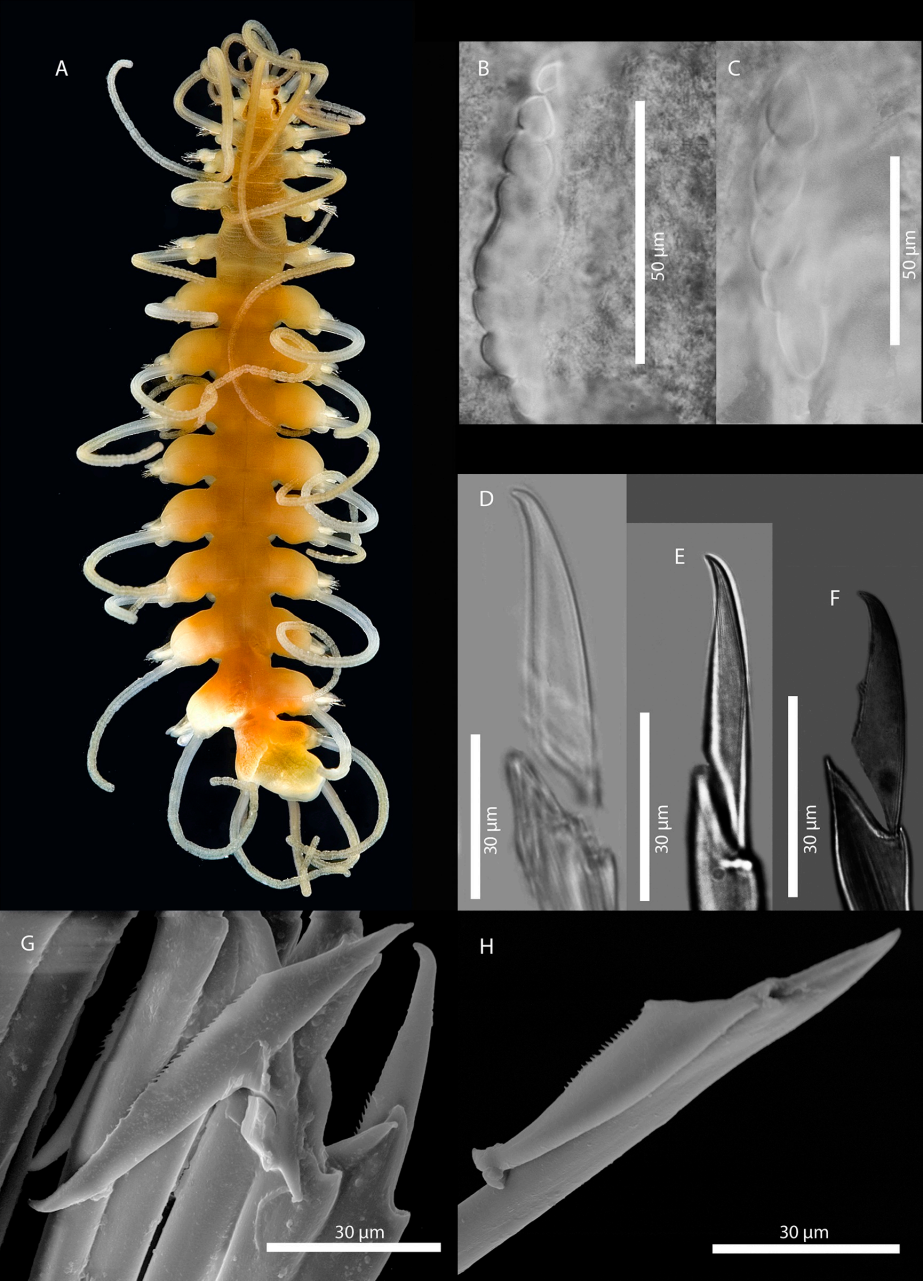
*Amblyosyllis finmarchica*. (A) Live specimen, dorsal view, MNCN 16.01/17978. (B) Trepan tooth (nine cusps in focus). (C) trepan tooth (five cusps in focus). (D) Dorsal chaeta, midbody segment. (E) Medial chaeta, midbody segment. (F) Ventral chaeta, midbody segment. (G) Medial chaeta, anterior segment, SEM. (H) Ventral chaeta, anterior segment, SEM.

*Gattiola finmarchica* Malmgren, 1867 [33]: 38–39, plate VI, Fig 36.

? *Gattiola cincinnata* Verrill, 1874 [37]: 391, plate 2, Fig 1.

**Type locality:** Finnmark, northern Norway (Norwegian Sea).

**Material examined:** Type-2442 SMNH; two specimens (MNCN 16.01/17978–9).

**Locality of material examined:** Hinlopen, Svalbard, Norway (Arctic Ocean).

**Habitat:** 52 m; stones and gravel.

**Distribution:** Norway, Russia, and Canada.

**Description of material examined:** Best preserved specimen (MNCN 16.01/17978) with 10 chaetigers, 4 mm long and 1 mm wide. With transversal brownish lines in the first five chaetigers; pigmentation lighter posterior to the proventricle (Fig 12A). Nuchal lappets long, extending over half of the second chaetiger, presenting dark dorsal pigmentation (Fig 12A). Prechaetal papillae triangular in shape. Ventral cirri rounded, as long as parapodial lobes. Around 30 compound chaetae in midbody parapodia, with unidentate blades, or with a minute proximal tooth (Fig 12D–12H). Chaetae medium to short, with slight dorsoventral gradation in length. Pharynx partially everted in preserved material. Trepan with six teeth with several cusps (multicuspid), five to nine cusps well seen in some teeth (Fig 12B and 12C). Both specimens are epigamic, segments full of gametes after the proventricle, massive.

**Remarks**: The morphology of the examined material matches with the type. This is a species easy to identify due to the presence of unidentate chaetae (or with a minute proximal tooth) and size, which seems to be larger than in other species. Helgason *et al*. [36] include a drawing of one trepan tooth of *A*. *finmarchica* with multiple cusps (11, nine larger), which agrees with our specimens. The only other described species with unidentate or obscurely bidentate chaetae is *A*. *cincinnata*, from the North West Atlantic Ocean [22, 37] (Table 1), whose trepan shows 11 cusps according to Riser [22]. This species should probably be synonymised with *A*. *finmarchica*, but we prefer to wait until more material from the type locality (Maine, USA) is available to be sequenced and compared.

The COI sequences of our specimens match 99–100% with those of *A*. *finmarchica* (GU672522, GU672523, GU672591) from Kandalaksha Bay, Russia [51], and 99% with *A*. *finmarchica* (HQ023430) from Manitoba, Canada [52]. This supports the presence of the species at both sides of the northernmost Atlantic Oceans, and the Arctic Ocean, and further supports to the junior synonymy of *A*. *cincinnata*.

*Amblyosyllis finmarchica* was considered to be a *nomen dubium* [132], but it was later [36, 133] recognised as valid based on material from Iceland and Norway, respectively. The present work confirms the validity of the species.

**Etymology:** The specific epithet of *Amblyosyllis finmarchica* refers to Finnmark (Norway), type locality of the species.

*Gattiola cincinnata* was named with a Latin adjective meaning ‘having curly hair’ or ‘having ringlets’, and refers probably to the coiled antennae and cirri of the species [37].

#### Lineage 6

***Amblyosyllis ovei* n. sp.**

Figs 13 and 14

**Fig 13:**
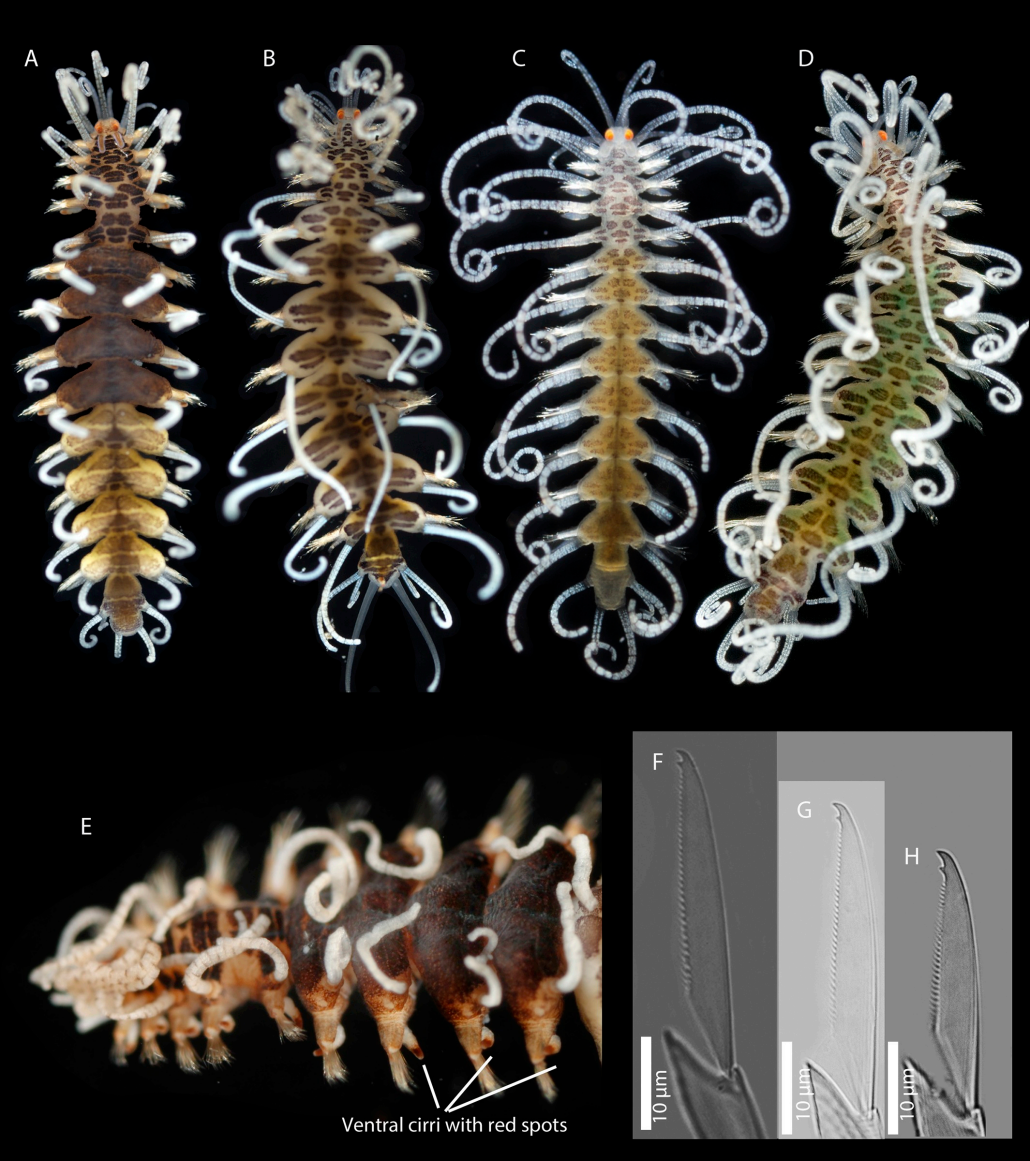
*Amblyosyllis ovei* n. sp. (A–E). Live specimens with slight differences in the colour pattern, dorsal view; A: MNCN 16.01/17985, B: MNCN 16.01/17986, C: MNCN 16.01/17983, D: MNCN 16.01/17984, E: MNCN 16.01/17985. (F) Dorsal chaeta, midbody segment. (G) Medial chaeta, midbody segment. (H) Ventral chaeta, midbody segment.

**Fig 14:**
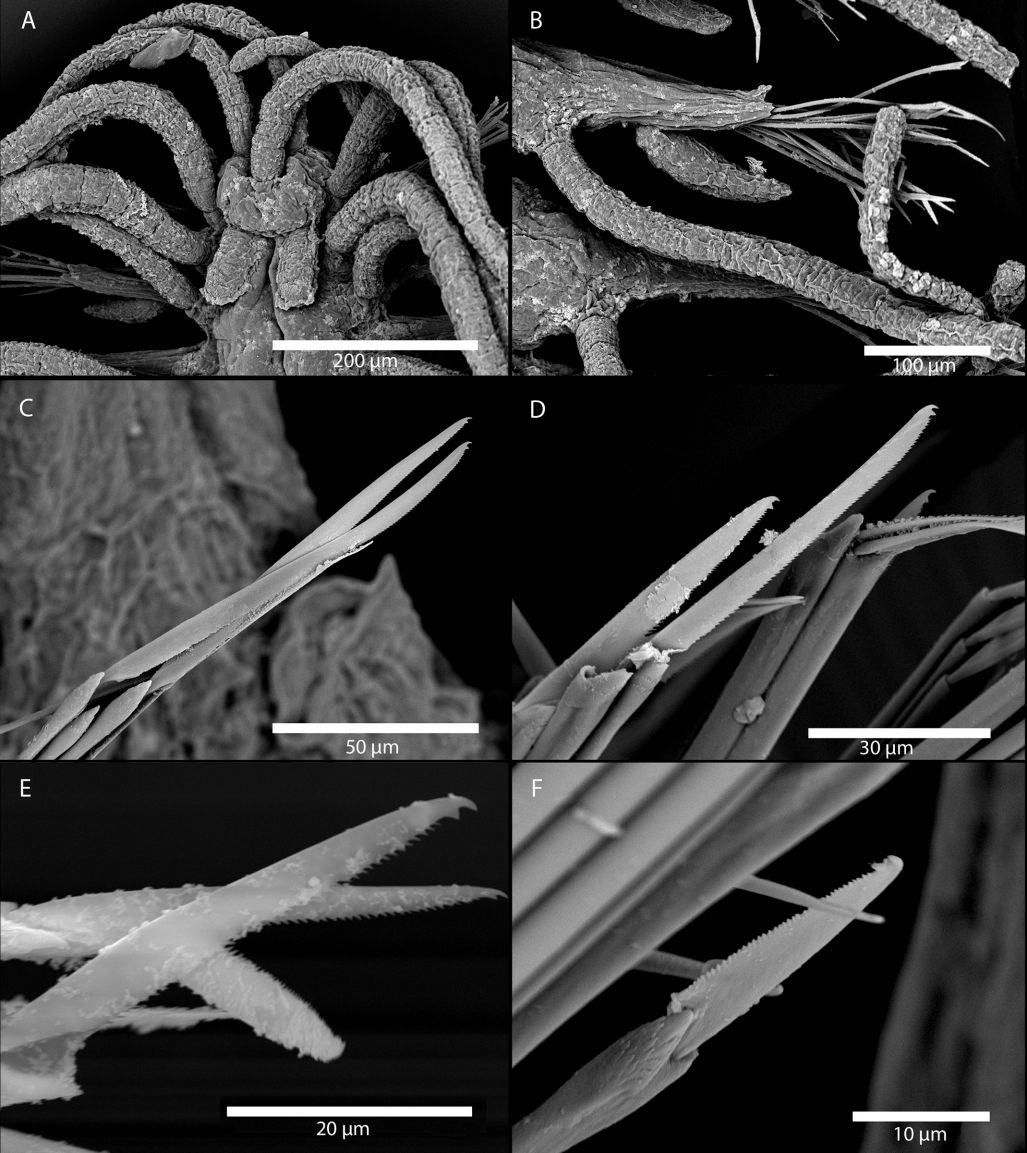
*Amblyosyllis ovei* n. sp. SEM. (A) Anterior end, dorsal view. (B) Anterior parapodia, dorsolateral view. (C) Dorsal chaetae, anterior segment. (D) Dorsal and medial chaetae, anterior segment. (E) Medial chaeta, anterior segment. (F) Ventral chaeta, anterior segment.

?*Amblyosyllis* sp. Human *et al*. 2013 [134]: 91, 1 Fig.

?*Amblyosyllis* sp. Turner Jr. *et al*. 2014 [135]: 92, 2 Figs.

?*Amblyosyllis* sp. Paresque *et al*. 2015 [136]: 321–325, Figs 12–14.

**Material examined:** Holotype (MNCN 16.01/17981) and two paratypes **(**MNCN 16.01/17980, MNCN 16.01/18462); additional material: MNCN 16.01/17983–6.

**Type locality:** Florida Keys, Summerland Key, Florida (USA, Gulf of Mexico)

**Locality of material examined:** Florida Keys, Summerland Key, Florida, USA, Gulf of Mexico (Caribbean Sea); Carrie Bow Cay (Ellen Cay), Twin Cays and Pelican Cays, Belize (Caribbean Sea).

**Habitat:** Intertidal (1–10 m); *Caulerpa* (Florida), and coarse sand and mangrove roots (Belize).

**Distribution**: Florida to ?northeast Brazil, ?Cayman Islands.

**Description:** Holotype incomplete, with 11 segments, 2.63 mm long, 0.55 mm wide. Colouration pattern with 6–8 dark spots per segment arranged symmetrically (Fig 13A–13D). Nuchal lappets long, reaching the end of the second chaetiger in specimens from Florida; they are slightly shorter in specimens from Belize (Fig 14A). Nuchal lappets with dark colouration (Fig 13A); especially marked in specimens from Florida. Dorsal cirri pseudoarticulated. Ventral cirri as long as parapodial lobes, spindle shaped, with dark reddish granular material inside (Fig 13E). Parapodia with several bidentate, long chaetae (Fig 14B–14F), around 10–25 on midbody parapodia. Distal tooth similar in shape or slightly larger than proximal one (Fig 13F–13H).

**Variation:** One specimen with midbody segments strongly pigmented dorsally (MNCN 16.01/17985, Fig 13A and 13E). Two specimens (MNCN 16.01/17983 and MNCN 16.01/17986) with dark colouration in dorsal cirri as transversal rings. Specimen (MNCN 16.01/17986) full of gametes in posterior segments. Paratype (MNCN 16.01/17980) with pharynx everted, trepan not observed.

**Remarks:** Some specimens (not related with locality: Florida or Belize) share exactly the same colour pattern, while others from the same locality show slight differences. The length of the nuchal lappets slightly differs in specimens from Florida and Belize, which might represent interpopulation variability. The only species previously reported from the Caribbean Sea is *A*. *rhombeata*, the type of the genus (see below, under Lineage 13). However, it shows a rhomboid or trapezoid black figure made of 6–8 transversal dark lines dorsally [2, 31], while in *A*. *ovei* n. sp. the dark areas are made of 6–8 short rounded dark spots. *Amblyosyllis* sp. from Paraíba, northeast Brazil [136], shows a pigmentation pattern similar to *A*. *ovei* n. sp. and thus, it is here tentatively considered the same species. *Amblyosyllis* sp. from San Andres (Colombia) photographed by Human *et al*. [134] and the ones from Cayman Islands photographed by Turner Jr. *et al*. [135] show the same colour pattern as *A*. *ovei* n. sp. specimens from Belize (Fig 14A), and are also tentatively referred to this species.

**Etymology:** This species is dedicated to Fredrik Pleijel’s stepson Ove Cedman Löfgren.

#### Lineage 7

***Amblyosyllis* sp. 4**

Fig 15

**Fig 15:**
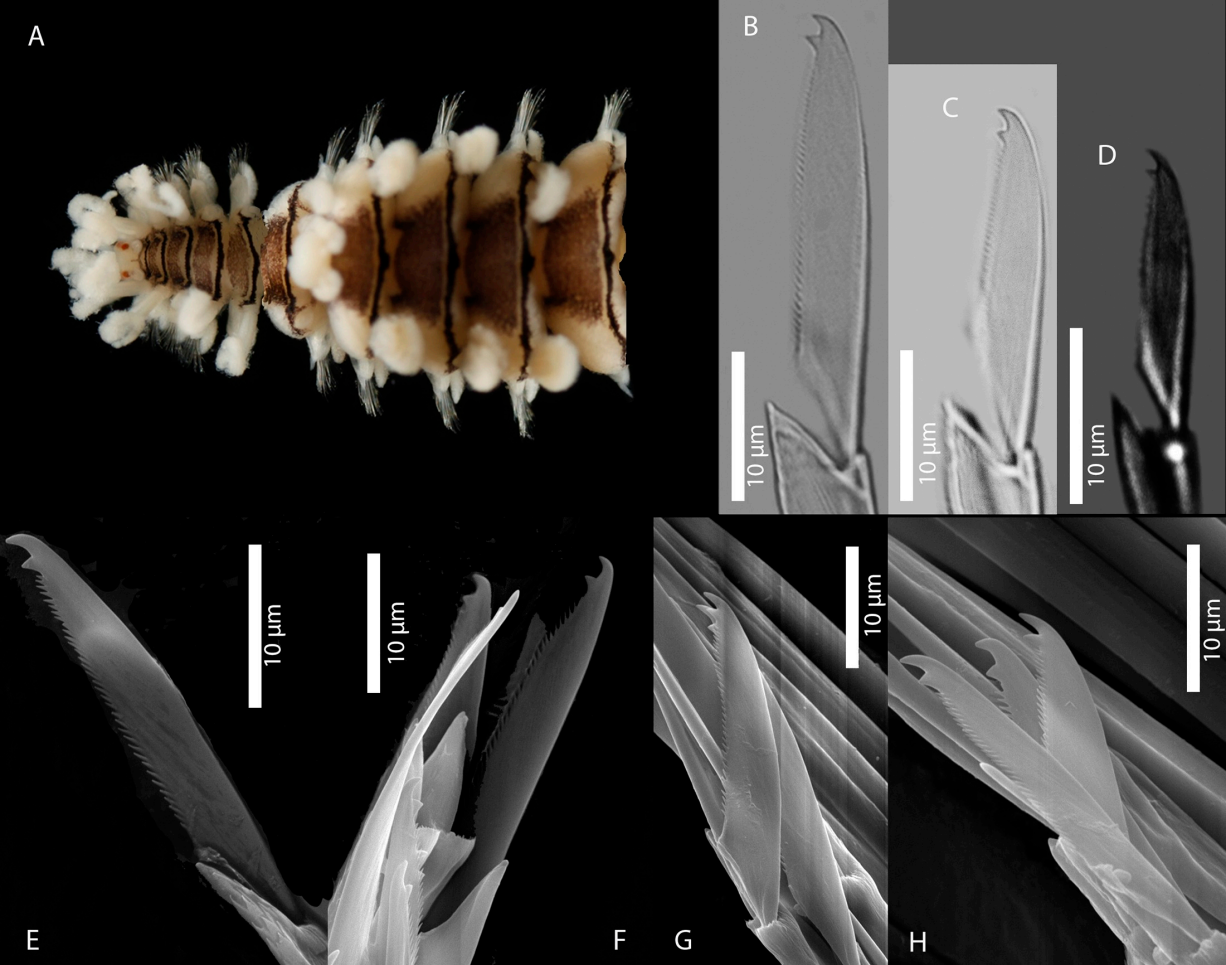
*Amblyosyllis* sp. 4. (A) Live specimen, dorsal view, MNCN 16.01/17987. (B) Dorsal chaeta, midbody segment. (C) Medial chaeta, midbody segment. (D) Ventral chaeta, midbody segment. (E, F) Dorsal chaetae, anterior segment, SEM. (G) Medial chaeta, anterior segment, SEM. (H) Ventral chaeta, midbody segment, SEM.

**Material examined:** One specimen (MNCN 16.01/17987).

**Locality of material examined:** Cape D’Aquilar, Lobster Bay, Hong Kong (South China Sea).

**Habitat:** 1 m; in *Corallina* sp.

**Description:** Anterior fragment of single specimen available 3.3 mm long, 1.4 mm wide. Colouration of each segment consisting in a thick dark transversal band in the anterior-most region and a transversal black line in the posterior-most region (Fig 15A). Nuchal lappets short, rounded. Parapodia with prechaetal digitiform conical papilla, longer in posterior parapodia; ventral cirri longer than parapodial lobes, spindle shaped. Parapodia with several short length bidentate chaetae (Fig 15B–15H), around 14–18 on midbody parapodia. Distal and proximal teeth approximately equivalent in size (Fig 15B–15D). Trepan not observed.

**Remarks:** Specimen with a characteristic colour pattern and short nuchal lappets, different to any other described species. However, there is only one specimen available, which prevents to be here described as a new taxon. The colour pattern and the short nuchal lappets approach the specimen to one of the five Japanese colour morphotypes of *Amblyosyllis* described by Imajima [42], and they could represent the same species (see also the remarks for *Amblyosyllis hectori* n sp.).

#### Lineage 8

***Amblyosyllis madeirensis* Langerhans**, **1879**

Figs 16 and 17

**Fig 16:**
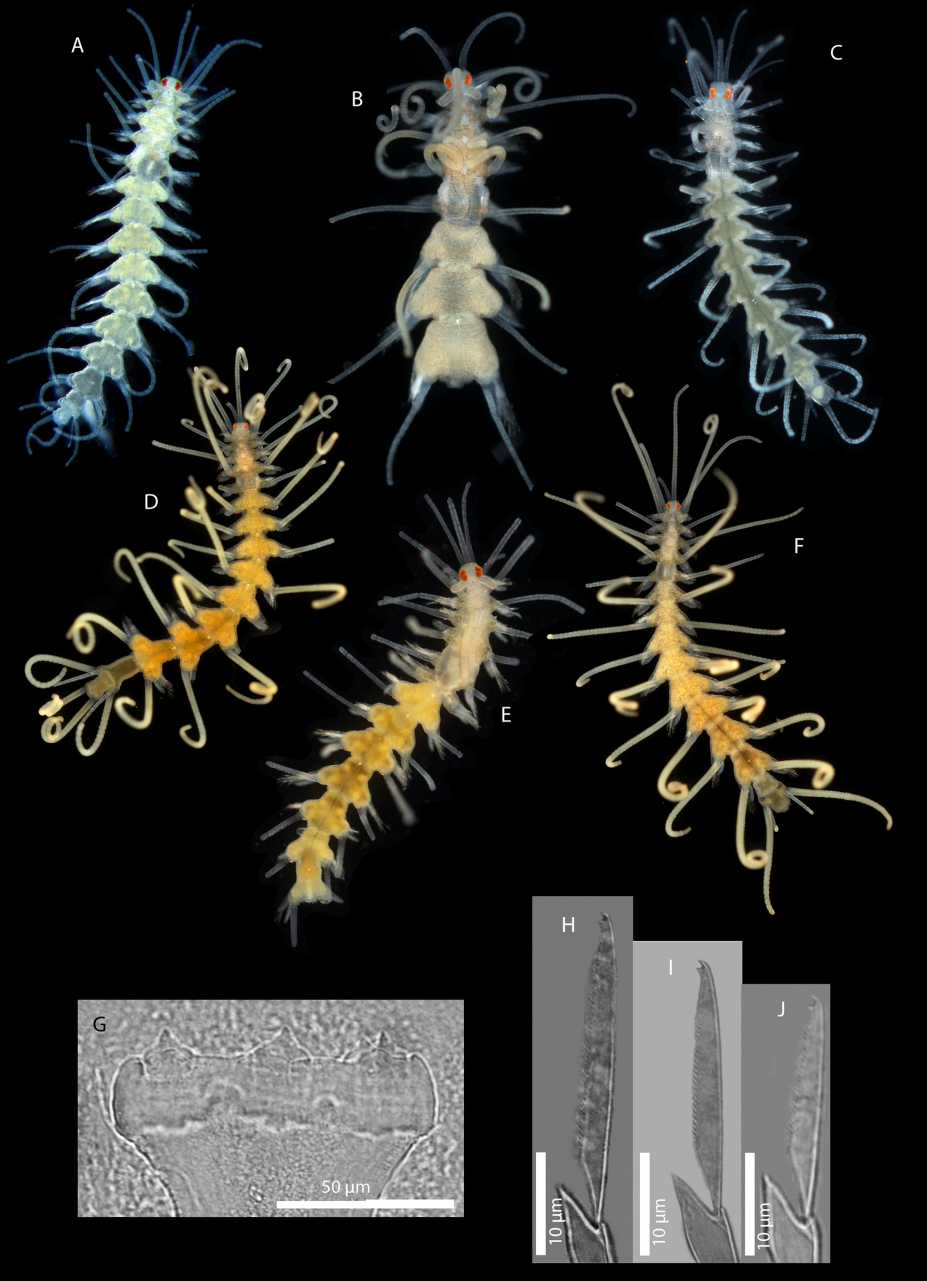
*Amblyosyllis madeirensis*. (A–F) Live specimens with slight differences in the colour pattern, dorsal view. A: MNCN 16.01/17988, B: MNCN 16.01/17991, C: MNCN 16.01/17990, D: MNCN 16.01/17993, E: MNCN 16.01/17989, F: MNCN 16.01/17992. (G) Trepan. (H) Dorsal chaeta, midbody segment. (I) Medial chaeta, midbody segment. (J) Ventral chaeta, midbody segment.

**Fig 17:**
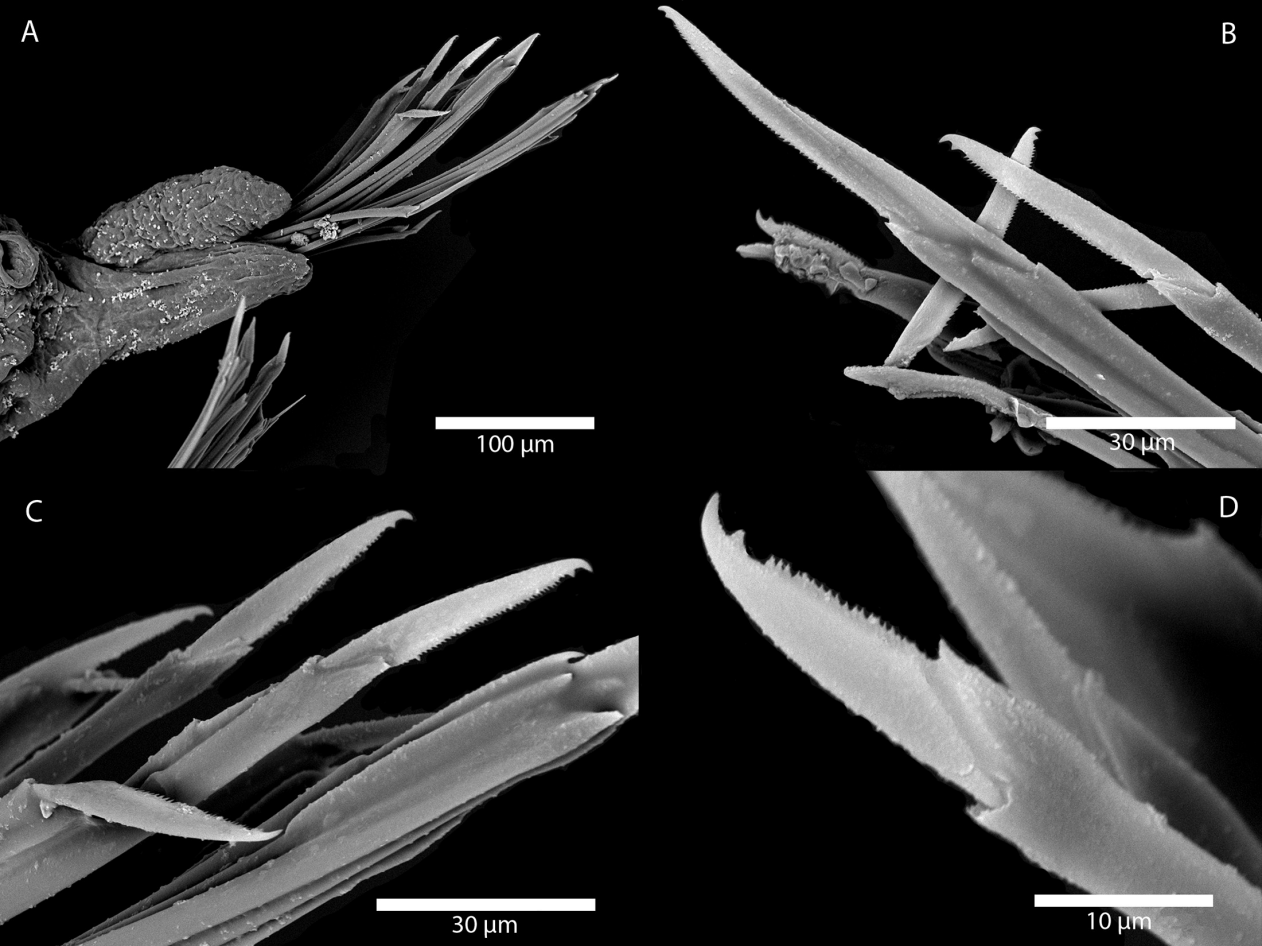
*Amblyosyllis madeirensis*. SEM. (A) Anterior parapodium, ventral view. (B) Dorsal chaetae, anterior segment. (C) Medial chaetae, anterior segment. (D) Ventral chaeta, anterior segment.

*Amblyosyllis madeirensis* Langerhans, 1879 [29]: 561, plate XXXII, Fig 19.

**Type locality:** Madeira Island, Portugal (Northeastern Atlantic Ocean).

**Material examined:** Six specimens (MNCN 16.01/17989–93, 18462).

**Locality of material examined:** Banyuls-sur-Mer, France (Mediterranean Sea); Istria, Croatia (Adriatic Sea); Porto Moniz, Madeira (Atlantic Ocean).

**Habitat:** Between 15–62 m; mixed sample of hard and soft substrate, amongst tunicates, shells with epifauna, hydroids and sponges.

**Distribution:** Madeira (Atlantic Ocean); Croatia and Italy (Mediterranean Sea).

**Description of material examined:** Best preserved specimen (MNCN 16.01/17991) with five anterior chaetigers, 1.25 mm long, and 0.68 mm wide. Colour pattern variable and not well-defined, most of the specimens with a variable number of transversal lines, but in some individuals, the central region of each chaetiger is colourless (Fig 16A–16F). Nuchal lappets clearly ciliated, long in some specimens, reaching the end of first chaetiger, in others they are smaller and rounded (Fig 16D). Dorsal cirri long and coiled over the dorsum; in some specimens clearly distally articulated. Ventral cirri longer than parapodial lobes, with dark granular material inside. Parapodia with several long to medium length bidentate chaetae (Figs 16H–16J and 17A–17D), around 14–16 on midbody parapodia. Distal and proximal teeth approximately equivalent in size. Trepan with six pentacuspid teeth (Fig 17G); lateral cusps in each tooth difficult to discern.

**Remarks:** The colour pattern and the length and shape of the nuchal lappets are variable. The distribution and morphology of the lineage coincide with those described for *A*. *madeirensis* [29].

**Etymology:** The specific epithet *madeirensis* refers to the type locality of the species, Madeira Island (Atlantic Ocean).

#### Lineage 9

***Amblyosyllis clarae* n. sp.**

Figs 18 and 19

**Fig 18:**
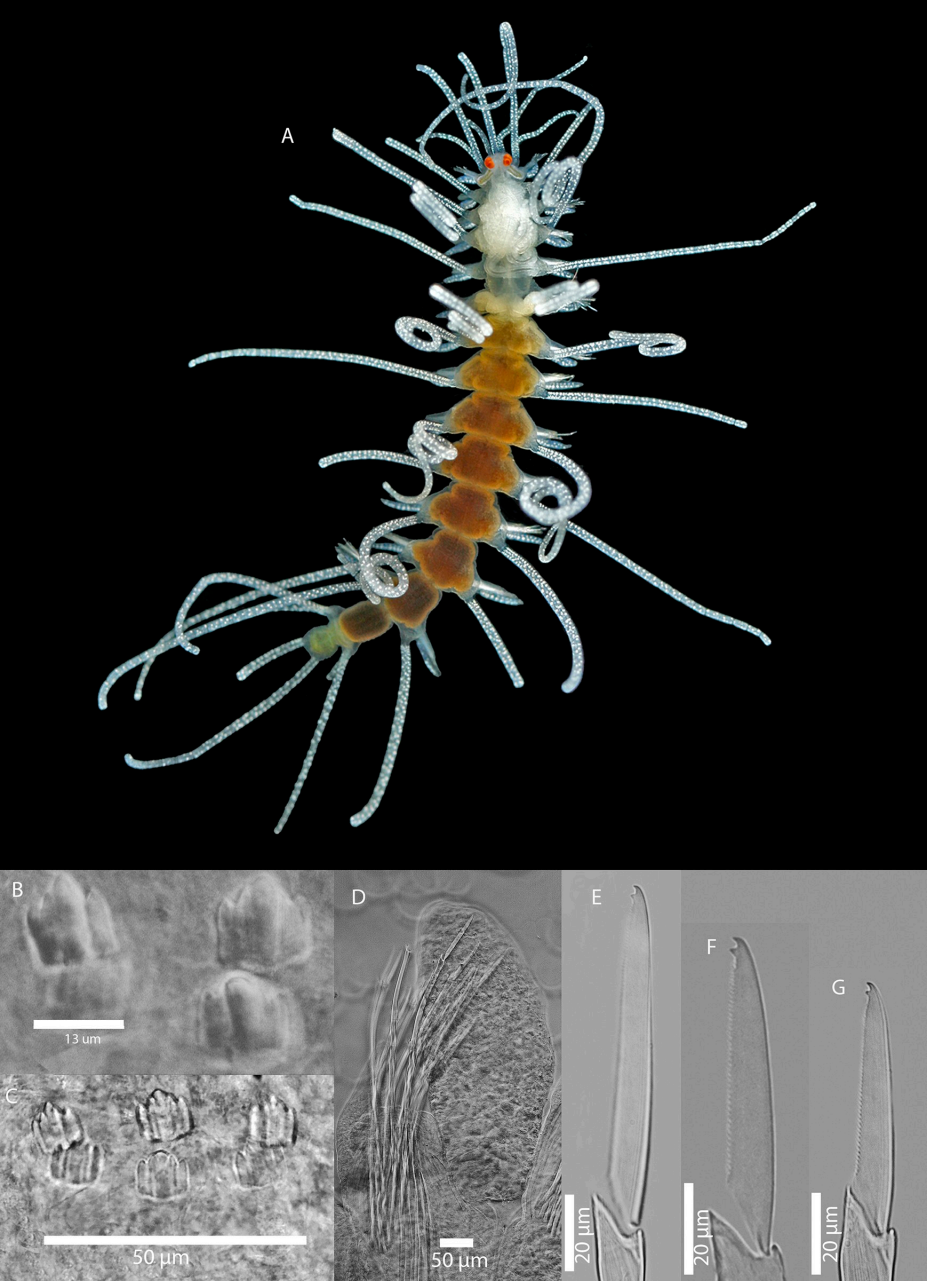
*Amblyosyllis clarae* n. sp. (A) Live specimen, dorsal view, MNCN 16.01/17994. (B) Trepan teeth. (C) Complete trepan. (D) Midbody chaetiger and ventral cirri. (E) Dorsal chaeta, midbody segment. (F) Medial chaeta, midbody segment. (G) Ventral chaeta, midbody segment.

**Fig 19:**
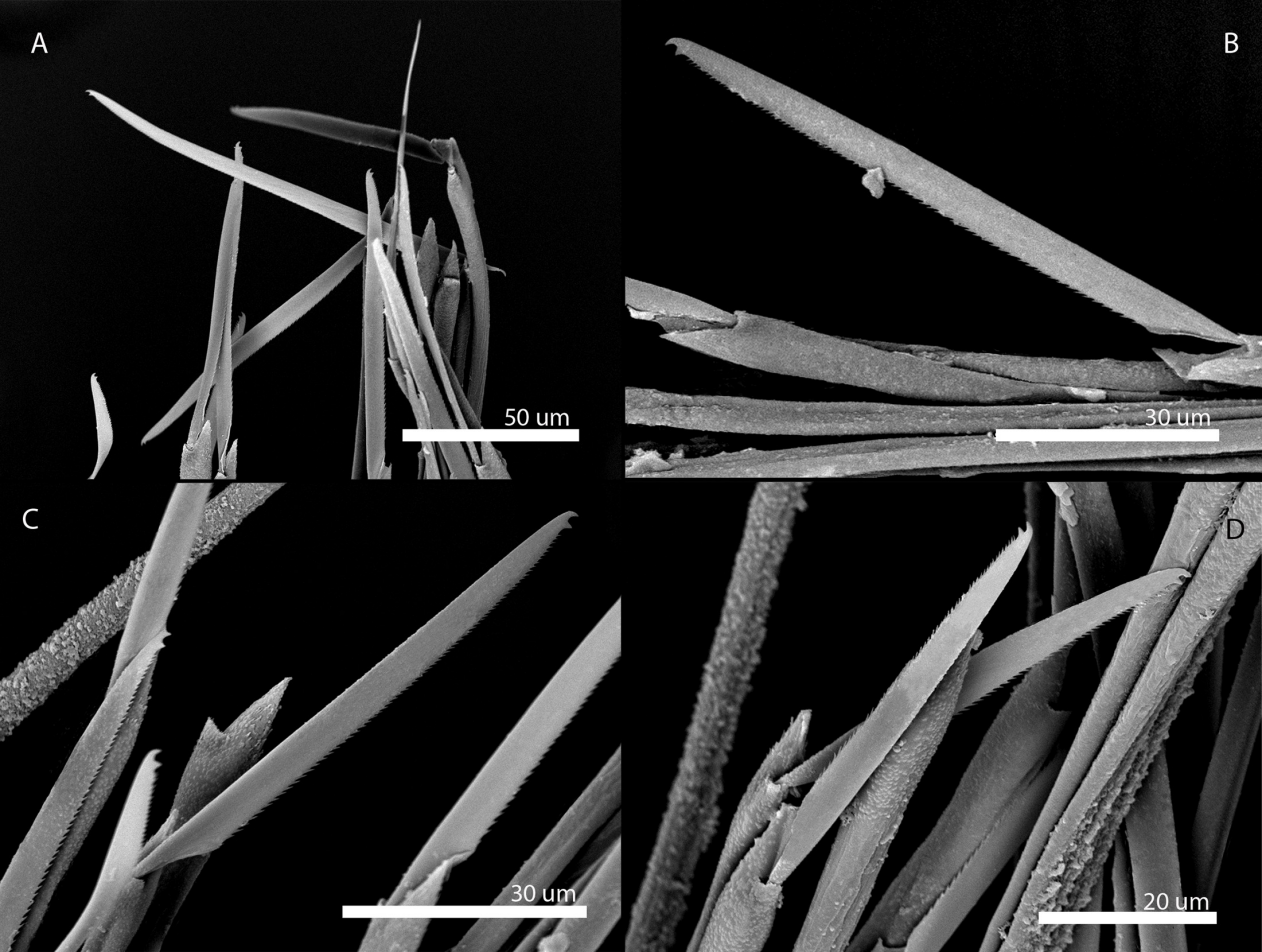
*Amblyosyllis clarae* n. sp. SEM. (A–C) Dorsal chaetae, anterior segment. (D) Dorsal to medial chaetae, anterior segment.

**Material examined:** Holotype (MNCN 16.01/17994) and paratypes (MNCN 16.01/17995–7). Additional material **(**MNCN 16.01/17998–18001), two specimens fixed in formalin (MNCN 16.01/18081, MNCN 16.01/18082).

**Type locality:** Trondheim, Norway (Norwegian Sea).

**Locality of material examined:** Trondheim, Norway (Norwegian Sea); Hjeltefjorden, Bergen, Norway (North Sea).

**Habitat:** Between 50–250 m; on *Lophelia*, *Geodia*, *Phakellia*, *Sabella* tubes, and *Pandalina*.

**Description**: Holotype incomplete with 6 chaetigers, 2.48 mm long, 1.28 mm wide. Anterior segments whitish, brown-yellowish segments posterior to the proventricle (Fig 18A). Dorsal surface densely covered by bunches of cilia. Two rounded nuchal lappets, extending over half of the first chaetiger. Dorsal cirri distally articulated. Distinct prechaetal digitiform papilla, longer and pointed in posterior chaetigers; ventral cirri slightly longer than parapodial lobes, round shaped (Fig 18D), with dark granular material inside. Parapodia with several long bidentate chaetae (Figs 18E–18G and 19A–19D), around 18–20 on midbody parapodia. Distal and proximal teeth approximately equivalent in size (Figs 18E–18G and 19A–19D). Paratypes (MNCN 16.01/17995 and MNCN 16.01/17996) with pharynx everted, trepan with six pentacuspid teeth (Fig 18B and 18C).

**Variation**: Specimens (e.g. those from Bergen) show small dark spots occasionally forming transversal bands over dorsum (e.g. MNCN 16.01/17998, MNCN 16.01/18001). One specimen (MNCN 16.01/18000) with dark colour in nuchal lappets.

**Remarks:** There are some slight differences in the colour pattern between specimens from Bergen and Trondheim that are interpreted as intraspecific variability. Chaetae shape is similar to its sister group *A*. *lineata*; however, the colour pattern of both species is clearly different.

**Etymology:** This species is dedicated to Clara Bleidorn Aguado, who was born when the first version of this manuscript was written. The yellow-brownish ground colour of this species (as seen in Fig 18A) resembles the hair colour of Clara.

#### Lineage 10

***Amblyosyllis lineata* Grube**, **1863**

Figs 20 and 21

**Fig 20:**
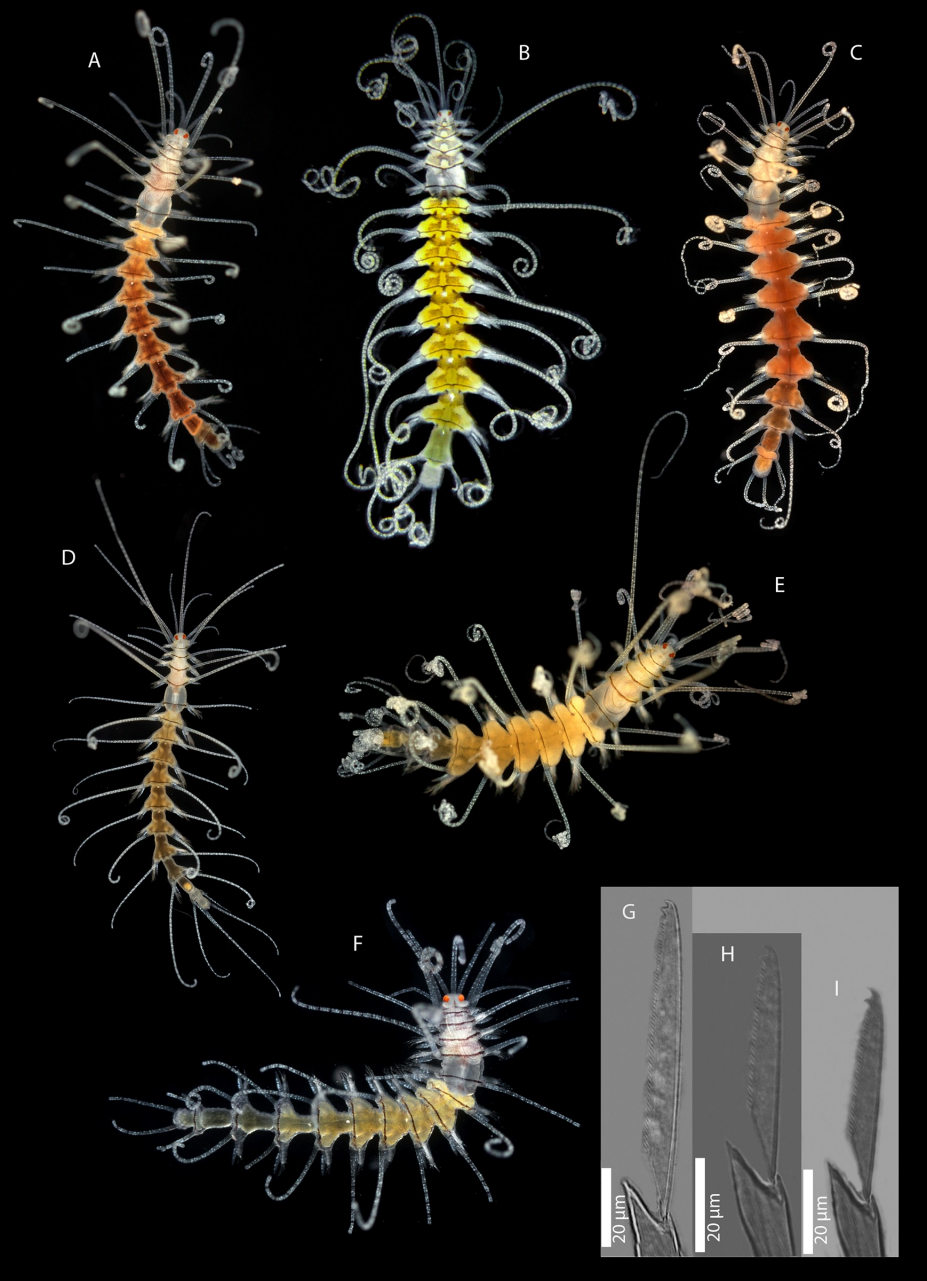
*Amblyosyllis lineata*. (A–F) Live specimens with differences in the colour pattern, dorsal view; A: MNCN 16.01/18004, B: MNCN 16.01/18008, C: MNCN 16.01/18011, D: MNCN 16.01/18012, E: MNCN 16.01/18003, F: MNCN 16.01/18009. (G) Dorsal chaeta, midbody segment. (H) Medial chaeta, midbody segment. (I) Ventral chaeta, midbody segment.

**Fig 21:**
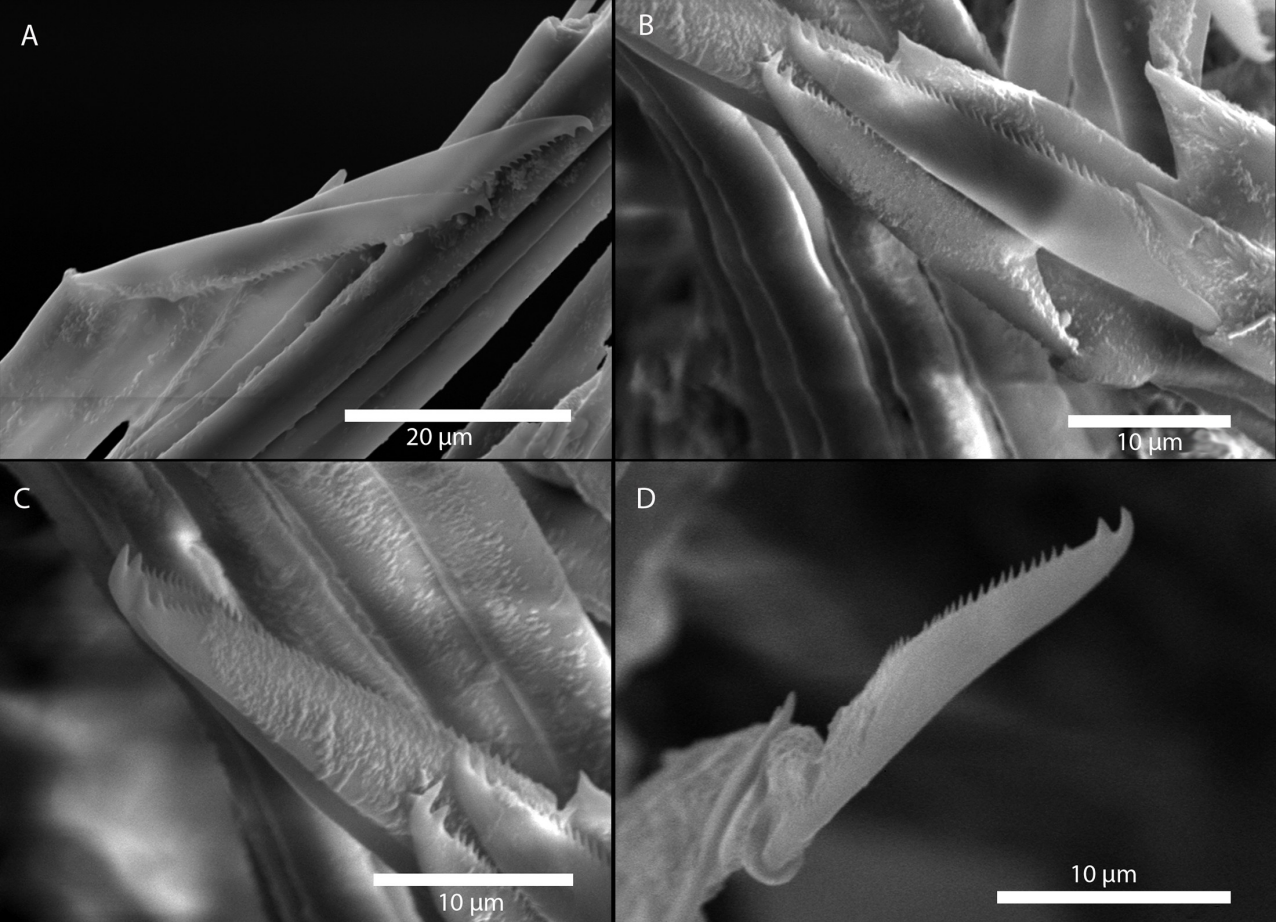
*Amblyosyllis lineata*. SEM. (A–C) Medial chaetae, midbody segment. (D) Medial to ventral chaeta, midbody segment.

*Amblyosyllis lineata* Grube, 1863 [74]: 48–49, plate V, Fig 1.

*Amblyosyllis algefnae* Viguier, 1886 [137]: 425–426, plate XXVII, Figs 5–9.

*Amblyosyllis madeirensis* San Martín, 2003 [18]: 96–99, Figs 41, 42; not Langerhans, 1879 [29].

**Type locality:** Mali Lošinj (= “Lussin Piccolo”), Croatia (Adriatic Sea).

**Material examined:** Two syntypes (Q3450 MfN, coll. Grube); 1 specimen **(**Q4363 MfN, coll. Grube); 11 specimens (MNCN 16.01/18002–12); one specimen preserved in formaline (MNCN 16.01/18083); slides (MNCN 16.01/18464–66).

**Additional material examined.** *Amblyosyllis madeirensis* (San Martín, 2003; not Langerhans, 1879). MNCN 16.01/6545, 1 specimen, Menorca, Baleares, Spain (Mediterranean Sea). MNCN 16.01/6548, 1 specimen, Columbretes, Spain (Mediterranean Sea). MNCN 16.01/6546, 1 specimen, Mallorca, Baleares, Spain (Mediterranean Sea).

**Locality of material examined:** Banyuls-sur-Mer, France (Mediterranean Sea); Ischia, Italy (Tyrrhenian Sea); Istria, Croatia (Adriatic Sea).

**Habitat:** Between 15–25 m; coralligenous, shells, ascidians, and sponges.

**Distribution:** France, Croatia, Italy and Spain (Mediterranean Sea).

**Description of material examined:** Best preserved specimen (MNCN 16.01/18009) incomplete, with 9 segments, 13.2 mm long, 0.43 mm wide. Colour pattern variable, some specimens with one dark transversal line in the posterior-most region of each chaetiger (Fig 20A and 20C–20F), others (e.g. MNCN 16.01/18011) with two dark anterior areas on each segment, a triangular spot, posteriorly oriented, in the middle of the transversal line, one additional longitudinal line at the base of each cirrophore and two spots in the posterior margin of each segment, posterior to the transversal dark line (Fig 20B). Two small rounded nuchal lappets, extending over a small portion of the first chaetiger. Dorsum covered by bunches of cilia (MNCN 16.01/18009), specimen MNCN 16.01/18003 with cilia only on the anterior segments. Dorsal and ventral cirri producing secretions. Dorsal cirri coiled with transversal dark lines in some specimens (MNCN 16.01/18002, 18004, 18006). Ventral cirri slightly longer than parapodial lobes. Parapodia with several long bidentate chaetae (Figs 20G–20I and 21A–21D), around 6–8 on midbody parapodia. Distal and proximal teeth approximately equivalent in size (Figs 20G–20I and 21A–21D). Trepan with six pentacuspid teeth.

**Remarks:** Name reinstated. This species was synonymized with *A*. *formosa* [32] and this synonymy was later maintained [30]. Langerhans [29] described the species as having 12 teeth in the trepan, but based on specimens from Madeira. The type locality, chaetae and colour pattern coincide with our specimens and with those identified as *A*. *madeirensis* by San Martín [18].

**Etymology:** The specific epithet of *Amblyosyllis lineata* is a Latin adjective meaning ‘lined’, and refers to the presence of a transversal dorsal black line on most segments. According to Viguier [137], *Amblyosyllis algefnae* was named after the artillery battery located in the middle of Algiers harbour when the species was collected, known as Al-Djefna. It was briefly cited in the 1894’s fictious novel “The Great War in England in 1897” by William Le Queux: “In a few minutes the Al-Djefna Battery in the centre of the harbour replied”. The battery was built on a submarine rock of the same name originally located opposite the mouth of the harbour and later incorporated in its interior upon its expansion, being finally integrated in the passenger’s wharf of Al Djefna (Algiers, Algeria).

#### Lineage 11

***Amblyosyllis* sp. 5**

**Material examined:** One specimen (MNCN 16.01/18013).

**Locality:** Coquimbo area, Chile (South Pacific Ocean).

**Habitat**: Intertidal.

**Remarks:** Small specimen, a juvenile or a regenerating adult. The specimen has a rudimentary pygidium and posterior segments not well developed. Chaetae resembling those of *Amblyosyllis emilioi* n. sp. The material is in poor condition to enable a proper description of the species.

#### Lineage 12

***Amblyosyllis emilioi* n. sp.**

Fig 22

**Fig 22:**
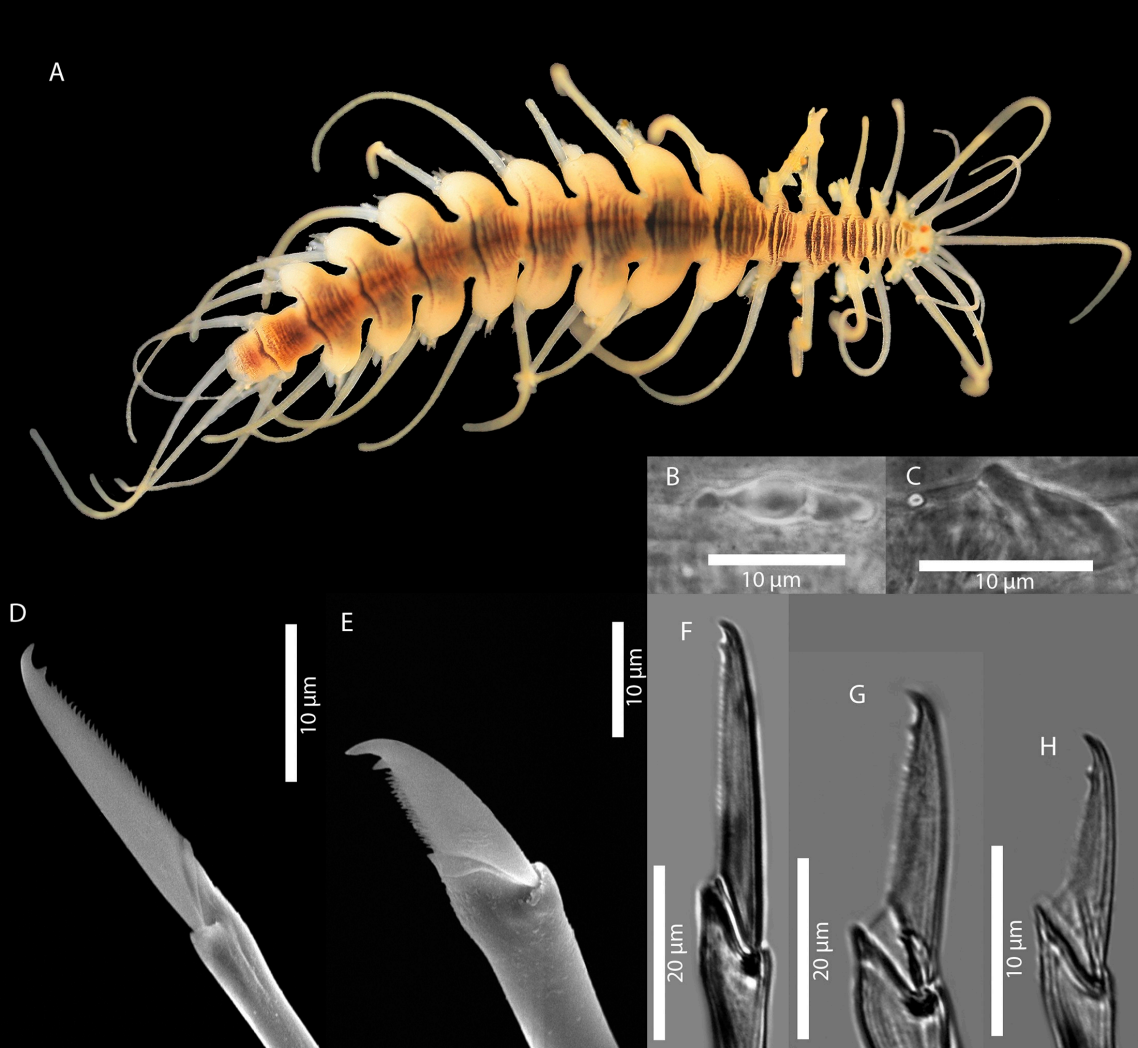
*Amblyosyllis emilioi* n. sp. (A) Live specimen, dorsal view, MNCN 16.01/18018. (B, C) Trepan, cusps difficult to discern. (D) Dorsal chaeta, midbody segment, SEM. (E) Ventral chaeta, midbody segment, SEM. F. Medial chaetae, midbody segment. (G) Medial chaeta, midbody segment. (H) Ventral chaeta, midbody segment.

**Material examined:** Holotype (MNCN 16.01/18018) and five paratypes (MNCN 16.01/18014, 18016–17, 16463).

**Comparative material examined.** *Amblyosyllis granosa* 1 specimen (SMNH 152708), Chile, Ancud Gulf, East coast of Chiloé Is., Canal, stones with algae, sta. M63a; 1 specimen (SMNH 152709), Chile, Canal Chacao, W of Rocas Amazonas, 40 m, stones, sta. M94. Lund University Chile Expedition 1948–49.

**Type locality:** Valdivia area, Chile (South Pacific Ocean).

**Locality of material examined:** Coquimbo and Valdivia area, Chile (South Pacific Ocean).

**Habitat:** Intertidal (5–7 m); among *Chaetopterus* tubes.

**Description:** Holotype 8 mm long, 2.13 mm wide. With a variable number of transversal dark lines per segment, with the posterior-most transversal line of each chaetiger usually thicker than the rest (Fig 22A). Segments secondarily annulated, with dorsal bunches of cilia on anterior segments. Nuchal lappets extending over the beginning of first chaetiger, with dark colouration. Distinct prechaetal, digitiform papilla large, as long as half-length of parapodia. Ventral cirri slightly longer than parapodial lobes, cirriform. Parapodia with several medium length bidentate chaetae (Fig 22D–22H), around 10–15 on midbody parapodia. Distal tooth considerably larger than proximal one (Fig 22D–22H). Trepan with six teeth, cusps difficult to discern (Fig 22B and 22C), but probably pentacuspid.

**Variation:** Large specimens, massive, several epigamic with gametes inside. Some specimens with colouration only in anterior segments. Paratype MNCN 16.01/180015 with shorter prechaetal papillae.

**Remarks:** Several specimens bear small ciliate protists, especially on anterior segments. These specimens resemble *Brachysyllis infuscata* (Ehlers, 1901) [138], redescribed by Aguado *et al*. [139], in the large body size, the segments secondarily annulated, and the presence of bunches of cilia, but differ in their respective generic diagnostic characters. The chaetae are similar in shape and length to specimens from Galápagos identified as *Amblyosyllis granosa* Ehlers, 1897 [39] by Westheide [140], which differ in having a colour pattern formed by squares. *Amblyosyllis granosa* was described without colour pattern (brown-goldish colouration), chaetae longer than those of the specimens from Galápagos, and absence of trepan [39]. It is a species with a wide distribution (Table 1), among others, from the East Pacific, in South America (including the original description [39]) and South West Pacific (New Zealand and Australia). However, the material from different localities shows clear differences. For instance, Verdes *et al*. [40], revised material of *A*. *granosa* from Australia finding two possible morphotypes with different chaetal shape. In our opinion, the reports from areas distant to South of Chile probably correspond to different species. The specimens from Galápagos, identified by Westheide [140] differ from the original description in the colour pattern and chaetal length, and could well be a different species too. Westheide described a trepan with six monocuspid teeth [140]. Specimens of *A*. *granosa* from the SMNH coincide well with the original description, brown goldish, without any other colour pattern. The chaetae are longer than those of the specimens identified herein, but similar in shape.

**Etymology:** This species is dedicated to Emilio Barcia, an excellent person, surgeon and historian in Ponferrada, El Bierzo, Spain. He passed away at 71 years old while studying his biology degree; he was very passionate about animals and nature itself.

#### Lineage 13

***Amblyosyllis rhombeata* Grube & Ørsted *in* Grube**, **1857**

Fig 23

**Fig 23:**
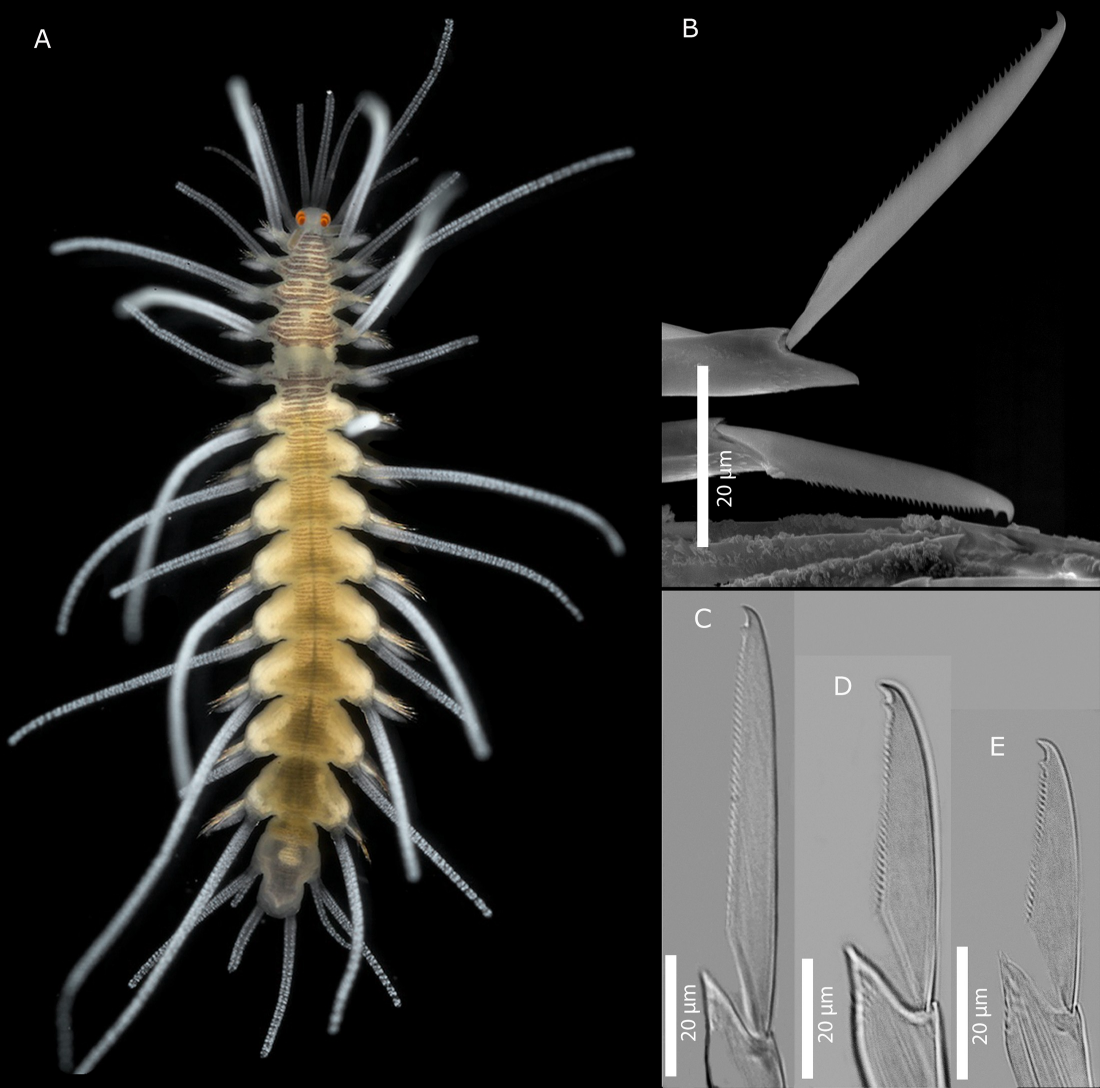
*Amblyosyllis rhombeata* n. sp. (A) Live specimen, dorsal view, MNCN 16.01/18023. (B) Dorsal chaeta, midbody segment, SEM. (C) Dorsal chaeta, midbody segment. (D) Medial chaeta, midbody segment. (E) Ventral chaeta, midbody segment.

*Amblyosyllis rhombeata* Grube & Ørsted *in* Grube, 1857 [2]: 186.

*Amblyosyllis rhombeata* Wolff & Petersen, 1991 [31]: 678, Fig 3.A.

**Type locality:** Saint Croix, U.S. Virgin Islands (Caribbean Sea).

**Material examined:** Seven specimens (MNCN 16.01/18019–25).

**Locality of material examined:** Florida Keys, USA, Gulf of Mexico (Caribbean Sea); Carrie Bow Cay (Ellen Cay), Belize (Caribbean Sea).

**Habitat:** Intertidal; on *Caulerpa* and coarse sand.

**Distribution:** Atlantic Ocean: Gulf of Mexico and Caribbean Sea.

**Description of material examined:** Best preserved specimen (MNCN 16.01/18025) incomplete, 1.38 mm long, 1 mm wide. With a variable number of transversal dark lines per segment (Fig 23A). Dark lines, in some segments, forming a rhombic shape. Two elongated nuchal lappets extending over first chaetiger. Dorsum covered by bunches of cilia. Prechaetal digitiform papilla elongated in posterior parapodia; ventral cirri longer than parapodial lobes. Parapodia with several long to medium length bidentate chaetae (Fig 23B–23E), around 18–23 on midbody parapodia. Distal tooth larger than proximal one (Fig 23B–23E). Trepan not observed.

**Remarks:** No differences were observed between the specimens from Florida and Belize. The dorsal gradation in the length of chaetae is not as pronounced as in other species of the genus. The original description of *A*. *rhombeata* does not include illustrations, a more detailed description does not exist, and the type series was never located by posterior authors and may be lost (D. Eibye-Jacobsen, pers. comm., 02 May 2018 [31, 141]). However, the species was described with a dorsal colour pattern formed by a rhomboid or trapezoid black figure made of 6–8 transversal dark lines by Grube & Ørsted [2]). Moreover, Wolff & Petersen ([31]: Fig 3A, as “*Melanosyllis microcephala*”) provided an unpublished pencil and watercolour sketch made by A.J. Ørsted based on a live specimen, where it is possible to observe the rhomboid colour pattern formed by the several transversal dark lines reported in the original description. The described colour pattern approaches the present description, being the single difference the presence of black eyes [2, 31], instead of red (Fig 23A). This difference is considered to be irrelevant, and the present specimens are considered to represent the long sought type species of the genus, *A*. *rhombeata*. The chaetal shape is similar to *Amblyosyllis ovei* n. sp., from the same geographic regions, but the colour pattern is very different (see above). The *Amblyosyllis* sp. from Florida that appears in the Scripps Oceanography web page, pictured by G. Rouse, shows the same colour pattern and should be identified as *A*. *rhombeata*.

**Etymology:** The specific epithet *rhombeata* is a Latin adjective meaning ‘rhombic’, and refers to the dorsal coloration pattern of the segments of the species, composed by 6 to 8 transversal dark streaks forming a black trapezoid to rhomboid figure (Grube & Ørsted [2]).

#### Lineage 14

***Amblyosyllis hectori* n. sp.**

Fig 24

**Fig 24:**
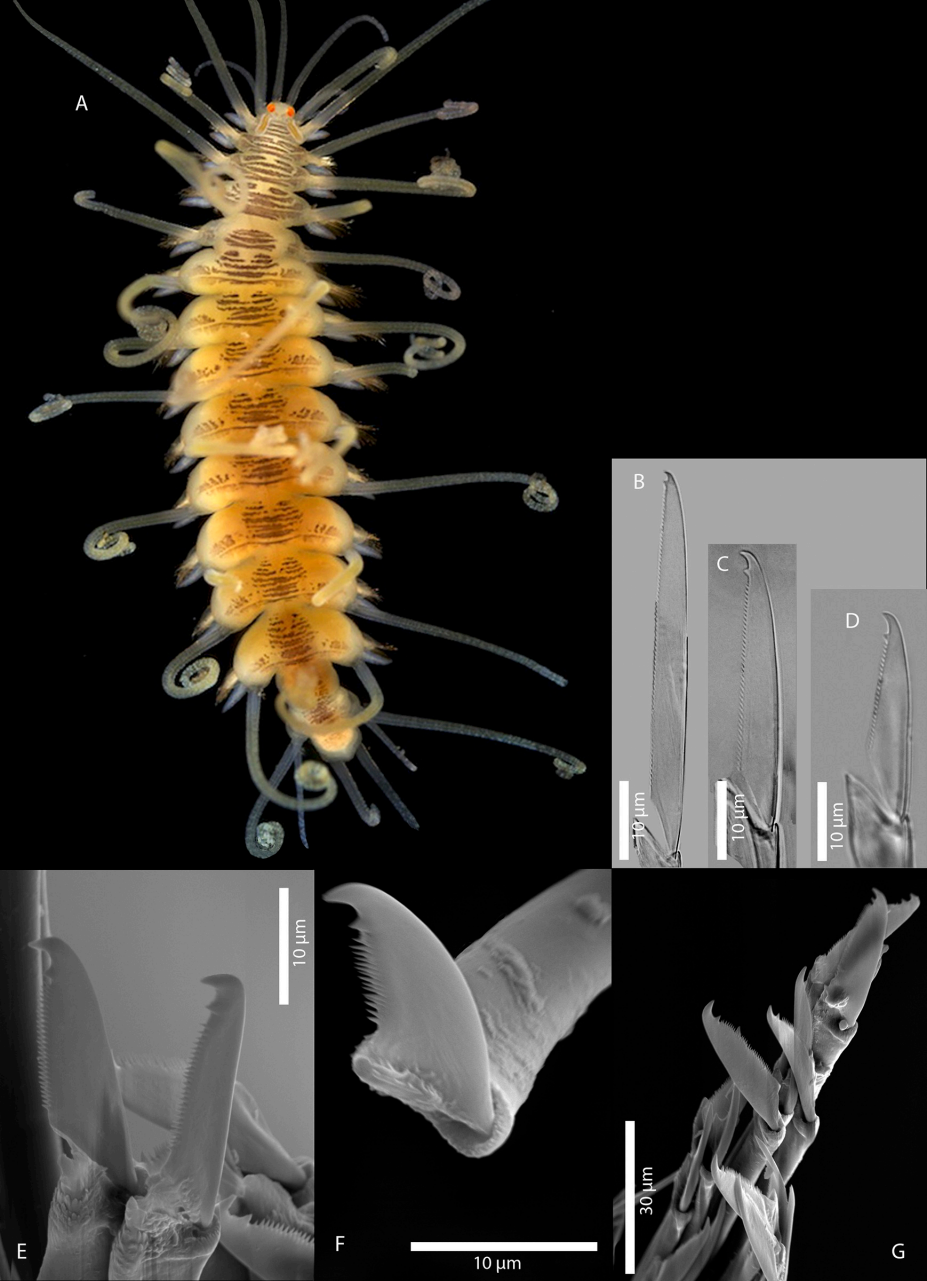
*Amblyosyllis hectori* n. sp. (A) Live specimen, dorsal view, MNCN 16.01/18026. (B) Dorsal chaeta, midbody segment. (C) Medial chaeta, midbody segment. (D) Ventral chaeta, midbody segment. (E–G) Chaetae, midbody segment, SEM.

*Amblyosyllis speciosa* Dorsey, 1978 [142]: 22–24, Fig 1a–c; not Izuka, 1912 [3].

**Material examined:** Holotype (MNCN 16.01/18027) and paratype (MNCN 16.01/18026).

**Type locality:** San Diego, California, USA (North Pacific Ocean).

**Habitat:** 18 m; on *Macrocystis* holdfasts.

**Description:** Holotype incomplete, 1.5 mm long, 1 mm wide. With several transversal lines per segment and some longitudinal lines on each side of the trapezoidal segment (Fig 24A); which leave a colourless triangular area in the beginning of each segment. Two elongated nuchal lappets extending over first chaetiger. Segments secondarily annulated. Dorsum covered by bunches of cilia. Prechaetal digitiform papilla elongated in posterior parapodia; ventral cirri similar in length to parapodial lobes. Parapodia with several long to medium length bidentate chaetae (Fig 24B–24G), around 20–25 on midbody parapodia. Distal tooth larger than proximal one (Fig 24B–24G). Trepan not observed.

**Remarks:** Chaetae similar to *A*. *anae* n. sp. and *A*. *speciosa* [3]. However, the colour pattern does not coincide with *A*. *anae* n. sp., neither with any of those of *A*. *speciosa* from Japan [3, 42]. Izuka [3] described *A*. *speciosa* from Misaki with “Colour of living specimens [being] purplish brown dorsally, with a median longitudinal yellowish patch in each segment, except on anterior most six segments and the anal segment; on the sixth segment a broad yellowish transverse band; posterior half of penultimate and anal segments lighter coloured. All the tentacles and cirri are milk-white” (Fig 1D). In 1964, Imajima & Hartman [11] synonymize *A*. *speciosa* and *A*. *nigrolineata*, another species described from Misaki [82]. These authors, based on previous descriptions and specimens collected off Shirikishinai (140 m depth), considered that *A*. *speciosa* had three different colour patterns: (i) dark purple with a white spot at the anterior part of all segments except the first and sixth segments; (ii) dark brown, saddle shaped band across the dorsum of each visible segment; and (iii) pale brown bands across the dorsum alternating with a pale band at segmental constrictions”. In 1966, Imajima [42] raised the number of colour patterns to five by adding: (iv) brownish purple with a white transverse band across between the dorsal cirri, and posterior region of the white band being darker than the anterior (corresponding with *A*. *nigrolineata* as described by Okada [82]); and (v) a brown transverse band with a median tubercle midway between the dorsal cirri, from Misaki. We suggest that these five different colour patterns very probably represent five different species from Japan and nearby waters. Unfortunately, Nishi & Tanaka [143] did not find the type of *A*. *speciosa* among Izuka’s polychaete collection deposited at the University Museum, University of Tokyo, adding “Unfortunately, the types of other species could not be found, possibly because they were discarded or destroyed by the Great Kanto Earthquake of 1923.”

In 1978, Dorsey identified as “*A*. *speciosa”* 28 specimens from California based on “the number and structure of pentacuspid teeth and the square shape of the fifth segment” [142]. However, the colour pattern did not match with any of the five described for *A*. *speciosa* from Japan, which was attributed to another possible variation within the same species, following Imajima & Hartman [11] and Imajima [42]. Since then “*A*. *speciosa”* has been used to identify specimens of *Amblyosyllis* with several different colour patterns along the Pacific coast of the USA, many of them fitting the colour pattern of *A*. *nigrolineata* (see the remarks for this species), while others fit *A*. *hectori* n. sp., or the specimens of Lineage 17 (see below).

The specimens from California studied herein match the colour pattern of those studied by Dorsey [142], and these are thus considered as belonging to *A*. *hectori* n. sp.

**Etymology:** This species is dedicated to Héctor Aguado Molina. The ground colour of this species (as seen in Fig 24A) resembles the hair colour of Héctor.

#### Lineage 15

***Amblyosyllis anae* n. sp.**

Fig 25

**Fig 25:**
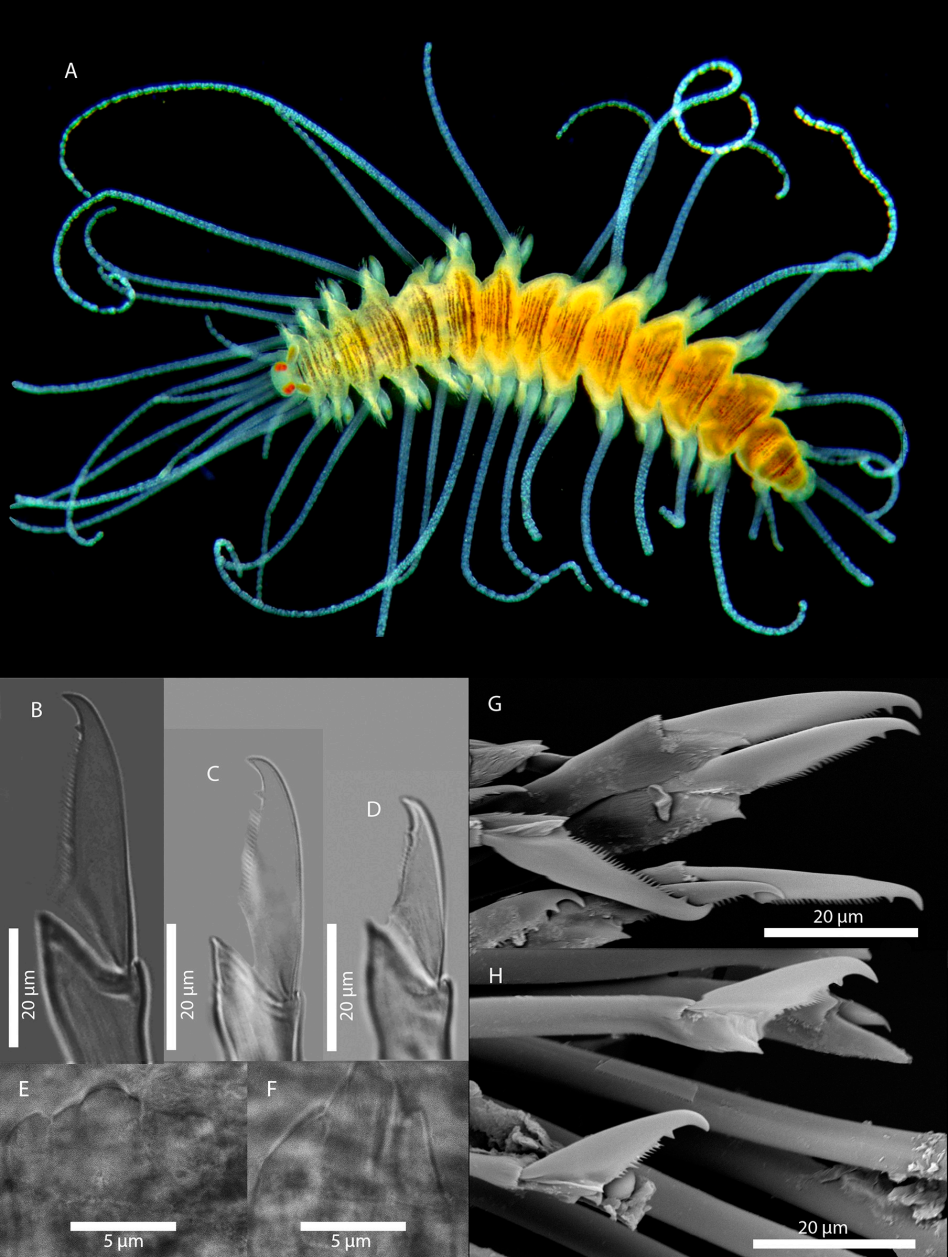
*Amblyosyllis anae* n. sp. (A) Live specimen, dorsal view, MNCN 16.01/18028. (B) Dorsal chaeta, midbody segment. (C) Medial chaeta, midbody segment. (D) Ventral chaeta, midbody segment. (E, F) Trepan teeth, cusps difficult to discern. (G) Dorsal chaetae, midbody segment, SEM. (H) Ventral chaetae, midbody segment, SEM.

*Amblyosyllis speciosa* Pernet, 1998 [23]: 1369–1371, Fig 1; not Izuka, 1912 [3].

**Material examined:** Holotype (MNCN 16.01/18029), and paratypes (MNCN 16.01/18028, 30, 31, 18463, 18467).

**Type Locality:** Friday Harbor Laboratory, Washington State, USA (North Pacific Ocean).

**Habitat:** Between 0–75 m; epifauna on floating dock, barnacles with associated fauna, sponges, hydroids, and *Polycarpa*.

**Distribution:** Only known from the type locality.

**Description:** Holotype incomplete, with five anterior chaetigers, 1.03 mm long, 0.6 mm wide. With variable number of transversal dark lines per segment (Fig 25A). Nuchal lappets reaching end of first chaetiger. Prechaetal digitiform papilla short and triangular; ventral cirri as long as parapodial lobes, digitiform and wide, covered by secretions in paratype MNCN 16.01/18028. Compound chaetae short and bidentate (Fig 25B, 25C, 25G and 25H), around 12–13 in midbody parapodia. Distal tooth considerably larger than proximal one (Fig 25B, 25C, 25G and 25H). Trepan with six pentacuspid teeth (Fig 25E and 25F).

**Variation:** Dorsum covered by cilia in one paratype (MNCN 16.01/18028).

**Remarks:** Chaetae similar in shape to those of *A*. *hectori* n. sp. and *A*. *speciosa*. However, they are shorter and the colour pattern is different. Pernet [23] cultured one species of *Amblyosyllis* also from Friday Harbor (Washington) and documented its brooding mode. The species was identified as *A*. *speciosa*. However, we have been able to check one colour picture of the specimens studied by Pernet (kindly sent by the author) and its colour pattern, including the blueish light reflection in the dorsal cirri when seen through optical microscopy, matches perfectly with *A*. *anae* n. sp. (Fig 25A). Hence, the brooding mode documented by Pernet [23] should be assigned to this species.

**Etymology:** This species is dedicated to Ana Bleidorn Aguado, who was born when the final version of this manuscript was ready. The yellow ground colour of this species (as seen in Fig 25A) resembles the hair colour of Ana.

#### Lineage 16

***Amblyosyllis nigrolineata* Okada**, **1934**

Fig 26

**Fig 26:**
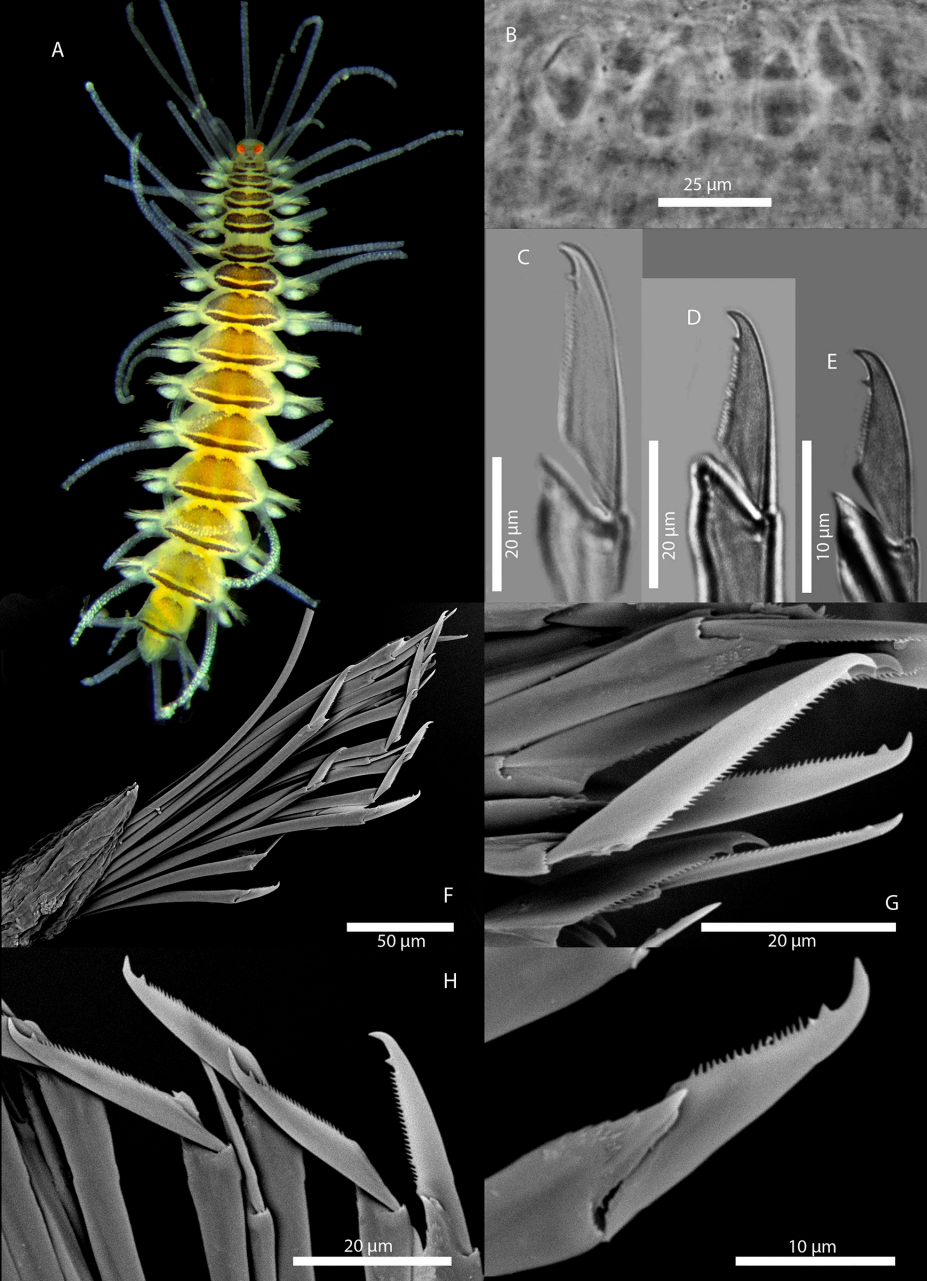
*Amblyosyllis nigrolineata*. (A) Live specimen, dorsal view, MNCN 16.01/18041. (B) Trepan. (C) Dorsal chaeta, midbody segment. (D) Medial chaeta, midbody segment. (E) Ventral chaeta, midbody segment. (F) Chaetal fascicle, midbody segment, SEM. (G, H) Medial chaetae, midbody segment, SEM. (I) Ventral chaeta, midbody segment, SEM.

*Amblyosyllis nigrolineata* Okada, 1934 [82]: 317–320, Figs 1, 2.

*Amblyosyllis speciosa* Aguado *et al*., 2008 [139]: 522, 527, Figs 1, 2; not Izuka, 1912 [3].

**Type locality**. Misaki, Japan (West Pacific Ocean).

**Material examined:** 11 specimens (MNCN 16.01/18032, 33, 35–43, 18468, 18469).

**Locality of material examined:** San Diego and Los Angeles, California, USA (North Pacific Ocean).

**Habitat:** Between 0–1 m; epifauna on floating docks and impounded ships, and intertidal flat with surfgrass (*Phyllospadix* sp).

**Distribution:** Japan, Australia and California (Pacific Ocean).

**Description of material examined:** Best preserved specimen (MNCN 16.01/18032) incomplete, with eight anterior chaetigers, 4.9 mm long, 1.43 mm wide. With a big patch of dark pigmentation that occupies three quarters of each segment, and a dark thick transversal line in the posterior-most margin, being both areas separated by a transversal colourless line (Fig 26A). Two ciliated nuchal lappets, extending over first chaetiger. Dorsum covered by bunches of cilia. Digitiform papilla longer in posterior chaetigers; ventral cirri as long as parapodial lobes, with round and wide shape. Parapodia with up to 16–18 short bidentate chaetae on midbody parapodia (Fig 26C–26I). Distal tooth considerably larger than proximal one (Fig 26C–26I). Trepan with six teeth (Fig 26B), probably pentacuspid, though difficult to discern in the material examined.

**Remarks:** The specimen MNCN 16.01/18040 is dorsally covered by symbiont protists similar to those found on specimens of *A*. *emilioi* n. sp. Our 16S sequences from *A*. *nigrolineata* were blasted in GenBank obtaining 98–100% of identity with the species *A*. *speciosa* (JF903685) from Japan and *A*. cf. *speciosa* (JF903682) from Australia [9, 139]. The colour pattern and chaetae shape coincide with *A*. *nigrolineata*, from Japan [82], which was considered to be close to *A*. *speciosa* by Hartman [30], and finally synonymised by Imajima & Hartman [11]. We reinstate *A*. *nigrolineata* since the chaetal shape and colour pattern is very distinct and differs from the one originally described for *A*. *speciosa* by Izuka [3]. Accordingly, the species identification of the specimens from GenBank (JF903685 and JF903682) has been also changed. The distribution of *A*. *nigrolineata* at both sides of the Pacific (Japan, Australia and California) is confirmed herein by morphological and molecular data.

Both, *A*. *speciosa*, and *A*. *nigrolineata*, share the same type locality, close to Misaki Marine Biological Station (Sagami Province, Japan) and, in both cases, the type material has not been located and may be lost (e.g. [143–145]).

In spite of the fact that the first reports of *A*. *speciosa* in the Pacific coasts of the US [23, 142] referred to two different native species (namely *A*. *hectori* n. sp. and *A*. *anae* n. sp., described as new in the present work), the species (under the name of “*Amblyosyllis speciosa form 4*” [146]) is presently considered as introduced. However, this morphotype corresponds to *A*. *nigrolineata*, as confirmed by comparing most pictures of the allegedly introduced specimens available online with our Fig 26A. The possible introduction vector has never been identified or even suggested.

#### Lineage 17

***Amblyosyllis plectorhyncha* (Marenzeller**, **1874**)

Fig 27

**Fig 27:**
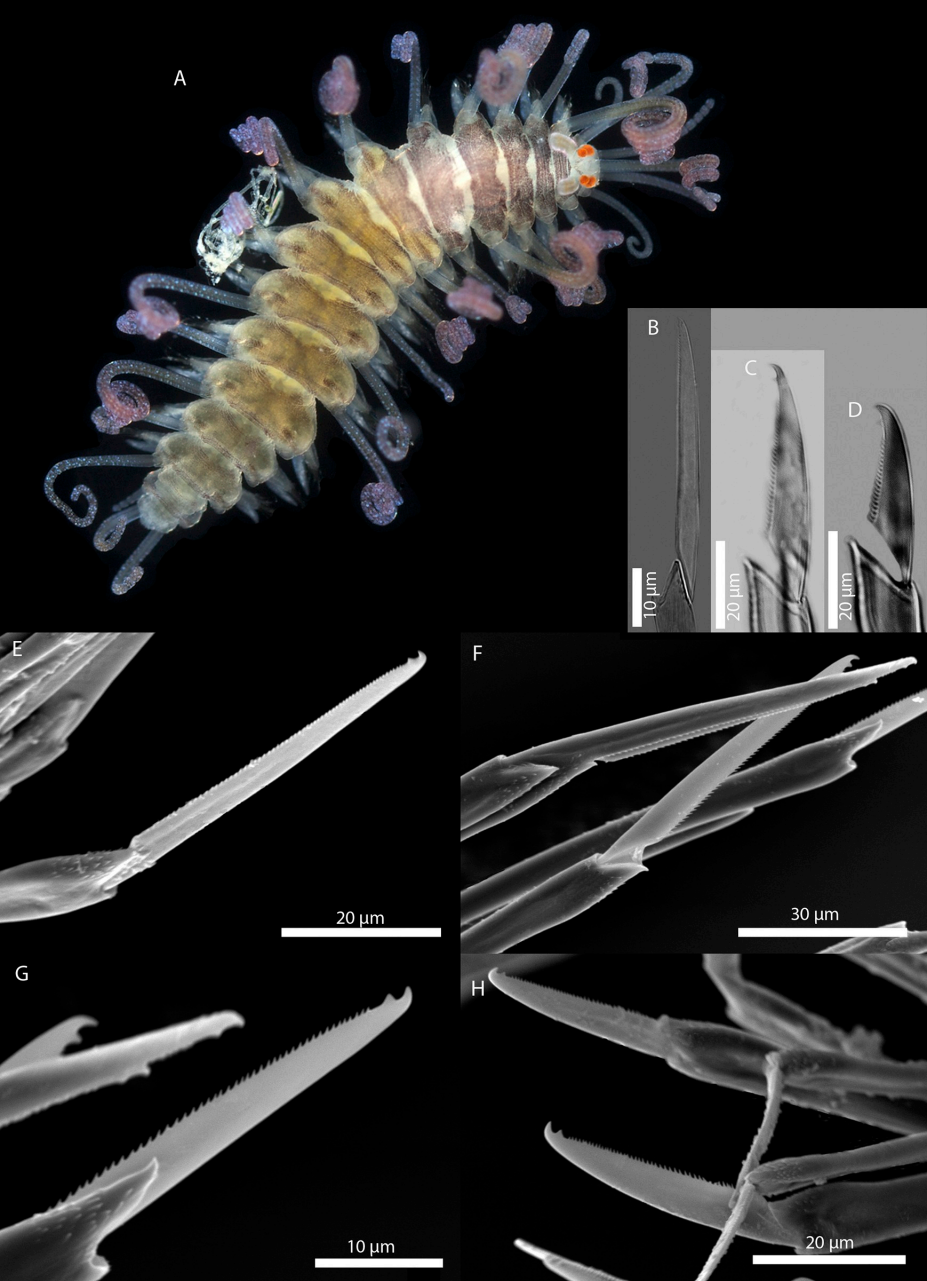
*Amblyosyllis plectorhyncha*. (A) Live specimen, dorsal view, MNCN 16.01/18044. (B) Dorsal chaeta, midbody segment. (C) Medial chaeta, midbody segment. (D) Ventral chaeta, midbody segment. (E) Dorsal chaeta, anterior segment, SEM. (F) Dorsal chaetae, midbody segment, SEM. (G, H) Medial chaetae, midbody segment, SEM.

*Pterosyllis plectorhyncha* Marenzeller, 1874 [81]: 47–50, plate V, Fig 3.

**Type locality:** Muggia, Italy (Adriatic Sea)

**Material examined:** One specimen (MNCN 16.01/18044).

**Locality of material examined:** Istria, Croatia (Adriatic Sea).

**Habitat:** 27 m; amongst shells, ascidians and sponges.

**Distribution:** Adriatic Sea.

**Description:** One single preserved specimen, incomplete, with nine chaetigers, 2.8 mm long, 0.9 mm wide. Dorsal colour pattern consisting of one broad transversal line per segment with an anterior constriction in the middle region, occasionally darker in the lateral areas of each segment. Dorsal cirri with purple colouration (Fig 27A). Two elongated nuchal lappets, extending over first chaetiger and a small anterior portion of the second one. Ventral cirri longer than parapodial lobes, spindle shaped. Parapodia with around 15–20 long, bidentate chaetae (Fig 27B–27H). Distal tooth larger than proximal one. Trepan not observed.

**Remarks:** The single available specimen perfectly coincided with the description of the holotype, collected at Muggia (Italy, Adriatic Sea), at less than 100 km far from Istria (Croatia, Adriatic Sea), where our specimen was found. This way, *A*. *plectorhyncha*, previously synonymised with *A*. *formosa*, is here considered to be valid and the species is reinstated. Chaetae of *A*. *plectorhyncha* are similar to those of *A*. *idae* n. sp. and *A*. *spectabilis*, but clearly differs in its colour pattern.

**Etymology:** The specific epithet *plectorhyncha* is composed by the combining forms *plecto*-, from the Greek *plektos* and meaning ‘twisted’ or ‘interwoven’, and -*rhyncha*, from the Greek *rhynchos* and meaning ‘snout’ or ‘proboscis. It refers to the fact that the proboscis and pharynx are so folded and twisted that it is difficult to unravel one from each other [81]: “Die Rüssel–und Schlundröhre sind ausserordentlich lang. Schon die erstere ist gefaltet und gewunden und die letztere so vielfach ineinander geschlungen, dass es fast unmöglich scheint, diesen Knäuel zu entwirren” (= “The proboscis and the pharyngeal tubes are extremely long. The former is so folded and twisted, and the latter so interlocked with it that it seems almost impossible to unravel this ball”).

#### Lineage 18

***Amblyosyllis idae* n. sp.**

Figs 28 and 29

**Fig 28:**
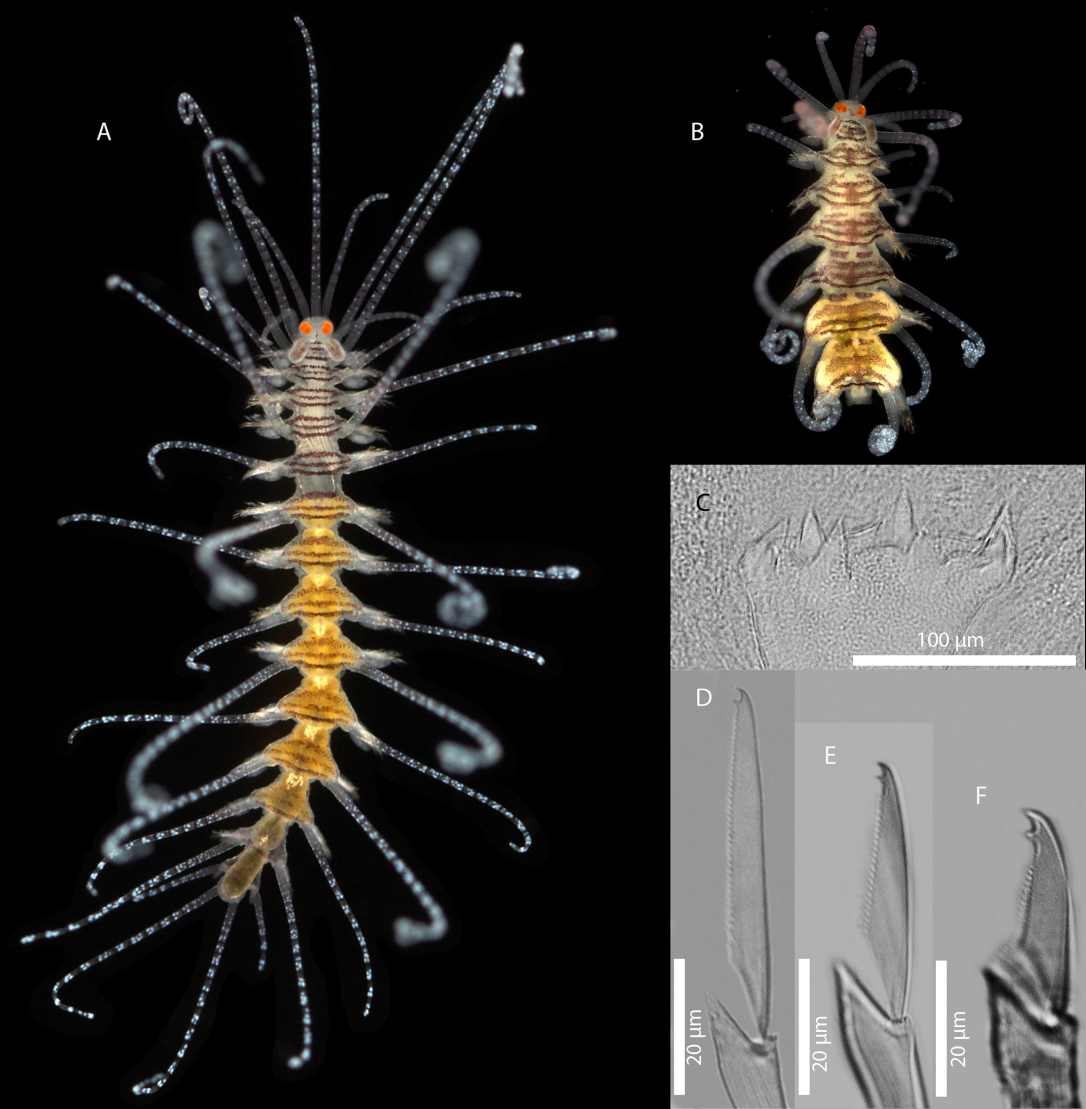
*Amblyosyllis idae* n. sp. (A–B) Live specimens with differences in colour patterns, dorsal view; A: MNCN 16.01/18050, B: MNCN 16.01/18049. (C) Trepan. (D) Dorsal chaeta, midbody segment. (E) Medial chaeta, midbody segment. (F) Ventral chaeta, midbody segment.

**Fig 29:**
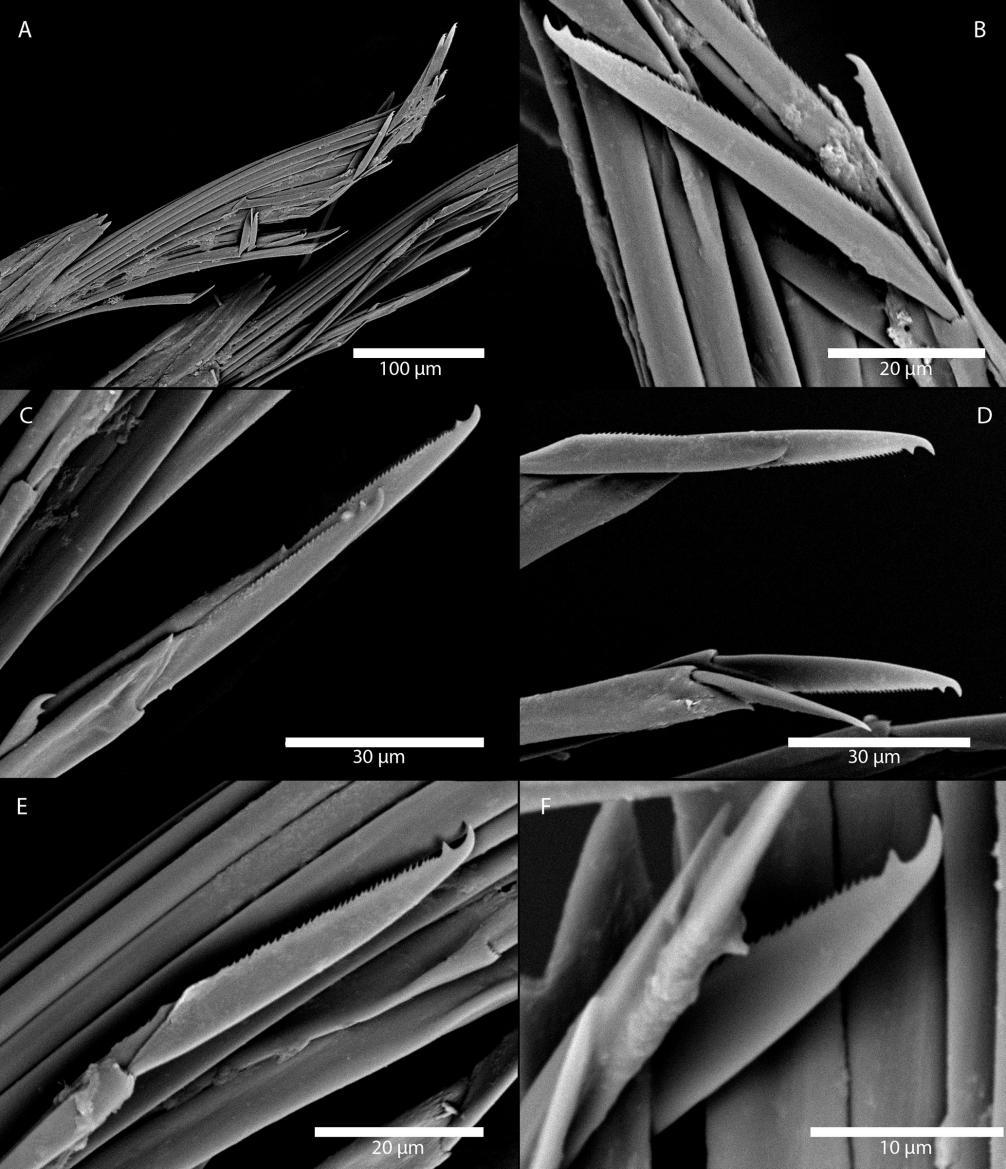
*Amblyosyllis idae* n. sp. SEM. (A) Midbody fascicle, ventral view. (B) Dorsal chaeta, midbody segment. (C–E) Medial chaetae, midbody segments. (F) Ventral chaeta, midbody segment.

**Material examined:** Holotype (MNCN 16.01/18050), three paratypes (MNCN 16.01/18047–49), and two additional specimens (MNCN 16.01/18045–46).

**Type locality:** Capri, Italy (Tyrrhenian Sea)

**Locality of material examined:** Banyuls-sur-Mer, France (Mediterranean Sea); Capri and Ischia, Italy (Tyrrhenian Sea).

**Habitat:** Between 0–15 m; among mussels with epifauna, growing on floating jetty, coralline and well sorted sand.

**Description:** Holotype incomplete, with 12 segments, 2.3 mm long, 0.5 mm wide. Three transversal coloured lines per segment (Fig 28A and 28B). Two nuchal lappets extending over first chaetiger with dark colour inside (Fig 28A and 28B). Dorsal cirri with secretions. Long, bidentate chaetae (Figs 28D–28F and 29A–29F), around 12–15 on midbody parapodia. Distal teeth slightly larger than proximal one or approximately equivalent in size. Trepan with six pentacuspid teeth (Fig 28C), lateral cusps difficult to discern.

**Variation**: Colour pattern of Italian specimens consists of three transversal lines per segment (Fig 28A), while the specimens from Banyuls may show a more complex pattern (Fig 28B). In both locations were also found whitish specimens with weak coloration.

**Fig 30:**
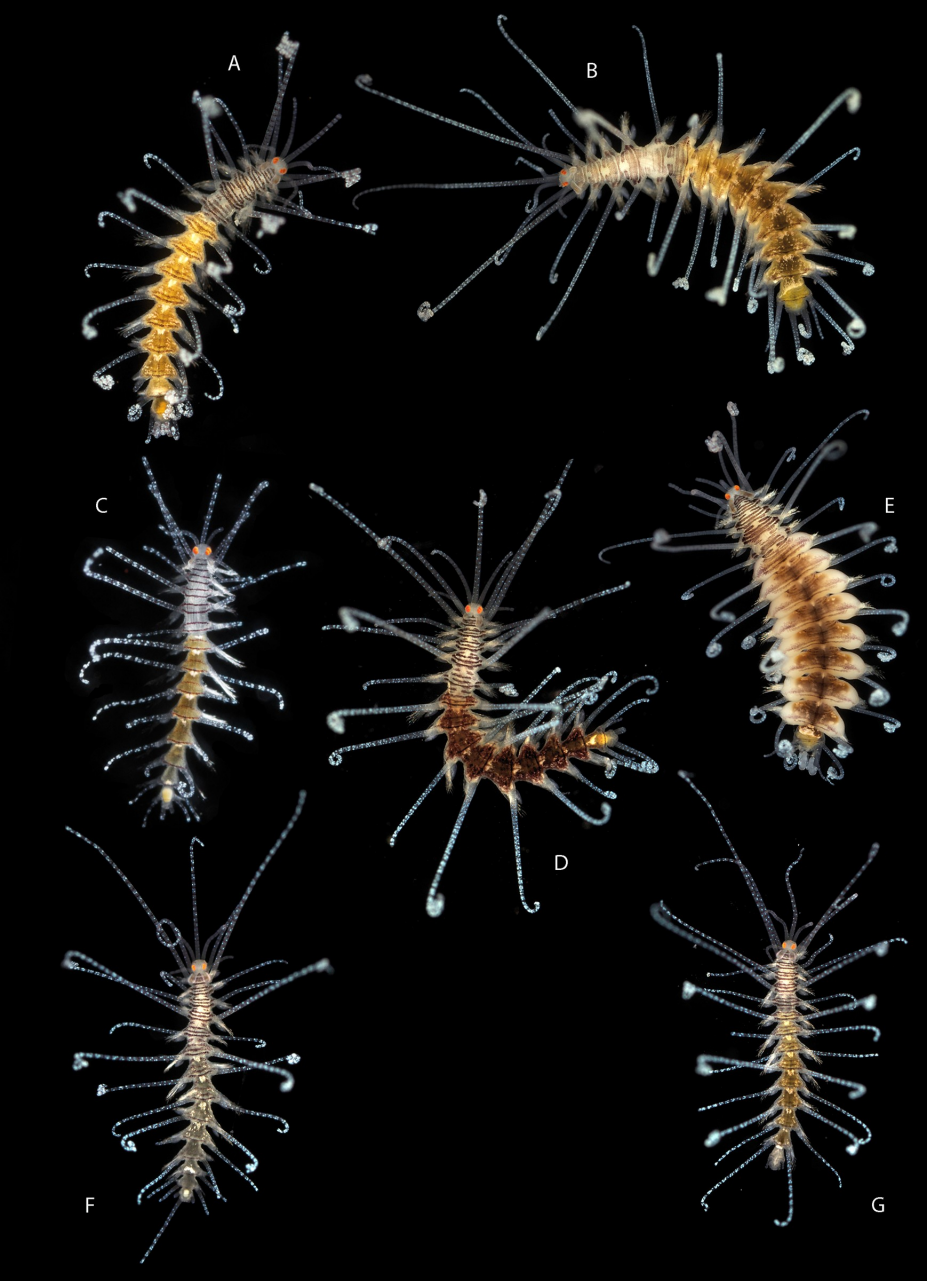
Amblyosyllis spectabilis. (A–G) Live specimens with differences in the colour patterns, dorsal view; A: MNCN 16.01/18065, B: MNCN 16.01/18075, C: MNCN 16.01/18061, D: MNCN 16.01/18076, E: MNCN 16.01/18064, F: MNCN 16.01/18073, G: MNCN 16.01/18074.

**Fig 31:**
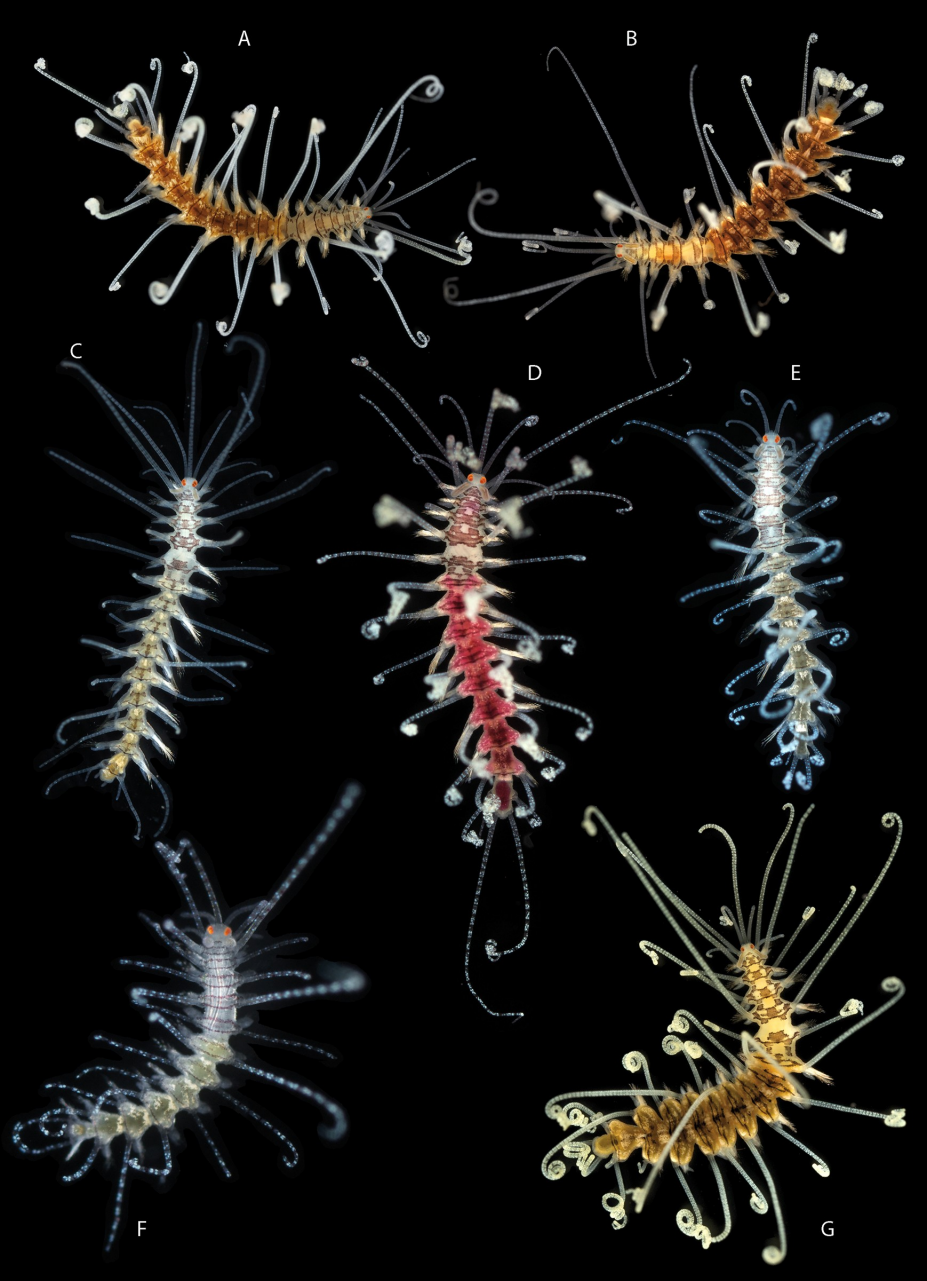
Amblyosyllis spectabilis. (A–G) Live specimens with differences in the colour patterns, dorsal view; A: MNCN 16.01/18080, B: MNCN 16.01/18052, C: MNCN 16.01/18059, D: MNCN 16.01/18060, E: MNCN 16.01/18058, F: MNCN 16.01/18057, G: MNCN 16.01/18077.

**Remarks:** Chaetae similar to those of *A*. *plectorhyncha* but the colour pattern is different. In some specimens (Fig 28A), it is similar to that attributed to *A*. *lineolata* (Fig 1C), a *nomem dubium* species from the Gulf of Naples. However, *A*. *lineolata* had two clear and continuous transversal lines per segment and one dotted line in the middle; while *A*. *idae* n. sp. shows three clearly continuous transversal lines per segment. Such colour pattern also resembles that of some *A*. *spectabilis* (Figs 30C, 30D, 31E and 31F), but differs in having chaetae with smaller distal tooth. The picture of “*A*. *formosa*” from Porto Cesareo, Lecce [147] by Fabio Vitale, shows the same colour pattern of *A*. *idae* n. sp. and probably represents the same species.

**Etymology:** This species is dedicated to Arne Nygren’s daughter Ida Nygren.

#### Lineage 19

***Amblyosyllis spectabilis* (Johnston *in* Baird**, **1861**)

Figs 30–33

**Fig 32:**
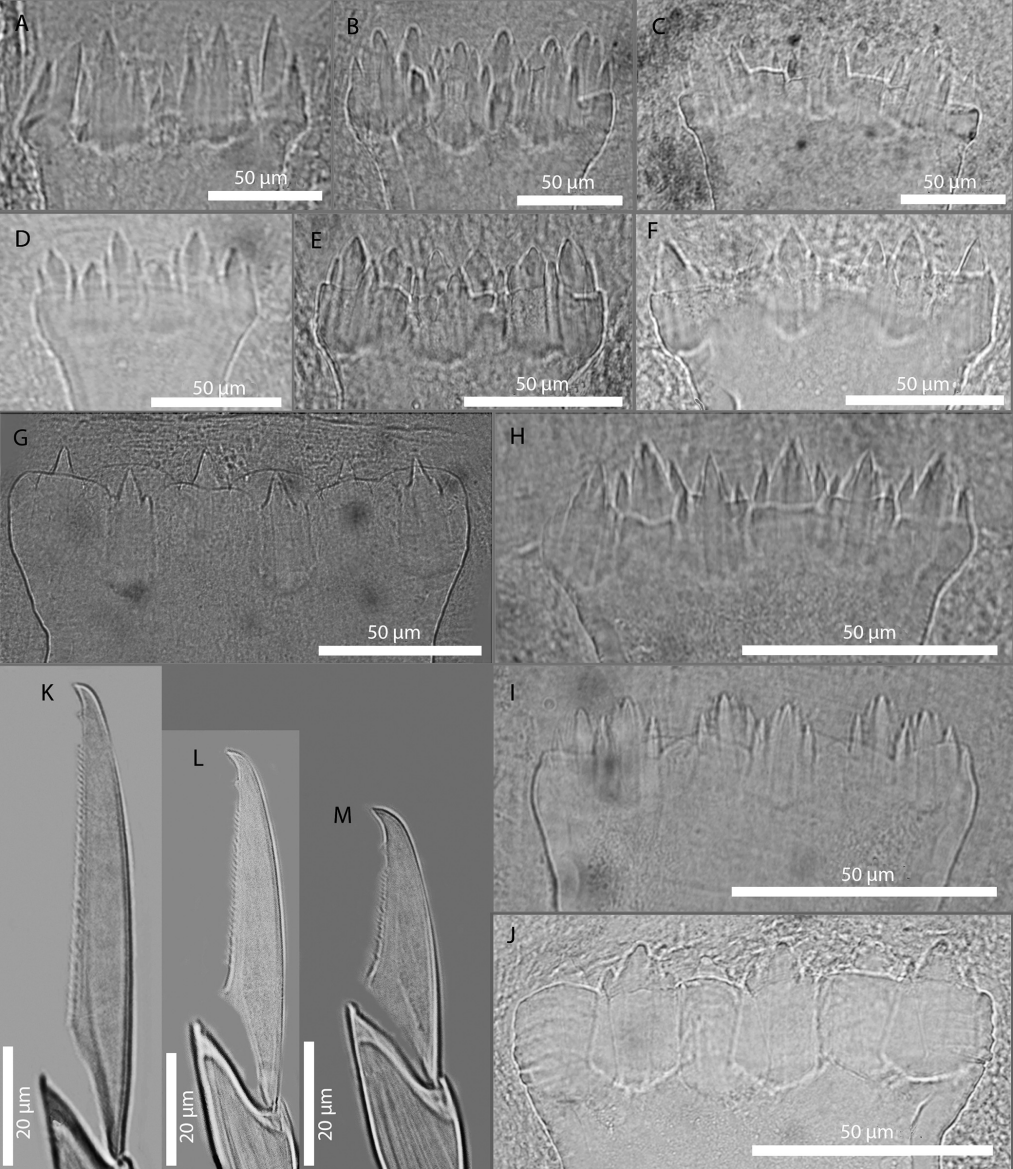
Amblyosyllis spectabilis. (A–J) Trepans of different individuals. (K) Dorsal chaeta, midbody segment. (L) Medial chaeta, midbody segment. (M) Ventral chaeta, midbody segment.

**Fig 33:**
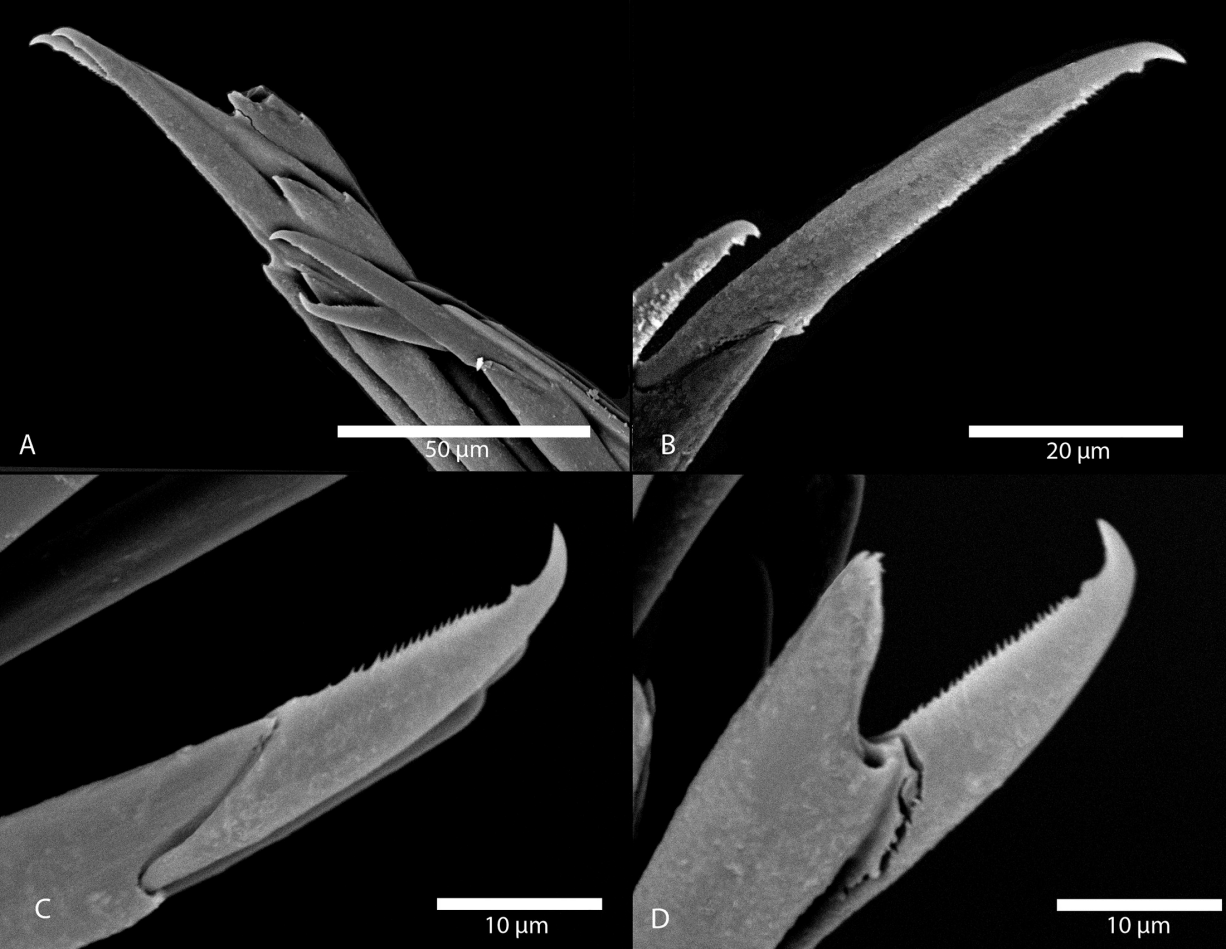
*Amblyosyllis spectabilis*. SEM. (A) Dorsal and medial chaetae, midbody segment. (B–C) Dorsal and medial chaetae, midbody segment. (D) Ventral chaeta, midbody segment.

*Gattiola spectabilis* Johnston *in* Baird, 1861 [80]: 298 (footnote); Johnston, 1865 [1]: 195–196, plate XVIa, Figs 1–7.

*Pterosyllis formosa* Claparède, 1863 [4]: 46, plate XIII, Figs 30–34.

*Pterosyllis dorsigera* Claparède, 1864 [5]: 560–562, plate VIII, Fig 1.

? *Nicotia lineolata* Costa, 1864 [6]: 160–164, plate III, Figs 1–14.

*Thylaciphorus hessii* Quatrefages, 1865 [108]: 55–56.

*Ambyosyllis immatura* Langerhans, 1879 [29]: 561, plate XXXII, Fig 20.

*Amblyosyllis formosa* San Martín, 2003 [18]: 93–96, Figs 39–40.

**Type locality:** The species was described based on material from several localities (Cullercoats, North Sea; Fowey, English Channel; Infracombe, Bristol Channel; and Tenby, Celtic Sea); no types designated.

**Material examined:** 29 specimens **(**MNCN 16.01/18051–80, 18470–73, 76, 78).

**Additional examined material.** MNCN 16.01/6535, one specimen, Nerja, Málaga, Spain (Mediterranean Sea). MNCN 16.01/14005, one specimen, Ría de Ferrol, Galicia, Spain (North Atlantic Ocean). MNCN 16.01/6529, one specimen, Ibiza, Baleares, Spain (Mediterranean Sea), MNCN 16.01/18084 and MNCN 16.01/18085, two specimens, Plymouth, Great Britain (North Atlantic Ocean)

**Locality of material examined:** Cádiz, Spain (Atlantic Ocean); Banyuls-sur-Mer, France (Mediterranean Sea); Istria, Croatia (Adriatic Sea); Ischia, Italy (Tyrrhenian Sea); Funchal, Caniçal, and Porto Moniz, Madeira Island (Atlantic Ocean); Plymouth, Great Britain (North Atlantic Ocean).

**Habitat:** Intertidal; on floating docks, hydroids, bryozoans (*Bugula*), sponges, coralline, mussels with epifauna, lime rock with epifauna, algae, shells, ascidians, sand, and shell gravel.

**Distribution:** This species has been considered cosmopolitan (Table 2). However, we recommend checking identifications with molecular information. The species is confirmed in the Northeastern Atlantic Ocean: North Sea, Celtic Sea, English Channel, Madeira Island, Gulf of Cádiz, Mediterranean Sea (France, Italy), and Adriatic Sea (Croatia).

**Description of material examined:** Best preserved complete specimen (MNCN 16.01/18085), 5.1 mm long, 1.1 mm wide. Colour pattern very variable, often with several transversal lines per segment and occasionally dark spots in the anterior-most dorsal region of each chaetiger with variable pattern, some specimens with the shape of a ∞ (Figs 30A–30G and 31A–31G). Two elongated nuchal lappets extending over first chaetiger. Medium length, bidentate chaetae (Figs 32K–32M and 33A–33D), around 22–28 on midbody parapodia. Distal tooth considerably larger than proximal one (Figs 32K–32M and 33A–33D). Trepan formed by six pentacuspid teeth (Fig 32A–32J).

**Remarks:** Colour pattern variable (the specimen in Fig 30D shows a pink colour probably because it fed on the red algae where it was found), but chaetal shape and size quite stable. Shape of cusps in trepan’s teeth appearing to be variable (Fig 32A–32J) since the lateral cusps may occasionally be difficult to discern (Fig 32D and 32G). However, they are always pentacuspid. Several specimens were collected from Plymouth, close to Fowey (Cornwall, British Channel), one of the type localities of the species. COI sequences of *A*. *spectabilis* from this study match with more than 98% of identity with specimens previously identified as *A*. *formosa* (EF123745) from Banyuls-sur-Mer, France [8].

*Gattiola spectabilis* has been considered a junior synonym of *Pterosyllis formosa* Claparède, 1863 [4], described from the same geographic area. However, and as stated in the genus remarks, the species was originally published by Baird [80], as *G*. *spectabilis* Johnston, and not by Johnston [1], as has been normally considered with a few exceptions by early authors. This way *G*. *spectabilis* predates *P*. *formosa* and its synonymies, and should be referred to as *A*. *spectabilis*.

**Etymology:** The specific epithet of *Gattiola spectabilis* is a Latin adjective meaning ‘remarkable, ‘admirable’ or ‘worth seeing’, and seems to refer to the general features of the species, stated to be a “remarkable form” [80], being “very beautiful creatures” (Hancock *in* Johnston [1]), with their segments being “marked prettily with fucous lines” [1], and the dorsal cirri “curled in a beautiful spiral” (Alder *in* Johnston [1]).

The specific name of *Pterosyllis formosa* is a Latin adjective meaning ‘beautiful’, and refers to the beauty of the species, referred by Claparède [4] as “Von dieser niedlichen Art traf ich nur ein einziges, aus 16 Gliedern bestehendes Individuum” (translated roughly as “Of this cute species I met only a single, 16-segmented individual”).

The specific name of *Pterosyllis dorsigera*, is composed by the Latin prefix *dorso*-, meaning ‘dorsal’ or ‘back’, and the Latin suffix -*ger*, used to form adjectives meaning ‘bearing’ or ‘carrying’ from nouns, being this way ‘back carrier’. The name refers probably to the characteristic dorsal colour pattern on each of the anterior segments of the species, in the shape of a ∞ with each half with slightly hexagonal outlines.

*Thylaciphorus hessii* is named after Charles-Eugène Hesse (1801–1890), Breton naturalist particularly interested in the marine fauna of the region of Brest (France), who found the type material at the harbour of Brest and provided the author with a drawing of a living specimen [108].

The specific epithet of *Amblyosyllis immatura* is a Latin adjective meaning ‘immature’ or ‘unripe’, and refers to the immature condition of the type species, described as being “Ein unreifes Exemplar” (“An immature specimen”) [29].

## Discussion

The monophyly of *Amblyosyllis* has been confirmed herein based on DNA data, in agreement with previous analyses [8, 9], as well as by morphological homogeneity (i.e., trapezoidal shape of segments, presence of an achaetous prepygidial segment and 13 fixed chaetigers [15]). The reasons for these fixed morphological characteristics are unidentified, and difficult to discern. This, together with the fact that little is known about the biological cycle, ecology, habits, diet, etc., of members of the genus, makes this animal interesting for research.

*Amblyosyllis*, however, has not received enough attention during the last decades [24]. Most of the species descriptions are quite old and lack detailed iconography. Additionally, most type series (including the type species, *A*. *rhombeata*) never existed or are considered to be lost. Consequently, redescriptions are problematic and the process of identification difficult. Up to date, there were 22 nominal species and subspecies (including synonymies and species names with dubious validity–*nomina dubia*) (Table 1). Fifteen were described between 1857 and 1897, five more during the 20th century, and only two at the beginning of the 21st century; none of them with the support of molecular data (Table 1). Most of the descriptions were made by some of the earliest European zoologists (e.g. [2, 4, 5, 29, 39, 80] (Table 1). However, they are either inadequate according to present standards, or very brief and incomplete. For decades there was a particular interest in describing species from worldwide geographic areas (Australia, North and South America, Africa, etc.), many of them being conservatively identified using European bibliography and, thus, referred to the species names therein (e.g. [44, 47, 107, 148–150]. Moreover, many synonymies were stated (e.g. [30]), sometimes based on peculiar interpretations of the Principle of Priority (see for instance [151]). As a result, many European species ended with wider or even cosmopolitan distributions (i.e. the former *A*. *formosa*, now *A*. *spectabilis*,). During the recent decades, fresh materials have been examined in much more detail and with new techniques, leading to questioning some of these wide distributions that became restricted (e.g. *A*. *granosa*, by [40]). However, the problem persists, as in many other polychaete groups [152].

In our study, we have combined for the first time several methodologies and sources of data. The results enable us to delineate *Amblyosyllis* species within our sample. However, there are still many geographical areas and habitats that have not been covered, and new information necessary to revise the whole group is still needed. We found 19 species in a pool of 115 specimens, from a total of 16 bioregions (Fig 2). The species are overall morphologically similar (showing several constant features and few interspecific variations). However, they are genetically very diverse. Interspecific COI divergence marker is always higher than 6%, and, in most cases, between 18–28%, thus greater than the barcoding gap accounted for most groups of organisms [153, 154]. In annelids, intraspecific and interspecific distances are 3.56%, and 20.06% on average, respectively [155]. Yet, COI intraspecific distances in some species complexes may range from 0–3%; e.g., [61, 156–158] to higher than 5% (e.g., [60, 62, 159]). 16S sequences lead to interspecific distances of 10–28%, which are averall higher than those obtained for other polychaete groups [160, 161], except between lineages 9–10, and 11–12 (where sequences differ in less than 3%, see Results and S3–S5 Files). For 28S sequences, differences between accepted species are larger than 4%, except for lineages 17–18–19 (1%) and 13–14–15 (2–3%) (S3–S5 Files), which could still be considered as significant since this marker is a slow evolving gene.

Statistical parsimony analyses of haplotypes (Fig 5) and mPTP (S6 File) over-split clades into networks and clusters, respectively, when analysing the three marker partitions separately. This is explained by both the low number of specimens representing some groups (in several cases only two) and by the presence of several “singletons¨. The mPTP developers suggest removing singletons since “the lack of multiple sequences per species will inevitably decrease the distinctiveness of the two evolutionary processes and consequently the identification of the shifting point from one to the other” [101]. Similar problems have been found in previous studies [104]. However, the mPTP analyses of combined COI, 16S and 28S showed more congruent results recovering 21 clusters, mostly matching the 19 putative species after analyses of morphological data and phylogenetic analyses of the combined datasets. The only exception were lineages 6 and 9 (*A*. *ovei* n. sp. and *A*. *clarae* n. sp.), which were subdivided into two after mPTP analyses of concatenated datasets. Our taxonomic proposal, (i.e. 19 species) could be considered conservative and that further work should be done to establish if members of these two clades should be sub-divided.

The representation of certain lineages by a low number of terminals does not allow for population structure studies. However, the larger Mediterranean groups (see for instance 30 terminals in *A*. *spectabilis*) allows detecting structuring processes as possible early stages of speciation [162]. Except for *A*. *finmarchica* and *A*. *nigrolineata*, most species seem to have restricted distributions, which may suggest there are fewer examples of widely distributed species than previously thought. Additionally, the species of *Amblyosyllis* may have reduced dispersal abilities, since they probably lack planktotrophic larvae, as demonstrated for the Washington population of *A*. *anae* n. sp. [23].

Within the analyzed sample, morphospecies C and G are divided in two by the complementary methods: lineages 3–4 (*Amblyosyllis* sp. 2 and *Amblyosyllis* sp. 3), and 8–10 (*A*. *madeirensis* and *A*. *lineata*), respectively (Figs 5 and 6). They represent examples of morphologically cryptic species. In these cases, cryptic species are temporary delineations (morphospecies hypothesis to be tested by molecular methods), rather than natural phenomena, as proposed by Struck *et al*. [56]. Regardless their morphological similarity and sympatric distribution *A*. *madeirensis* and *A*. *lineata* are not sister taxa (S1 File) most likely after retaining the plesiomophic features from a common ancestor (together with *Amblyosyllis* sp. 4 and *A*. *clarae* n. sp.). *Amblyosyllis clarae* n. sp., however, is a deeper occurring species (150–200 m), from northern Europe, while *Amblyosyllis* sp. 4 is a species from Hong Kong, with different morphology. Additionally, the separation of these morphospecies by other methods allowed us to recognize some morphological variation that was not initially considered in detail. For instance, slight differences in the chaetal shape between *Amblyosyllis* sp. 2–*Amblyosyllis* sp. 3, and *A*. *madeirensis*–*A*. *lineata*, together with some variability in colour pattern of the latter doublet. Some authors treat these cases as examples of pseudocryptic species [53, 54].

Six species (*Amblyosyllis antoni* n. sp., *A*. *madeirensis*, *A*. *lineata*, *A*. *plectorhyncha*, *A*. *idae* n. sp. and *A*. *spectabilis*) are present in Mediterranean waters, while *A*. *madeirensis* and *A*. *spectabilis* occur also in the East Atlantic (Madeira). *Amblyosyllis ovei* n. sp. and *A*. *rhombeata* are from Florida and Belize; *A*. *emilioi* n. sp. from Chile; *A*. *hectori* n. sp., *A*. *anae* n. sp. and *A*. *nigrolineata* from California, and finally *Amblyoysllis* sp. 2 and *Amblyosyllis* sp. 4 from New Zealand (Fig 6). These species show overlapping distribution patterns, besides sharing habitat and bathymetric distribution. They are considered to be sympatric species.

## Conclusions

- Nineteen species of *Amblyosyllis* are identified after considering different sources of data and analytical methods.
- The genetic divergence in the genus is high, in contrast to the morphological homogeneity.
- The presence/absence of nuchal lappets, colour pattern, number of teeth in the trepan and chaetae shape have been found useful morphological characteristics for species identification. However, they should be combined with molecular methods.
- Two species, *A*. *finmarchica* and *A*. *nigrolineata*, present a wide geographical distribution, while the other species show a more restricted distribution.
- There are several examples of sympatric species from the Mediterranean, Florida and Belize, Chile, California and New Zealand that show overlapping distribution patterns, and share habitat and bathymetric distribution.
- A combined study of phenotypic disparity and genetic divergence is the best strategy for sharp species delineations.

## Supporting information

S1 File Occurrence and collection information of the especimens examined and sequenced. * Paratype, ** Holotype. Studied material, taxonomic identification and lineage correspondence, voucher number, GenBank accession number, geographic distribution, habitat and depth. Number of biogeographic provinces refer to (As per Spalding *et al*. 2017): 1, Arctic; 2, Northern European Seas; 3, Lusitanian; 4, Mediterranean Sea; 10, Cold Temperate Northeast Pacific; 11, Warm Temperate Northeast Pacific; 12, Tropical Northwestern Atlantic; 25, South China Sea; 45, Warm Temperate Southeastern Pacific; 54, Southern New Zealand; 56, Southeast Australian Shelf. Numbers for Ecoregions stand for: 18. North and East Barents Sea; 22. Southern Norway; 25. North Sea; 27. South European Atlantic Shelf; 29. Azores Canaries Madeira 30. Adriatic Sea; 35. Western Mediterranean; 57, Oregon, Washington, Vancouver Coast and Shelf; 59. Southern California Bight; 70. Floridian; 113. Southern China; 117. Sunda Shelf/Java Sea; 178. Araucanian; 199. Central New Zealand; 206. Western Bassian.
(DOCX)

S2 File Phylogenetic trees. (A) ML tree obtained from the analyses of the combined mitochondrial data set (COI+6S); Bootstrap support values below nodes. (B) Majority rule consensus tree from BI analysis obtained from the complete combined data set (COI+16S+28S); posterior probability support values close to each node.
(PDF)

S3 File Divergences measured in COI fragment using the Tamura 3-parameter model, with gamma distribution (= 1), shown in the lower left corner, and p-distance in the upper right corner. The analysis included 107 nucleotide sequences. Codon positions included were 1st+2nd+3rd+Noncoding. All ambiguous positions were removed for each sequence pair. There was a total of 624 positions in the final dataset (256 of which were parsimony informative). The number of base substitutions per site from averaging over all sequence pairs between clades are shown. Blue numbers indicate low distances (<0.1), between clades.
(DOCX)

S4 File Divergences measured in 16S fragment using the Kimura 2-parameter model, shown in the lower left corner, and p-distance in the upper right corner. The analysis included 55 nucleotide sequences. All ambiguous positions were removed for each sequence pair. There was a total of 364 positions in the final dataset (117 of which were parsimony informative). The number of base substitutions per site from averaging over all sequence pairs between clades are shown. Blue numbers indicate low distances (<0.1), between clades.
(DOCX)

S5 File Divergences measured in 28S fragment using the Tajima-Nei model, shown in the lower left corner, and p-distance in the upper right corner. The analysis included 98 nucleotide sequences. All ambiguous positions were removed for each sequence pair. There was a total of 348 positions in the final dataset (108 of which were parsimony informative). The number of base substitutions per site from averaging over all sequence pairs between clades are shown. Blue numbers indicate low distances (<0.02), between clades.
(DOCX)

S6 File Species delimitation analyses: Poisson Tree Process (PTP) and multi-rate Poisson Tree Process (mPTP). (A) PTP delimitation of COI sequences. Terminal branches in red indicate lineages that stand as separate species and the clades in red are lumped into single species. (B) mPTP delimitation of COI sequences. Terminal branches in red indicate lineages that stand as separate species and the clades in red are lumped into single species. C. mPTP delimitation complete combined data set (COI+16S+28S). Terminal branches in red indicate lineages that stand as separate species and the clades in red are lumped into single species.
(PDF)
