# Supplementary material for: Species delimitation in Amblyosyllis (Annelida, Syllidae)
Source: PLoS One. 2019 Apr 10;14(4):e0214211. doi: 10.1371/journal.pone.0214211 (PMC6457521; doi:10.1371/journal.pone.0214211)
Supplement: S1 File — * Paratype, ** Holotype. Studied material, taxonomic identification and lineage correspondence, voucher number, GenBank accession number, geographic distribution, habitat and depth. Number of biogeographic provinces refer to (As per Spalding et al. 2017): 1, Arctic; 2, Northern European Seas; 3, Lusitanian; 4, Mediterranean Sea; 10, Cold Temperate Northeast Pacific; 11, Warm Temperate Northeast Pacific; 12, Tropical Northwestern Atlantic; 25, South China Sea; 45, Warm Temperate Southeastern Pacific; 54, Southern New Zealand; 56, Southeast Australian Shelf. Numbers for Ecoregions stand for: 18. North and East Barents Sea; 22. Southern Norway; 25. North Sea; 27. South European Atlantic Shelf; 29. Azores Canaries Madeira 30. Adriatic Sea; 35. Western Mediterranean; 57, Oregon, Washington, Vancouver Coast and Shelf; 59. Southern California Bight; 70. Floridian; 113. Southern China; 117. Sunda Shelf/Java Sea; 178. Araucanian; 199. Central New Zealand; 206. Western Bassian. (DOCX) [file pone.0214211.s001.docx]

| **Species** | Voucher number | **Specimen code** | **Lineage** | **COI** | **16S** | **28S** | **Locality** | **Depth** | **Lat Start** | **Long Start** | **Habitat** | **Biogeographic realm** | **Province** | **Ecoregion** |
| --- | --- | --- | --- | --- | --- | --- | --- | --- | --- | --- | --- | --- | --- | --- |
| *A. antoni* n. sp. | MNCN16.01/18463* | 1103_BA | 1 | MH431295 | MH431090 | MH431188 | France, Banyuls | 25 | 42.499 | 3.141 | coralligenous | Temp N Atlantic | 4 | 35 |
|  | MNCN16.01/17972** | 1419_BA | 1 | MH431296 |  | MH431189 | France, Banyuls | 25 | 42.476667 | 3.161667 | coralligenous | Temp N Atlantic | 4 | 35 |
| *Amblyosyllis.* sp. 1 | MNCN 16.01/17973 | 1427_NZ | 2 | MH431285 |  | MH431180 | New Zealand, Kaikora | intertidal | −42.416667 | 173.666667 | rock pools with algae | Temperate Australasia | 54 | 199 |
|  | MNCN 16.01/17974 | 2598_NZ | 2 |  | MH431078 | MH431181 | New Zealand, Kaikora | intertidal | −42.416667 | 173.666667 | rock pools with algae | Temperate Australasia | 54 | 199 |
| *Amblyosyllis* sp. 2 | MNCN 16.01/17975 | 1429_NZ | 3 | MH431293 | MH431085 | MH431177 | New Zealand, Kaikora | intertidal | −42.416667 | 173.666667 | rock pools with algae | Temperate Australasia | 54 | 199 |
| *Amblyosyllis* sp. *3* | MNCN 16.01/17976 | 1466_OZ | 4 | MH431258 |  | MH431178 | South Australia, Gulf St. Vincent, Yorke Peninsula, Cowbowie Field Station, Edithburg jetty | 3-5 | −35.086194 | 137.747083 | mixed sand and gravel | Temperate Australasia | 56 | 206 |
|  | MNCN 16.01/17977 | 1467_OZ | 4 | MH431259 | MH431091 | MH431179 | South Australia, Gulf St. Vincent, Yorke Peninsula, Cowbowie Field Station, Edithburg jetty | 3-5 | −35.086194 | 137.747083 | mixed sand and gravel | Temperate Australasia | 56 | 206 |
| *A. finmarchica* | MNCN 16.01/17978 | 1477_SVA | 5 | MH431256 |  | MH431175 | Svalbard, Hinlopen | 52 | 79.447483 | 20.1397 | stones and gravel | Arctic | 1 | 18 |
|  | MNCN 16.01/17979 | 1478_SVA | 5 | MH431257 | MH431086 | MH431176 | Svalbard, Hinlopen | 52 | 79.447483 | 20.1397 | stones and gravel | Arctic | 1 | 18 |
| *A. ovei* n. sp. | MNCN 16.01/17980* | 2154_FL | 6 | MH431286 | MH431088 | MH431148 | USA, Florida Keys, Summerland Key, north point | intertidal | 24.686483 | −81.4439 | under bridge, *Caulerpa* | Tropical Atlantic | 12 | 70 |
|  | MNCN16.01/17981** | 2155_FL | 6 | MH431287 | MH431089 | MH431149 | USA, Florida Keys, Summerland Key, north point | intertidal | 24.686483 | −81.4439 | under bridge, *Caulerpa* | Tropical Atlantic | 12 | 70 |
|  | MNCN 16.01/18462* | 2156_FL | 6 | MH431288 |  | MH431150 | USA, Florida Keys, Summerland Key, north point | intertidal | 24.686483 | −81.4439 | under bridge, *Caulerpa* | Tropical Atlantic | 12 | 70 |
|  | MNCN 16.01/17983 | 1470_CBC | 6 | MH431289 |  | MH431151 | Belize, Carrie Bow Cay (Ellen Cay) | 10 | 16.803167 | -88.077817 | coarse sand | Tropical Atlantic | 12 | 68 |
|  | MNCN 16.01/17984 | 1471_CBC | 6 | MH431290 | MH431087 | MH431152 | Belize, Twin Cays, Lair Channel | 0.5 | 16.827917 | −88.100783 | among mangrove roots | Tropical Atlantic | 12 | 68 |
|  | MNCN 16.01/17985 | 1472_CBC | 6 | MH431291 |  | MH431153 | Belize, Pelican Cays, Manatee Cay | 1 | 16.666733 | −88.1922 | mangrove roots | Tropical Atlantic | 12 | 68 |
|  | MNCN 16.01/17986 | 1473_CBC | 6 | MH431292 |  | MH431154 | Belize, Pelican Cays, Manatee Cay | 1 | 16.666733 | −88.1922 | mangrove roots | Tropical Atlantic | 12 | 68 |
| *Amblyosyllis.* sp. 4 | MNCN 16.01/17987 | 0091_HK | 7 | MH431294 | MH431077 | MH431174 | Hong Kong, Cape D'Aquilar, Lobster bay | 1 | 22.206667 | 114.258333 |  | Central Indo-Pacific | 25 | 113 |
| *A. madeirensis* | MNCN 16.01/18462 | 0253_BA | 8 | MH431279 |  | MH431182 | France, Banyuls | 59-62 | 42.494167 | 3.1695 | mixed sample of hard and soft substrate. Tunicates and shells with epifauna, hydroids, sponges. | Temp N Atlantic | 4 | 35 |
|  | MNCN 16.01/17989 | 0983_BA | 8 | MH431280 |  | MH431183 | France, Banyuls | 25 | 42.499 | 3.141 | coralligenous | Temp N Atlantic | 4 | 35 |
|  | MNCN 16.01/17990 | 1077_CR | 8 | MH431281 |  | MH431184 | Croatia, Istria, off Rovinj | 15-27 | 45.074065 | 13.611099 | coralligenous | Temp N Atlantic | 4 | 30 |
|  | MNCN 16.01/17991 | 1081_CR | 8 | MH431282 |  | MH431185 | Croatia, Istria, off Rovinj | 27 | 45.087 | 13.608617 | shells, ascidians, sponges etc. | Temp N Atlantic | 4 | 30 |
|  | MNCN 16.01/17992 | 1461_MA | 8 | MH431283 | MH431084 | MH431186 | NW Madeira, Porto Moniz | 16-19 | 32.867817 | −17.163967 | rocks and sand | Temp N Atlantic | 3 | 29 |
|  | MNCN 16.01/17993 | 1462_MA | 8 | MH431284 |  | MH431187 | NW Madeira, Porto Moniz | 16-19 | 32.867817 | −17.163967 | rocks and sand | Temp N Atlantic | 3 | 29 |
| *A. clarae* n. sp. | MNCN 16.01/17994* | 1430_TRO | 9 | MH431260 | MH431079 | MH431155 | Norway, Trondheimsfjorden, Rødberg | 180-250 | 63.468217 | 9.999833 | *Lophelia* | Temp N Atlantic | 2 | 22 |
|  | MNCN 16.01/18081 | NO_061 | 9 |  |  |  | Norway, Trondheimsfjorden, Rødberg | 180-250 | 63.468217 | 9.999833 | *Lophelia* | Temp N Atlantic | 2 | 22 |
|  | MNCN 16.01/17995* | 1431_TRO | 9 | MH431261 |  | MH431156 | Norway, Trondheimsfjorden, Rødberg | 180-250 | 63.472667 | 10.000667 | *Lophelia* | Temp N Atlantic | 2 | 22 |
|  | MNCN 16.01/17996* | 1432_TRO | 9 | MH431262 |  | MH431157 | Norway, Trondheimsfjorden, Rødberg | 180-250 | 63.472667 | 10.000667 | *Lophelia* | Temp N Atlantic | 2 | 22 |
|  | MNCN16.01/17997** | 1475_TRO | 9 | MH431263 | MH431080 | MH431158 | Norway, Trondheimsfjorden, Rødberg | 180-250 | 63.472667 | 10.000667 | *Lophelia* | Temp N Atlantic | 2 | 22 |
|  | MNCN 16.01/17998 | 1468_BE | 9 | MH431264 | MH431081 | MH431159 | Norway, Hjeltefjorden, Føllese | 101-125 | 60.41375 | 5.1413 | dead *Lophelia* | Temp N Atlantic | 2 | 22 |
|  | MNCN 16.01/18082 | BE06F008 | 9 |  |  |  | Norway, Hjeltefjorden, Føllese | 101-125 | 60.41375 | 5.1413 | dead *Lophelia* | Temp N Atlantic | 2 | 22 |
|  | MNCN 16.01/17999 | 1474_BE | 9 | MH431265 |  | MH431160 | Norway Hjeltefjorden, Føllese | 101-125 | 60.41375 | 5.1413 | dead *Lophelia* | Temp N Atlantic | 2 | 22 |
|  | MNCN 16.01/18000 | 1476_TRO | 9 | MH431266 |  | MH431161 | Norway, Trondheimsfjorden, Rødberg | 180-250 | 63.472667 | 10.000667 | *Lophelia* | Temp N Atlantic | 2 | 22 |
|  | MNCN 16.01/18001 | 2591_BE | 9 | MH431267 | MH431082 | MH431162 | Norway, Bergen, Korsfjorden | 150-250 | 60.1612 | 5.170817 | *Geodia, Phakellia, Sabella* tubes, *Pandalina* | Temp N Atlantic | 2 | 22 |
| *A.lineata* | MNCN 16.01/18002 | 0976_BA | 10 | MH431268 |  | MH431163 | France, Banyuls | 25 | 42.499 | 3.141 | coralligenous | Temp N Atlantic | 4 | 35 |
|  | MNCN 16.01/18003, 18464 | 0977_BA | 10 | MH431269 |  | MH431164 | France, Banyuls | 25 | 42.499 | 3.141 | coralligenous | Temp N Atlantic | 4 | 35 |
|  | MNCN 16.01/18004, 18465 | 0978_BA | 10 | MH431270 |  | MH431165 | France, Banyuls | 25 | 42.499 | 3.141 | coralligenous | Temp N Atlantic | 4 | 35 |
|  | MNCN 16.01/18005 | 0980_BA | 10 | MH431271 |  | MH431166 | France, Banyuls | 25 | 42.499 | 3.141 | coralligenous | Temp N Atlantic | 4 | 35 |
|  | MNCN 16.01/18006 | 0981_BA | 10 | MH431272 |  | MH431167 | France, Banyuls | 25 | 42.499 | 3.141 | coralligenous | Temp N Atlantic | 4 | 35 |
|  | MNCN 16.01/18007 | 0982_BA | 10 | MH431273 |  | MH431168 | France, Banyuls | 25 | 42.499 | 3.141 | coralligenous | Temp N Atlantic | 4 | 35 |
|  | MNCN 16.01/18008 | 1454_BA | 10 | MH431275 | MH431083 | MH431170 | France, Banyuls | 25 | 42.476667 | 3.161667 | coralligenous | Temp N Atlantic | 4 | 35 |
|  | MNCN 16.01/18083 | BA2001F21 | 10 |  |  |  | France, Banyuls | 25 | 42.476667 | 3.161667 | coralligenous | Temp N Atlantic | 4 | 35 |
|  | MNCN 16.01/18009 | 1082_CR | 10 | MH431274 |  | MH431169 | Croatia, Istria, off Rovinj | 27 | 45.087 | 13.608617 | shells, ascidians, sponges etc. | Temp N Atlantic | 4 | 30 |
|  | MNCN 16.01/18010 | 1751_IT | 10 | MH431278 |  | MH431173 | Italy, Ischia | 15 | 40.74475 | 13.978333 | coralligenous and well sorted sand | Temp N Atlantic | 4 | 35 |
|  | MNCN 16.01/18011, 18466 | 1746_IT | 10 | MH431276 |  | MH431171 | Italy, Ischia | 15 | 40.74475 | 13.978333 | as above | Temp N Atlantic | 4 | 35 |
|  | MNCN 16.01/18012 | 1748_IT | 10 | MH431277 |  | MH431172 | Italy, Ischia | 15 | 40.74475 | 13.978333 | as above | Temp N Atlantic | 4 | 35 |
| *A.* sp. 5 | MNCN 16.01/180013 | 1436_CH | 11 | MH431193 | MH431069 |  | Chile, Coquimbo area | intertidal | −29.953333 | −71.363 |  | Temperate South America | 45 | 177 |
| *A. emilioi* | MNCN 16.01/18014* | 1433_CH | 12 | MH431190 |  |  | Chile, Coquimbo area | intertidal | −29.953333 | −71.363 |  | Temperate South America | 45 | 177 |
|  | MNCN 16.01/18463* | 1434_CH | 12 | MH431191 | MH431065 |  | Chile, Coquimbo area | intertidal | −29.953333 | −71.363 |  | Temperate South America | 45 | 177 |
|  | MNCN 16.01/18016* | 1435_CH | 12 | MH431192 | MH431066 |  | Chile, Coquimbo area | intertidal | −29.953333 | −71.363 |  | Temperate South America | 45 | 177 |
|  | MNCN 16.01/18017* | 1437_CH | 12 | MH431194 | MH431067 | MH431115 | Chile, Coquimbo area | 5-7 | −29.966167 | −71.352833 | among *Chaetopoterus*-tubes | Temperate South America | 45 | 177 |
|  | MNCN 16.01/18018** | 1438_CH | 12 | MH431195 | MH431068 | MH431116 | Chile, Valdivia area | intertidal | −39.824347 | −73.407194 |  | Temperate South America | 45 | 178 |
| *A. rhombeata* | MNCN 16.01/18019 | 2151_FL | 13 | MH431196 | MH431070 | MH431098 | USA, Florida Keys, Summerland Key, north point | intertidal | 24.686483 | −81.4439 | under bridge, *Caulerpa* | Tropical Atlantic | 12 | 70 |
|  | MNCN 16.01/18020 | 2152_FL | 13 | MH431197 | MH431072 | MH431099 | USA, Florida Keys, Summerland Key, north point | intertidal | 24.686483 | −81.4439 | under bridge, *Caulerpa* | Tropical Atlantic | 12 | 70 |
|  | MNCN 16.01/18021 | 2153_FL | 13 | MH431198 | MH431071 |  | USA, Florida Keys, Summerland Key, north point | intertidal | 24.686483 | −81.4439 | under bridge, *Caulerpa* | Tropical Atlantic | 12 | 70 |
|  | MNCN 16.01/18022 | 2157_FL | 13 | MH431199 | MH431073 | MH431100 | USA, Florida Keys, Summerland Key, north point | intertidal | 24.686483 | −81.4439 | under bridge, *Caulerpa* | Tropical Atlantic | 12 | 70 |
|  | MNCN 16.01/18023 | 2158_FL | 13 | MH431200 | MH431074 | MH431101 | USA, Florida Keys, Summerland Key, north point | intertidal | 24.686483 | −81.4439 | under bridge, *Caulerpa* | Tropical Atlantic | 12 | 70 |
|  | MNCN 16.01/18024 | 2159_FL | 13 | MH431201 | MH431075 | MH431102 | USA, Florida Keys, Summerland Key, north point | intertidal | 24.686483 | −81.4439 | under bridge, *Caulerpa* | Tropical Atlantic | 12 | 70 |
|  | MNCN16.01/18025 | 2601_CBC | 13 | MH431202 | MH431076 | MH431103 | Belize, Carrie Bow Cay (Ellen Cay) | 10 | 16.803167 | −88.077817 | coarse sand | Tropical Atlantic | 12 | 68 |
| *A. hectori* n. sp. | MNCN 16.01/18026 | 2593_CA | 14 | MH431244 | MH431063 | MH431096 | USA, California, San Diego, La Jolla | 18 | 32.806117 | −117.28635 | *Macrocystis* holdfast | Temperate Northern Pacific | 11 | 59 |
|  | MNCN 16.01/18027 | 2594_CA | 14 | MH431245 | MH431064 | MH431097 | USA, California, San Diego, La Jolla | 18 | 32.806117 | −117.28635 | *Macrocystis* holdfast | Temperate Northern Pacific | 11 | 59 |
| *A. anae* n. sp | MNCN 16.01/18028* | 1425_WA | 15 | MH431240 | MH431060 | MH431092 | USA, Washington State, Friday Harbor Laboratory | 0-1 | 48.5455 | −123.0125 | epifauna on floating dock outside FHL, barnacles with associated fauna, yellow sponge, hydroids | Temperate Northern Pacific | 10 | 57 |
|  | MNCN 16.01/18029** | 1448_WA | 15 | MH431241 | MH431062 | MH431093 | USA, Washington State, Friday Harbor Laboratory | 0-1 | 48.5455 | −123.0125 | as above | Temperate Northern Pacific | 10 | 57 |
|  | MNCN 16.01/18030*, 18463 | 2595_WA | 15 | MH431243 |  | MH431095 | USA, Washington State, Friday Harbor Laboratory | 70-75 | 48.4935 | −122.9485 | *Polycarpa*, barnacles with rich associated fauna, few sponges | Temperate Northern Pacific | 10 | 57 |
|  | MNCN 16.01/18031*, 18467 | 2166_WA | 15 | MH431242 | MH431061 | MH431094 | USA, Washington State, Friday Harbor Laboratory | 70-75 | 48.4935 | −122.9485 | as above | Temperate Northern Pacific | 10 | 57 |
| *A. nigrolineata* | MNCN 16.01/18032 | 0199_CA | 16 | MH431246 |  | MH431104 | USA, California, Los Angeles, harbour | 0-1 | 33.75 | −118.266667 | epifauna on floating docks and impounded ships | Temperate Northern Pacific | 11 | 59 |
|  | MNCN 16.01/18033 | 2599_CA | 16 | MH431255 | MH431058 | MH431113 | USA, California, Los Angeles, harbour | 0-1 | 33.75 | −118.266667 | as above | Temperate Northern Pacific | 11 | 59 |
|  |  | 1420_CA | 16 | MH431247 | MH431053 | MH431105 | USA, California, Los Angeles, harbour | 0-1 | 33.75 | −118.266667 | as above | Temperate Northern Pacific | 11 | 59 |
|  | MNCN 16.01/18035 | 1452_CA | 16 | MH431253 | MH431057 | MH431111 | USA, California, Los Angeles, harbour | 0-1 | 33.75 | −118.266667 | as above | Temperate Northern Pacific | 11 | 59 |
|  | MNCN 16.01/18036 | 1453_CA | 16 |  |  |  | USA, California, Los Angeles, harbour | 0-1 | 33.75 | −118.266667 | as above | Temperate Northern Pacific | 11 | 59 |
|  | MNCN 16.01/18037 | 1421_CA | 16 | MH431248 | MH431054 | MH431106 | USA, California, Los Angeles, harbour | 0-1 | 33.75 | −118.266667 | as above | Temperate Northern Pacific | 11 | 59 |
|  | MNCN 16.01/18038, 18468 | 2597_CA | 16 | MH431254 |  | MH431112 | USA, California, San Diego, Harbour | 0-1 | 32.724667 | −117.223 | epifauna on dock | Temperate Northern Pacific | 11 | 59 |
|  | MNCN 16.01/18039 | 1423_CA | 16 | MH431249 | MH431059 | MH431107 | USA, California, San Diego, Harbour | 0-1 | 32.724667 | −117.223 | epifauna on dock | Temperate Northern Pacific | 11 | 59 |
|  | MNCN 16.01/18040, 18469 | 1451_CA | 16 | MH431252 | MH431056 | MH431110 | USA, California, San Diego, Harbour | 0-1 | 32.724667 | −117.223 | epifauna on dock | Temperate Northern Pacific | 11 | 59 |
|  | MNCN 16.01/18041 | 1424_CA | 16 | MH431250 |  | MH431108 | USA, California, San Diego, La Jolla | intertidal | 32.842667 | −117.281 | intertidal flat with surfgrass (*Phyllospadix* sp.) | Temperate Northern Pacific | 11 | 59 |
|  | MNCN 16.01/18042 | 2602_CA | 16 |  |  | MH431114 | USA, California, San Diego, La Jolla | intertidal | 32.842667 | −117.281 | as above | Temperate Northern Pacific | 11 | 59 |
|  | MNCN 16.01/18043 | 1450_CA | 16 | MH431251 | MH431055 | MH431109 | USA, California, San Diego, La Jolla | intertidal | 32.842667 | −117.281 | as above | Temperate Northern Pacific | 11 | 59 |
| *A. plectorhyncha* | MNCN 16.01/18044 | 1426_CR | 17 | MH431239 |  | MH431147 | Croatia, Istria, off Rovinj | 27 | 45.087 | 13.608617 | shells, ascidians, sponges etc. | Temp N Atlantic | 4 | 30 |
| *A. idae* n. sp. | MNCN 16.01/18045 | 1417_BA | 18 | MH431233 |  | MH431134 | France, Banyuls | 0-1 | 42.480167 | 3.135333 | mussels with epifauna | Temp N Atlantic | 4 | 35 |
|  | MNCN 16.01/18046 | 1455_BA | 18 | MH431234 |  | MH431135 | France, Banyuls | 0-1 | 42.480167 | 3.135333 | mussels with epifauna | Temp N Atlantic | 4 | 35 |
|  | MNCN 16.01/18047* | 1752_IT | 18 | MH431237 | MH431051 | MH431137 | Italy, Capri | 0.5 | 40.55665 | 14.245333 | growth on floating jetty | Temp N Atlantic | 4 | 35 |
|  | MNCN 16.01/18048* | 1753_IT | 18 | MH431238 | MH431052 | MH431138 | Italy, Capri | 0.5 | 40.55665 | 14.245333 | growth on floating jetty | Temp N Atlantic | 4 | 35 |
|  | MNCN 16.01/18049* | 1749_IT | 18 | MH431235 | MH431050 | MH431136 | Italy, Capri | 15 | 40.74475 | 13.978333 | coralligenous and well sorted sand | Temp N Atlantic | 4 | 35 |
|  | MNCN 16.01/18050** | 1750_IT | 18 | MH431236 |  |  | Italy, Capri | 15 | 40.74475 | 13.978333 | as above | Temp N Atlantic | 4 | 35 |
| *A. spectabilis* | MNCN 16.01/18051 | 0077_SP | 19 | MH431224 | MH431038 | MH431140 | Spain, Cadiz |  | 36.383333 | −6.216667 | floating docks, very rich fauna with see squirts in dense aggregations, hydroids, bryozoans (*Bugula*), sponges. | Temp N Atlantic | 4 | 27 |
|  | MNCN 16.01/18052 | 0984_BA | 19 | MH431225 |  | MH431141 | France, Banyuls | 25 | 42.499 | 3.141 | coralligenous | Temp N Atlantic | 4 | 35 |
|  | MNCN 16.01/18053 | 0985_BA | 19 | MH431226 |  | MH431142 | France, Banyuls | 25 | 42.499 | 3.141 | coralligenous | Temp N Atlantic | 4 | 35 |
|  | MNCN 16.01/18054 | 2596_BA | 19 | MH431232 |  | MH431146 | France, Banyuls | 25 | 42.499 | 3.141 | coralligenous | Temp N Atlantic | 4 | 35 |
|  | MNCN 16.01/18055 | 1418_BA | 19 | MH431230 |  | MH431145 | France, Banyuls | 0-1 | 42.480167 | 3.135333 | mussels with epifauna | Temp N Atlantic | 4 | 35 |
|  | MNCN 16.01/18056 | 0011_BA | 19 | MH431223 | MH431037 | MH431139 | France, Banyuls | 0-1 | 42.480167 | 3.135333 | mussels with epifauna | Temp N Atlantic | 4 | 35 |
|  | MNCN 16.01/18057 | 1088_CR | 19 | MH431229 |  | MH431144 | Croatia, Istria, off Rovinj | 5-15 | 45.045917 | 13.6237 | lime rock with epifauna and algae | Temp N Atlantic | 4 | 30 |
|  | MNCN 16.01/18058 | 1073_CR | 19 | MH431227 |  | MH431143 | Croatia, Istria, off Rovinj | 5-15 | 45.045917 | 13.6237 | as above | Temp N Atlantic | 4 | 30 |
|  | MNCN 16.01/18059 | 1083_CR | 19 | MH431228 |  |  | Croatia, Istria, off Rovinj | 27 | 45.087 | 13.608617 | shells, ascidians, sponges etc. | Temp N Atlantic | 4 | 30 |
|  | MNCN 16.01/18060 | 1747_IT | 19 | MH431231 |  |  | Italy, Ischia | 15 | 40.74475 | 13.978333 | coralligenous and well sorted sand | Temp N Atlantic | 4 | 35 |
|  | MNCN 16.01/18061 | 1084_CR | 19 | MH431203 | MH431039 |  | Croatia, Istria, off Rovinj | 27 | 45.087 | 13.608617 | shells, ascidians, sponges etc. | Temp N Atlantic | 4 | 30 |
|  | MNCN 16.01/18062 | 1439_MA | 19 | MH431204 | MH431040 | MH431117 | Madeira, Funchal | 5-11 | 32.636067 | −16.930583 | rock and boulders | Temp N Atlantic | 3 | 29 |
|  | MNCN 16.01/18063 | 1440_MA | 19 | MH431205 | MH431041 | MH431118 | Madeira, Funchal | 5-11 | 32.636067 | −16.930583 | rock and boulders | Temp N Atlantic | 3 | 29 |
|  | MNCN 16.01/18064 | 1441_MA | 19 | MH431206 | MH431042 | MH431119 | Madeira, Funchal | 5-11 | 32.636067 | −16.930583 | rock and boulders | Temp N Atlantic | 3 | 29 |
|  | MNCN 16.01/18065 | 1442_MA | 19 | MH431207 |  | MH431120 | Madeira, Funchal | 5-11 | 32.636067 | −16.930583 | rock and boulders | Temp N Atlantic | 3 | 29 |
|  | MNCN 16.01/18066 | 1443_MA | 19 | MH431208 | MH431043 | MH431121 | Madeira, Funchal | 5-11 | 32.636067 | −16.930583 | rock and boulders | Temp N Atlantic | 3 | 29 |
|  | MNCN 16.01/18067 | 1444_MA | 19 | MH431209 |  | MH431122 | Madeira, Funchal | 5-11 | 32.636067 | −16.930583 | rock and boulders | Temp N Atlantic | 3 | 29 |
|  | MNCN 16.01/18068 | 1445_MA | 19 | MH431210 |  | MH431123 | Madeira, Funchal | 5-11 | 32.636067 | −16.930583 | rock and boulders | Temp N Atlantic | 3 | 29 |
|  | MNCN 16.01/18069 | 1446_MA | 19 | MH431211 | MH431044 | MH431124 | Madeira, Funchal | 5-11 | 32.636067 | −16.930583 | rock and boulders | Temp N Atlantic | 3 | 29 |
|  | MNCN 16.01/18070 | 1447_MA | 19 | MH431212 |  | MH431125 | Madeira, Funchal | 5-11 | 32.636067 | −16.930583 | rock and boulders | Temp N Atlantic | 3 | 29 |
|  | MNCN 16.01/18071 | 1456_MA | 19 | MH431213 | MH431045 | MH431126 | Madeira, Funchal | 5-11 | 32.636067 | −16.930583 | rock and boulders | Temp N Atlantic | 3 | 29 |
|  | MNCN 16.01/18072 | 1458_MA | 19 | MH431215 |  |  | Madeira, Funchal | 5-11 | 32.636067 | −16.930583 | rock and boulders | Temp N Atlantic | 3 | 29 |
|  | MNCN 16.01/18073 | 1457_MA | 19 | MH431214 | MH431046 | MH431127 | SE Madeira, Caniçal, Cais da Junta | 12-19 | 32.735017 | −16.740467 | rocks and sand | Temp N Atlantic | 3 | 29 |
|  | MNCN 16.01/18074, 18470 | 1459_MA | 19 | MH431216 |  | MH431128 | SE Madeira, Caniçal, Cais da Junta | 12-19 | 32.735017 | −16.740467 | rocks and sand | Temp N Atlantic | 3 | 29 |
|  | MNCN 16.01/18075, 18471 | 1460_MA | 19 | MH431217 | MH431047 | MH431129 | NW Madeira, Porto Moniz | 15-25 | 32.860733 | −17.15175 | rocks, coral and balanids | Temp N Atlantic | 3 | 29 |
|  | MNCN 16.01/18076 | 1463_MA | 19 | MH431218 |  | MH431130 | NW Madeira, Porto Moniz | 15-25 | 32.860733 | −17.15175 | rocks, coral and balanids | Temp N Atlantic | 3 | 29 |
|  | MNCN 16.01/18077, 18472 | 2160_PLY | 19 | MH431219 |  |  | Great Britain, Plymouth, The Sound | 10-15 | 50.358333 | −4.148333 | coarse shell gravel | Temp N Atlantic | 2 | 25 |
|  | MNCN 16.01/18084 | PLY2011 F005 | 19 |  |  |  | Great Britain, Plymouth, The Sound | 10-15 | 50.358333 | −4.148333 | coarse shell gravel | Temp N Atlantic | 2 | 25 |
|  | MNCN 16.01/18085 | PLY2011 F006 | 19 |  |  |  | Great Britain, Plymouth, The Sound | 10-15 | 50.358333 | −4.148333 | coarse shell gravel | Temp N Atlantic | 2 | 25 |
|  | MNCN 16.01/18078 | 2592_PLY | 19 | MH431222 | MH431049 | MH431133 | Great Britain, Plymouth, The Sound | 10-15 | 50.358333 | −4.148333 | coarse shell gravel | Temp N Atlantic | 2 | 25 |
|  | MNCN 16.01/18079, 18473 | 2162_PLY | 19 | MH431220 |  | MH431131 | Great Britain, Plymouth, The Sound | 10-15 | 50.358333 | −4.148333 | coarse shell gravel | Temp N Atlantic | 2 | 25 |
|  | MNCN 16.01/18080 | 2163_PLY | 19 | MH431221 | MH431048 | MH431132 | Great Britain, Plymouth, The Sound | 10-15 | 50.358333 | −4.148333 | coarse shell gravel | Temp N Atlantic | 2 | 25 |
