# Supplementary figures and images for: Species delimitation in Amblyosyllis (Annelida, Syllidae)
Source: PLoS One. 2019 Apr 10;14(4):e0214211. doi: 10.1371/journal.pone.0214211 (PMC6457521; doi:10.1371/journal.pone.0214211)

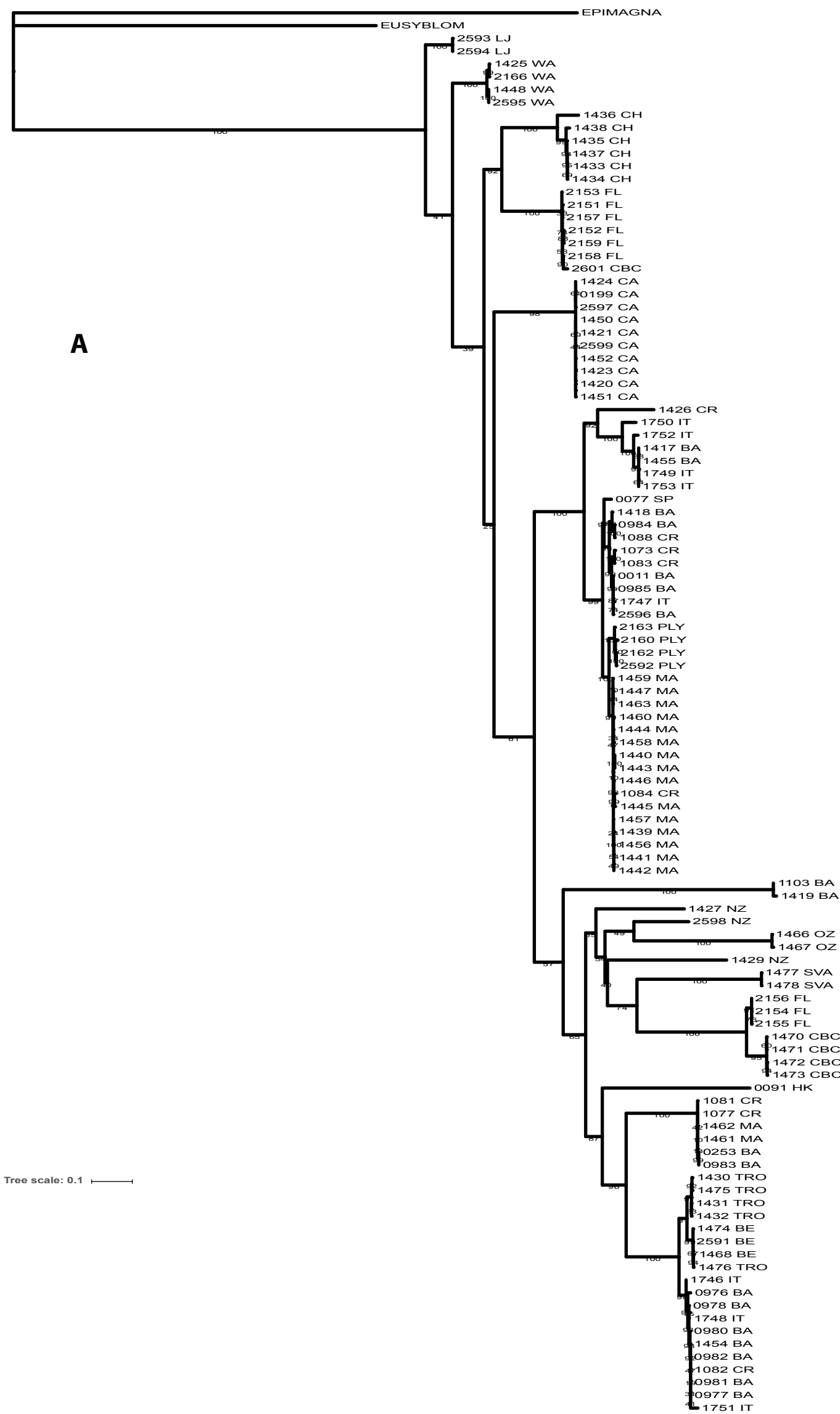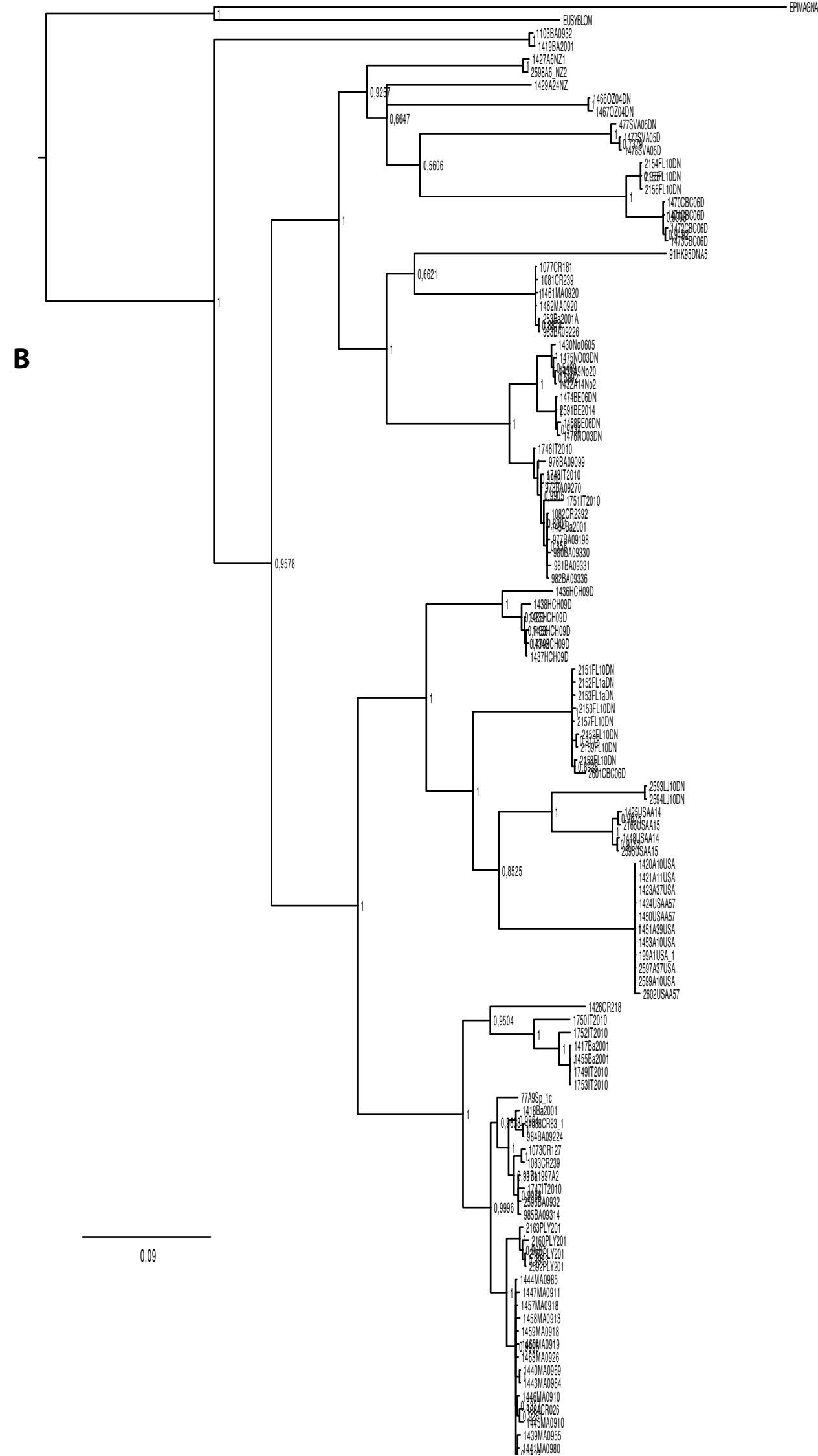

Supplement: S2 File — (A) ML tree obtained from the analyses of the combined mitochondrial data set (COI+6S); Bootstrap support values below nodes. (B) Majority rule consensus tree from BI analysis obtained from the complete combined data set (COI+16S+28S); posterior probability support values close to each node. (PDF) [file pone.0214211.s002.pdf]

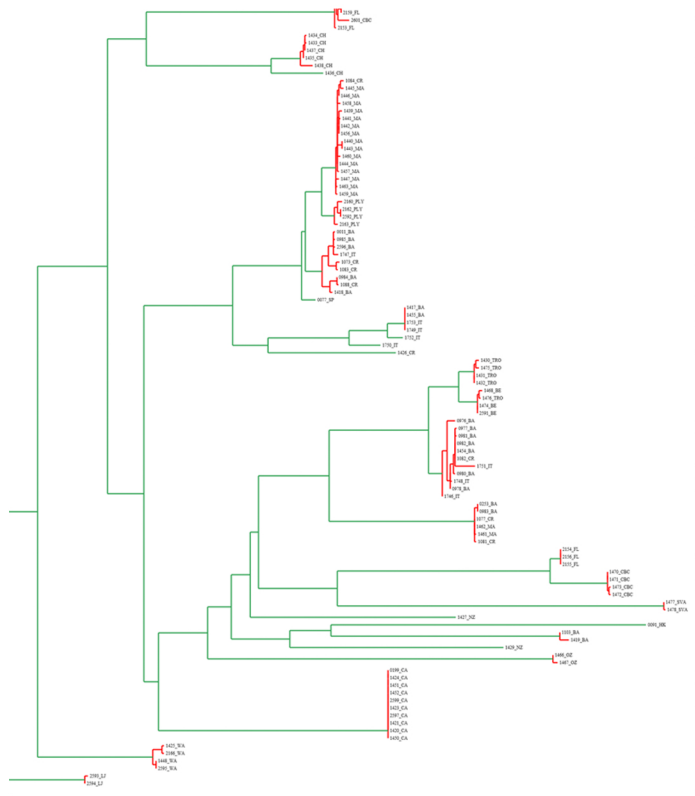

A

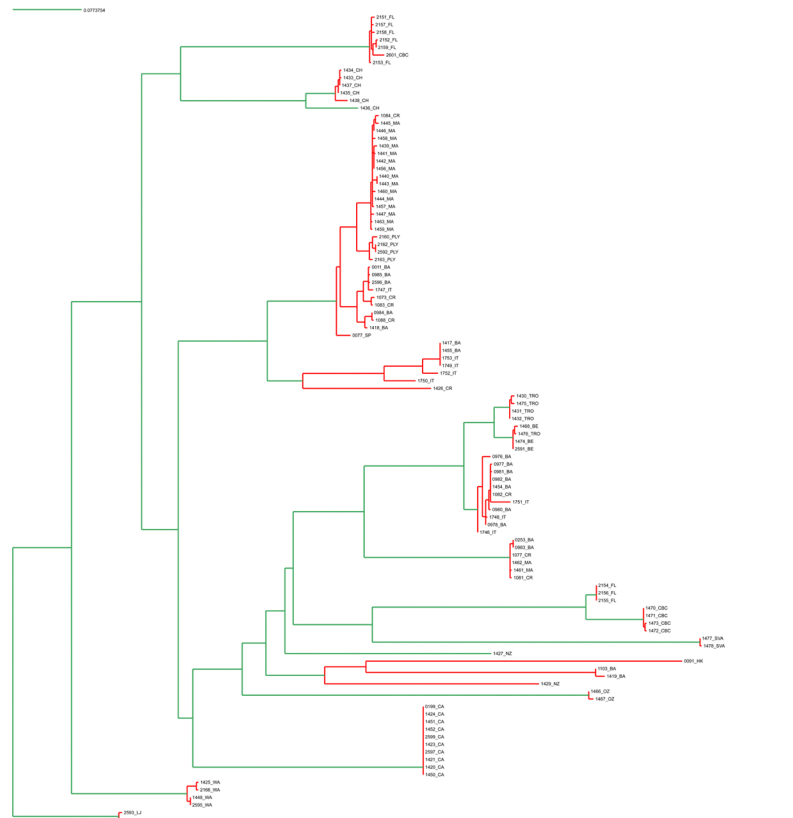

B

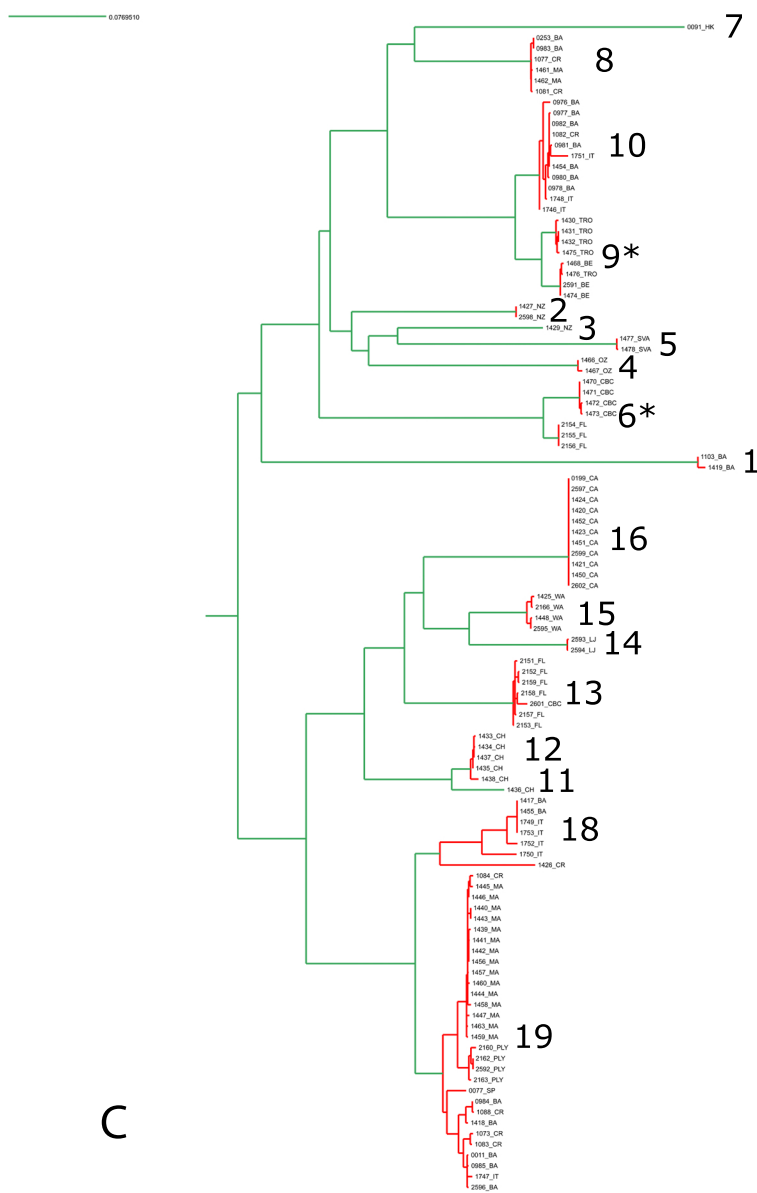

C

Supplement: S6 File — (A) PTP delimitation of COI sequences. Terminal branches in red indicate lineages that stand as separate species and the clades in red are lumped into single species. (B) mPTP delimitation of COI sequences. Terminal branches in red indicate lineages that stand as separate species and the clades in red are lumped into single species. C. mPTP delimitation complete combined data set (COI+16S+28S). Terminal branches in red indicate lineages that stand as separate species and the clades in red are lumped into single species. (PDF) [file pone.0214211.s006.pdf]
