# Supplementary material for: Species delimitation in Amblyosyllis (Annelida, Syllidae)
Source: PLoS One. 2019 Apr 10;14(4):e0214211. doi: 10.1371/journal.pone.0214211 (PMC6457521; doi:10.1371/journal.pone.0214211)
Supplement: S5 File — The analysis included 98 nucleotide sequences. All ambiguous positions were removed for each sequence pair. There was a total of 348 positions in the final dataset (108 of which were parsimony informative). The number of base substitutions per site from averaging over all sequence pairs between clades are shown. Blue numbers indicate low distances (<0.02), between clades. (DOCX) [file pone.0214211.s005.docx]

|  | 15 | 14 | 13 | 16 | 12 | 19 | 18 | 17 | 6 | 5 | 3 | 4 | 2 | 8 | 7 | 9 | 10 | 1 |
| --- | --- | --- | --- | --- | --- | --- | --- | --- | --- | --- | --- | --- | --- | --- | --- | --- | --- | --- |
| 15 | 0 | 0,02 | 0,02 | 0,03 | 0,043 | 0,084 | 0,083 | 0,080 | 0,133 | 0,158 | 0,140 | 0,128 | 0,140 | 0,132 | 0,185 | 0,183 | 0,194 | 0,182 |
| 14 | 0,02 | 0 | 0,03 | 0,039 | 0,048 | 0,094 | 0,093 | 0,090 | 0,136 | 0,158 | 0,139 | 0,127 | 0,139 | 0,133 | 0,186 | 0,175 | 0,189 | 0,185 |
| 13 | 0,02 | 0,03 | 0 | 0,02 | 0,039 | 0,085 | 0,084 | 0,081 | 0,129 | 0,160 | 0,136 | 0,124 | 0,139 | 0,129 | 0,189 | 0,175 | 0,183 | 0,182 |
| 16 | 0,03 | 0,040 | 0,02 | 0 | 0,048 | 0,091 | 0,090 | 0,087 | 0,126 | 0,154 | 0,133 | 0,121 | 0,136 | 0,132 | 0,183 | 0,177 | 0,183 | 0,170 |
| 12 | 0,045 | 0,050 | 0,040 | 0,050 | 0 | 0,073 | 0,072 | 0,069 | 0,135 | 0,148 | 0,136 | 0,124 | 0,136 | 0,123 | 0,174 | 0,175 | 0,176 | 0,175 |
| 19 | 0,090 | 0,103 | 0,091 | 0,099 | 0,078 | 0 | 0,01 | 0 | 0,143 | 0,161 | 0,149 | 0,140 | 0,146 | 0,124 | 0,180 | 0,191 | 0,196 | 0,173 |
| 18 | 0,089 | 0,102 | 0,090 | 0,097 | 0,076 | 0,01 | 0 | 0,01 | 0,143 | 0,156 | 0,144 | 0,135 | 0,138 | 0,122 | 0,176 | 0,188 | 0,193 | 0,176 |
| 17 | 0,086 | 0,098 | 0,086 | 0,093 | 0,073 | 0 | 0,01 | n/c | 0,140 | 0,162 | 0,150 | 0,141 | 0,147 | 0,125 | 0,179 | 0,188 | 0,193 | 0,170 |
| 6 | 0,149 | 0,152 | 0,145 | 0,140 | 0,151 | 0,161 | 0,161 | 0,158 | 0 | 0,090 | 0,072 | 0,063 | 0,084 | 0,093 | 0,113 | 0,102 | 0,107 | 0,152 |
| 5 | 0,181 | 0,181 | 0,185 | 0,176 | 0,169 | 0,185 | 0,178 | 0,187 | 0,096 | 0 | 0,036 | 0,039 | 0,081 | 0,090 | 0,108 | 0,106 | 0,102 | 0,160 |
| 3 | 0,158 | 0,157 | 0,153 | 0,149 | 0,153 | 0,168 | 0,162 | 0,171 | 0,076 | 0,037 | n/c | 0,01 | 0,063 | 0,063 | 0,087 | 0,081 | 0,086 | 0,148 |
| 4 | 0,142 | 0,142 | 0,138 | 0,134 | 0,138 | 0,157 | 0,151 | 0,159 | 0,066 | 0,040 | 0,01 | 0 | 0,054 | 0,060 | 0,090 | 0,079 | 0,084 | 0,136 |
| 2 | 0,156 | 0,157 | 0,156 | 0,152 | 0,152 | 0,164 | 0,155 | 0,166 | 0,089 | 0,086 | 0,066 | 0,056 | 0 | 0,081 | 0,126 | 0,114 | 0,111 | 0,151 |
| 8 | 0,147 | 0,148 | 0,143 | 0,147 | 0,137 | 0,138 | 0,135 | 0,139 | 0,101 | 0,098 | 0,067 | 0,063 | 0,087 | 0 | 0,089 | 0,101 | 0,099 | 0,142 |
| 7 | 0,215 | 0,217 | 0,221 | 0,213 | 0,201 | 0,209 | 0,203 | 0,208 | 0,124 | 0,118 | 0,093 | 0,096 | 0,140 | 0,096 | n/c | 0,124 | 0,116 | 0,180 |
| 9 | 0,212 | 0,201 | 0,201 | 0,205 | 0,201 | 0,224 | 0,220 | 0,220 | 0,111 | 0,115 | 0,086 | 0,084 | 0,124 | 0,110 | 0,138 | 0,014 | 0,054 | 0,149 |
| 10 | 0,226 | 0,221 | 0,211 | 0,211 | 0,204 | 0,232 | 0,227 | 0,228 | 0,116 | 0,110 | 0,092 | 0,089 | 0,121 | 0,108 | 0,129 | 0,057 | 0 | 0,150 |
| 1 | 0,212 | 0,217 | 0,212 | 0,196 | 0,204 | 0,202 | 0,207 | 0,198 | 0,173 | 0,184 | 0,167 | 0,152 | 0,171 | 0,161 | 0,211 | 0,168 | 0,169 | 0 |
